# A comparison of all-cause and cause-specific mortality by household socioeconomic status across seven INDEPTH network health and demographic surveillance systems in sub-Saharan Africa

**DOI:** 10.1080/16549716.2019.1608013

**Published:** 2019-05-16

**Authors:** Matthew M. Coates, Mamusu Kamanda, Alexander Kintu, Iwara Arikpo, Alberto Chauque, Melkamu Merid Mengesha, Alison J. Price, Peter Sifuna, Marylene Wamukoya, Charfudin N. Sacoor, Sheila Ogwang, Nega Assefa, Amelia C. Crampin, Eusebio V. Macete, Catherine Kyobutungi, Martin M. Meremikwu, Walter Otieno, Kafui Adjaye-Gbewonyo, Andrew Marx, Peter Byass, Osman Sankoh, Gene Bukhman

**Affiliations:** a Department of Global Health and Social Medicine, Program in Global Noncommunicable Diseases and Social Change, Harvard Medical School, Boston, USA; b INDEPTH Network, Accra, Ghana; c Department of Global Health and Population, Harvard T.H. Chan School of Public Health, Boston, USA; d Cross River Health & Demographic Surveillance System (CRHDSS), University of Calabar, Calabar, Nigeria; e Centro de Investigação em Saúde da Manhiça (CISM), Mozambique; f College of Health and Medical Sciences, Haramaya University, Harar, Ethiopia; g Department of Population Health, London School of Hygiene & Tropical Medicine, London, UK; h Malawi Epidemiology and Intervention Research Unit, Lilongwe, Malawi; i US Army Medical Research Directorate–Kenya (USAMRD-K)/Kenya Medical Research Institute (KEMRI), Kisumu, Kenya; j African Population and Health Research Center, Nairobi, Kenya; k Department of Paediatrics and Child Health, Maseno University School of Medicine, Kisumu, Kenya; l University College London, London, UK; m Department of Epidemiology and Global Health, Umeå University, Umeå, Sweden; n Medical Research Council/Wits University Rural Public Health and Health Transitions Research Unit (Agincourt), School of Public Health, Faculty of Health Sciences, University of the Witwatersrand, Johannesburg, South Africa; o Institute of Applied Health Sciences, University of Aberdeen, Aberdeen, Scotland; p Statistics Sierra Leone, Freetown, Sierra Leone; q College of Medicine and Allied Health Sciences, University of Sierra Leone, New England, Sierra Leone; r School of Public Health, Faculty of Health Sciences, University of the Witwatersrand, Johannesburg, South Africa; s Division of Global Health Equity, Brigham and Women’s Hospital, Boston, MA, USA; t Partners In Health, Boston, MA, USA

**Keywords:** Cause of death, verbal autopsy, non-communicable disease, life expectancy

## Abstract

**Background**: Understanding socioeconomic disparities in all-cause and cause-specific mortality can help inform prevention and treatment strategies.

**Objectives**: To quantify cause-specific mortality rates by socioeconomic status across seven health and demographic surveillance systems (HDSS) in five countries (Ethiopia, Kenya, Malawi, Mozambique, and Nigeria) in the INDEPTH Network in sub-Saharan Africa.

**Methods**: We linked demographic residence data with household survey data containing living standards and education information we used to create a poverty index. Person-years lived and deaths between 2003 and 2016 (periods varied by HDSS) were stratified in each HDSS by age, sex, year, and number of deprivations on the poverty index (0–8). Causes of death were assigned to each death using the InterVA-4 model based on responses to verbal autopsy questionnaires. We estimated rate ratios between socioeconomic groups (2–4 and 5–8 deprivations on our poverty index compared to 0–2 deprivations) for specific causes of death and calculated life expectancy for the deprivation groups.

**Results**: Our pooled data contained almost 3.5 million person-years of observation and 25,038 deaths. All-cause mortality rates were higher among people in households with 5–8 deprivations on our poverty index compared to 0–2 deprivations, controlling for age, sex, and year (rate ratios ranged 1.42 to 2.06 across HDSS sites). The poorest group had consistently higher death rates in communicable, maternal, neonatal, and nutritional conditions (rate ratios ranged 1.34–4.05) and for non-communicable diseases in several sites (1.14–1.93). The disparities in mortality between 5–8 deprivation groups and 0–2 deprivation groups led to lower life expectancy in the higher-deprivation groups by six years in all sites and more than 10 years in five sites.

**Conclusions**: We show large disparities in mortality on the basis of socioeconomic status across seven HDSS in sub-Saharan Africa due to disparities in communicable disease mortality and from non-communicable diseases in some sites. Life expectancy gaps between socioeconomic groups within sites were similar to the gaps between high-income and lower-middle-income countries. Prevention and treatment efforts can benefit from understanding subpopulations facing higher mortality from specific conditions.

## Background

Extensive studies in high-income countries have described associations between socioeconomic status (SES) and both overall and cause-specific disease burden, often exploring social, biological, and psychological pathways that underlie these disparities [1–3]. Efforts to increase evidence on inequalities within low- and lower-middle-income countries (LLMICs) are growing [4]. Studies from LLMICs have shown socioeconomic disparities in all-cause mortality across the age spectrum, but these data are mostly based on cross-sectional population surveys and more commonly describe inequalities in child and neonatal mortality [5–7]. Evidence from studies investigating disparities in SES and cause-specific death patterns and rates in these populations is limited, particularly across multiple causes and age groups [8–10].

In the era of the Sustainable Development Goals (SDGs), there have been increasing calls for more geographically specific data and data that can be stratified by subpopulations to address health equity [11,12]. The lack of national vital registration and disease registries in LLMICs means population-level data on causes of death (COD) to study disparities by SES are generally unavailable. Nonetheless, many LLMICs have well-established health and demographic surveillance system (HDSS) sites that track individuals within a geographically defined area over time. These sites systematically collect information on sociodemographic factors, vital events (births and deaths), and COD (using a verbal autopsy approach). Importantly, within HDSS sites, individual-level data, including date and COD, can be linked to household-level demographic data and other information collected by specific studies nested within the site [13]. Previously, the INDEPTH Network, a collaborative group of 50 HDSS sites of which 75% are located in Africa, has pooled verbal autopsy data from member HDSS sites to describe patterns in COD across Africa and Asia [14,15].

Estimates of patterns of COD from verbal autopsies in HDSS sites have contributed to understanding of global patterns of COD [14,16]. Multi-site analyses have described the epidemiological transition of changing patterns of COD over time, associated with changing socioeconomic and demographic factors [17–19]. HDSS sites have contributed to a growing evidence base on health equity in LLMICs [13], but the degree to which levels and patterns of COD vary by SES groups within communities in LLMICs is underexplored. Studies examining household-level or individual-level socioeconomic factors and cause-specific death rates have often been limited to single sites and particular health conditions. Such studies have often shown higher death rates from some causes, including HIV, malaria, tuberculosis, maternal conditions, childhood infection, and noncommunicable diseases (NCDs) associated with lower attainment on certain SES indicators [9,10,20–24]. Nonetheless, other studies report conflicting or inconclusive evidence about the same causes [8,25,26]. Further analyses using these types of data offer an opportunity to monitor health equity around global goals such as the SDGs and to understand how phenomena such as the epidemiological transition affect populations with different levels of SES. This study utilized verbal autopsy data coupled with data on household living standards and education from seven HDSS sites in sub-Saharan Africa (SSA) in the INDEPTH Network to examine patterns in rates of all-cause and cause-specific mortality by levels of absolute poverty.

## Methods

### Data

Our study included seven HDSS sites from the INDEPTH Network with varied levels of extreme poverty, verbal autopsy data for deceased individuals, and linked measures of household SES. All contributing HDSS sites were in SSA – Harar and Kersa, Ethiopia; Kombewa and Nairobi, Kenya; Karonga, Malawi; Manhiça, Mozambique; and Cross River, Nigeria. In addition to routine demographic information (births, in-migration, out-migration, and deaths), these sites also periodically collected household data on education and living standards, in specific household surveys or as part of routine data collection. Profiles for five sites are published elsewhere [27–31]. We included all deaths and person-time lived in the site among residents during the site-specific time period of analysis. Site characteristics such as the time period of analysis, total person-years, and urbanicity are shown in Table 1. It is important to note that the sites in large cities, such as in Nairobi and Harar, are in particular areas of the cities. We separated the data from the Nairobi site into two time periods (2003–2009 and 2010–2015) because of the long time series of available data. While this reduced the number of pooled observations, we chose not to pool over the long time period given the changing death rates and poverty rates over time, as well as the possibility that the associations between death rates and poverty changed over the 13-year period.

**Table 1.** Characteristics of health and demographic surveillance systems (HDSS): setting, demographics, mortality, and poverty.

	Ethiopia		Kenya			Malawi	Mozambique	Nigeria
	Harar	Kersa	Kombewa	Nairobi	Nairobi	Karonga	Manhiça	Cross River
Period of Analysis	2013–2016	2013–2016	2011–2015	2003–2009	2010–2015	2009–2016	2010–2016	2013–2016
Urbanicity	Urban	Rural	Rural	Urban	Urban	Rural	Rural	Urban/Rural
Person-Years (thousands)	163	351	845	407	400	298	892	103
Percent of Population By Age Range								
Under 5	9.7	14.9	14.3	14.6	13.5	17.0	16.1	9.5
5 to 14	20.3	30.4	30.7	17.2	19.1	30.3	29.5	24.9
15 to 39	48.4	37.4	39.8	55.4	52.3	36.6	36.4	44.7
40 to 59	15.4	13.3	9.4	11.3	13.2	10.7	11.3	16.0
60 and over	6.2	4.0	5.9	1.5	1.9	5.3	6.7	4.9
Percent of Population Female	52.4	49.4	53.6	43.6	44.7	51.8	55.5	50.6
Deaths	524	2388	5007	3226	2496	1834	9197	366
Crude Death Rate (per 100,000 person-years)	321	680	593	792	624	615	1030	354
Age-Sex-Standardized Death Rate (per 100,000 person-years)	304	825	681	941	762	646	1069	482
Percent of Population by SES Deprivations (2.5^th^-97.5^th^ Percentile)								
0–2 Deprivations	70.6 (70.4–70.8)	2.0 (1.9–2.0)	3.5 (3.5–3.5)	40.5 (40.3–40.6)	65.6 (65.6–65.7)	8.6 (8.5–8.7)	42.2 (42.2–42.3)*	60.4 (60.4–60.4)
3–4 Deprivations	28.1 (28.0–28.3)	16.7 (16.5–16.8)	31.2 (31.2–31.2)	50.6 (50.4–50.8)	31.8 (31.8–31.9)	54.8 (54.6–55.0)	44.5 (44.5–44.6)*	27.7 (27.7–27.7)
5–8 Deprivations	1.2 (1.2–1.3)	81.3 (81.2–81.5)	65.4 (65.4–65.4)	8.9 (8.8–9.0)	2.6 (2.5–2.6)	36.6 (36.5–36.8)	13.2 (13.1–13.3)*	11.9 (11.9–11.9)
Median SES Deprivations (mean)	2 (2.0)	6 (5.5)	5 (4.9)	3 (2.9)	2 (2.2)	4 (4.1)	3 (2.9)*	2 (2.2)

SES = socioeconomic status. *Deprivations reported out of eight possible except for in Manhiça, where reported out of six possible deprivations (two education indicators excluded).

We used a modified version of the Multidimensional Poverty Index (MPI) developed by the Oxford Poverty and Human Development Initiative [32]. Our modified poverty index (PI), used by the *Lancet* Commission on Reframing Noncommunicable Diseases and Injuries for the Poorest Billion (*Lancet* NCDI Poverty Commission), contained eight indicators in which households were either categorized as deprived or not deprived, including school attendance for all school-aged children in the household, maximum years of schooling of any household member over five years, electricity availability, improved water and sanitation availability, flooring material, cooking fuel used, and a set of assets (specific definitions in appendix Table S1) [33]. Deprivations in health on the traditional MPI were excluded from our index because we examined health outcomes. Those individuals living in households deprived in five or more of eight of these indicators were defined to be among the world’s poorest billion people by the Commission [33].

We used a single absolute index to measure poverty for direct comparison of comparable levels of poverty across multiple sites in varied settings. While site-specific indices can be optimized to create evenly split populations (for example, into wealth quintiles), we chose to define SES groups in the same manner across sites. In a few cases, adaptations had to be made to the PI because of inconsistencies in the data collected by each site. For example, no data were collected in the Kersa HDSS on whether households had motorcycles, requiring that asset be left out of the definition of the asset indicator for the Kersa PI. With these adjustments, Manhiça was the only site in which we did not have a full set of eight indicators; the education indicators could not be created from the available data. The Manhiça PI contained six indicators instead of eight (complete details on indicators and assumptions in appendix, Tables S1 & S2). We reported results by number of deprivations.

### Analysis

For cause-specific mortality analyses, individuals contributed exposure time during their residence in the HDSS from the start of the site-specific study period, or their date of in-migration if later, until the earliest of death, out-migration, or last HDSS follow-up date. Returning and repeat migrants only contributed exposure time while resident in the HDSS area. To stratify deaths and exposure time among residents of the sites by age, sex, and the number of indicators in which a person was deprived, we first merged household deprivation data with residency data. The range of years with SES data available varied across sites (full details in appendix). In Nairobi, Karonga, and Manhiça, multiple surveys collected SES data longitudinally. We utilized these surveys over time to reflect the changing poverty status of the household. We used multiple imputation to impute values for the PI indicators for households missing years of data as well as households with no data, using household characteristics such as number of household members, ages of household members, moves in and out of the household, and deaths [34] in the household; neighborhood effects; household effects; and year [35]. In Cross River, Kombewa, Kersa, and Harar, households generally had one survey with SES data in a single baseline year, with new households receiving the survey when they formed. For households in the Kersa and Harar sites with missing survey information, we imputed the indicators using multiple imputation, utilizing the household characteristics described above, though only for a single survey time point per household. In Cross River and Kombewa, we assumed the SES survey data to be representative for the corresponding age and sex groups in the sites to assign population to SES groups. We imputed SES data for the deaths that were missing SES data (less than 5%) using the deaths that had linked SES estimates. For these sites with one poverty index time point per household at a baseline year, that poverty index value was assigned to the person-time and deaths in the household in subsequent years. Full imputation description and sensitivity analyses are shown in the appendix, including a comparison of results using time-variant and time-invariant poverty index values in sites with multiple surveys. We created 20 imputed datasets to carry forward uncertainty from SES assignment through the analysis.

Causes of death were determined by verbal autopsies, which consist of standardized interviews with relatives or witnesses about the symptoms of the deceased individual and circumstances leading to the death. From the answers to these questions, causes of death are typically assigned using computer models or physician review. To improve comparability, we used the InterVA-4 model (version 4.03 or 4.04) to classify COD from verbal autopsy in every site [14,36]. InterVA-4 is a free public software that uses Bayesian probabilistic modeling to assign likelihoods to causes of death based on the coded responses to verbal autopsy questionnaires. As per convention with InterVA, we assigned death fractions based on the likelihoods of the resulting cause classifications, as well as residual likelihood in the ‘indeterminate’ cause category. Deaths determined to be stillbirths were not included. We calculated cause-specific mortality rates (CSMR) using the stratified person-years (PY) and COD for each age group (*a*), sex (*s*), year (*y*), and number of deprivations (*d*), by cause (*c*).

$$CSMR_{c,a,s,y,d}=\frac{Deaths_{c,a,s,y,d}}{PY_{a,s,y,d}}$$

We calculated these age-specific mortality rates for ages under-one year, one to four years, and five-year age groups to 85 years and older. We used these age-sex-specific mortality rates, along with the INDEPTH 2013 population standard for SSA to calculate age-sex-standardized death rates to describe epidemiological differences between sites and SES groups [37]. From these age-sex-standardized cause-specific mortality rates, we also calculated proportions from each cause. To describe the effect of demographic composition across sites, notably age differences, we investigated crude rates. We calculated life expectancies by site and deprivation group using standard life table methods (see appendix) [38]. For summary measures, we present results stratified into three groups based on our poverty index: 0–2 deprivations, 3–4 deprivations, and 5–8 deprivations. While we created results for the most specific causes of death classified by InterVA-4, we additionally created summary results using broader categories (full list of cause classifications in appendix).

To incorporate uncertainty from the missing socioeconomic data, we calculated mortality rates based on each of the imputed datasets we created for each site. Our figures show means across the imputations. In many cases, the additional uncertainty is small, as in the proportion of the population with five or more deprivations (Table 1). We tested for differences in rates of death by cause groups by constructing negative binomial models of deaths for each site and cause, including age group, sex, year, and socioeconomic group as covariates. We took 500 draws from the distribution of estimated coefficients for the regression from each of the 20 imputations, yielding 10,000 simulations of the coefficients, which we summarized using the mean and 95% uncertainty interval (UI), taking the 2.5^th^ and 97.5^th^ percentiles. We considered estimates of relative rates with the UI excluding one to be significant. We also conducted these regressions for all ages combined. To characterize variation in the relative disparities by sex and age, we also conducted similar regressions, stratifying by sex, and then by three broad age groups (under-15, 15 to 39, and over 40), controlling for each of the other factors in each case (results presented in appendix). For more stable results given the smaller numbers of deaths, we limited these stratified analyses to deaths from all causes.

Analyses were conducted using Stata version 15.1 and R version 3.3.3. Multiple imputation was conducted using the Amelia II package in R [35].

## Results

We compiled 45 site-years of verbal autopsy and demographic data from the seven HDSS sites in five countries, comprising almost 3.5 million person-years of observation and 25,038 deaths (Table 1). All sites had young populations, with 83.5% of the population below 40, ranging from 78.4% in Harar to 87.2% in Nairobi from 2003 to 2009. Additionally, 14.6% of the population was below the age of five, ranging from 9.5% in Cross River to 17.0% in Karonga. The population in Kersa HDSS had the highest median number of deprivations (6), while the populations in Harar, Nairobi 2010–2015, and Cross River had the lowest (2). Age and sex structures of the populations by SES level were relatively consistent, though there were some differences (appendix Figures S1-S3). In most sites, young adults around ages 20 to 40 contributed a smaller proportion of the population in the poorer groups than the wealthier groups. The HDSS in Nairobi in particular had a unique pattern with a relatively large number of males between 20 and 39. In several sites, the proportion of the population from women of older ages and from young children was higher in the 5–8 deprivation group than in the wealthier groups, though the overall population of women at older ages was small.

**Figure 1.**
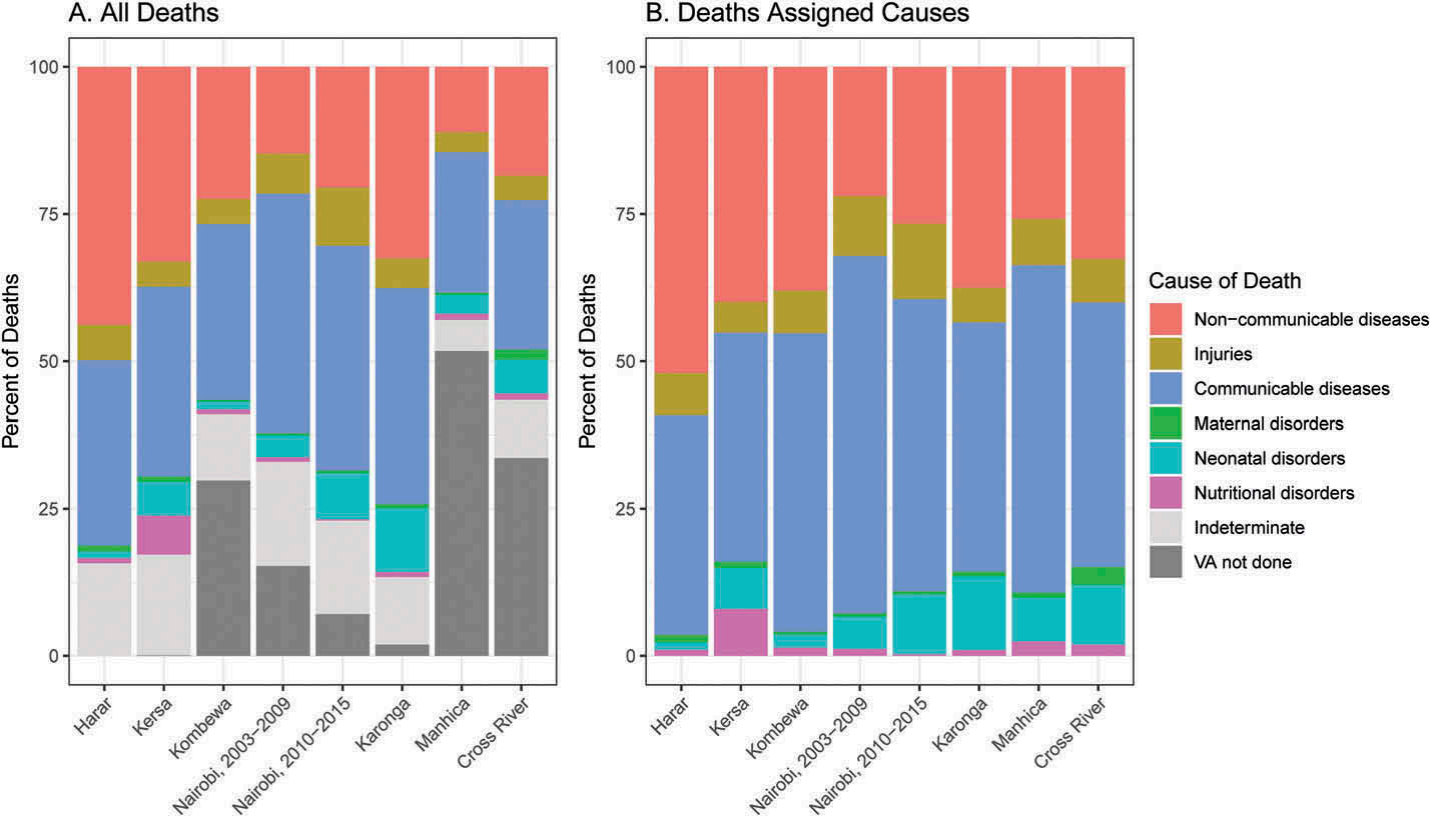
Age-sex-standardized proportions of broad causes of death among deaths among (a) all deaths and (b) deaths assigned causes by InterVA. Note: Countries in which sites are located: Ethiopia (Harar, Kersa), Kenya (Kombewa, Nairobi), Malawi (Karonga), Mozambique (Manhiça), Nigeria (Cross River).

We observed the highest age-sex-standardized mortality rates in Manhiça HDSS (1,069 deaths per 100,000 person-years) and the 2003–2009 period of Nairobi HDSS (941 deaths per 100,000 person-years) sites, and the lowest in Harar (304 per 100,000 person-years) and Cross River (482 per 100,000 person-years) (Table 1). The Manhiça (51.7%), Cross River (38.5%), and Kombewa (30.1%) sites had the highest proportion of deaths that did not have valid VA data collected. All other sites had valid VA data for 85%-100% of recorded deaths. Among deaths with collected VA and for which causes could be assigned to a specific condition (not indeterminate) by the InterVA model, the age-sex-standardized percent from communicable, maternal, neonatal, and nutritional (CMNN) disorders ranged from 67.9% in Nairobi HDSS (2003–2009) to 40.8% in Harar HDSS, while 21.9% to 52.0% of deaths were from NCDs in those same two sites, respectively (Figure 1). The lowest age-sex-standardized proportions of deaths from injuries were in Karonga (5.9%) and Kersa (5.2%), both rural, while the highest were in the urban Nairobi HDSS in the two time periods, with 10.1% from 2003 to 2009 and 12.5% in 2010 to 2015. Crude results for all ages were similar, though the relative percentage of deaths from CMNN conditions was lower in Cross River HDSS, and the relative percentages from injuries and CMNN conditions in Nairobi HDSS were higher (appendix Figure S4).

**Figure 2.**
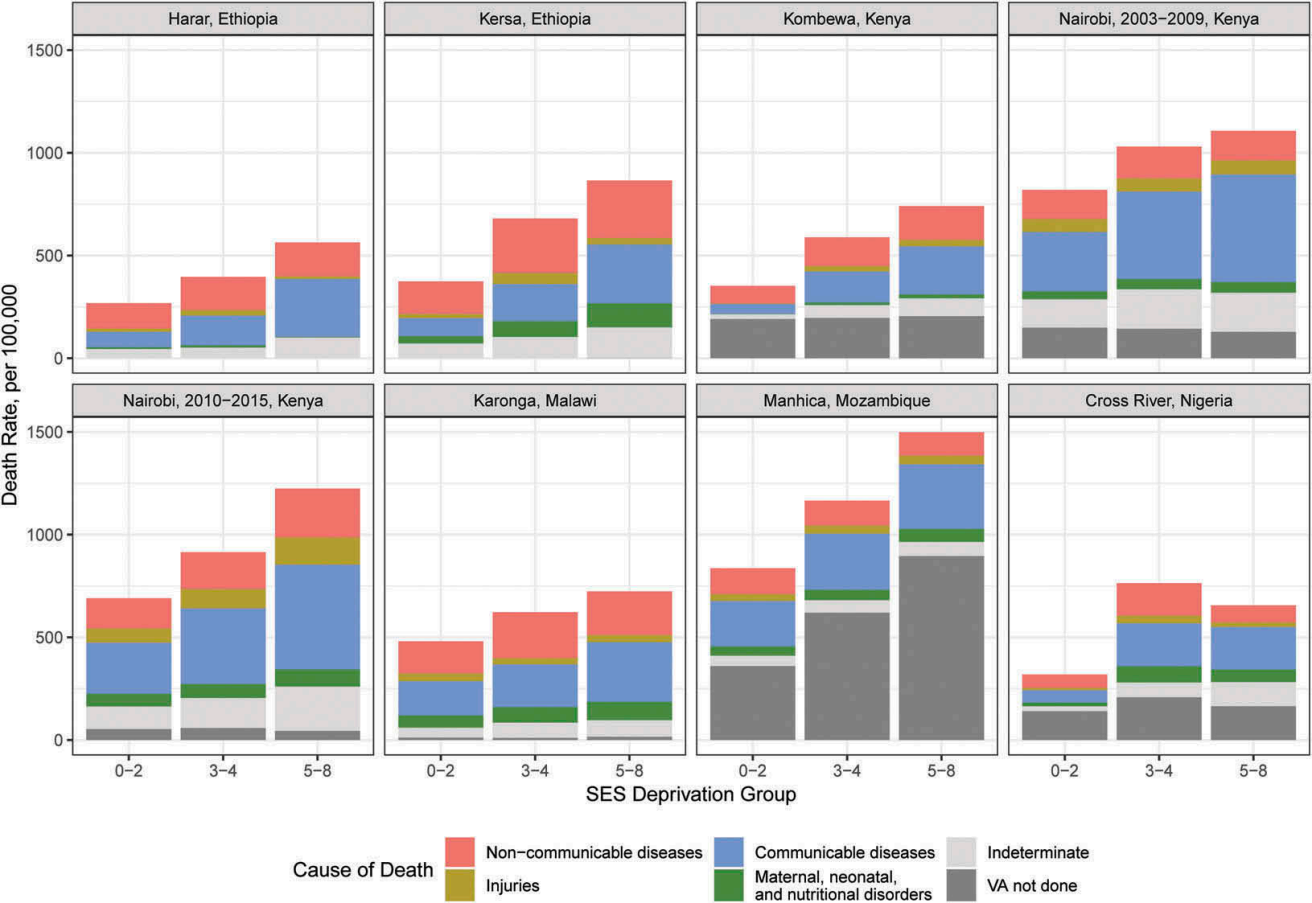
Age-sex-standardized mortality rates by HDSS site, socioeconomic group, and cause of death category.

Age-sex-standardized mortality rates varied with levels of poverty within each site (Figure 2). Crude all-age rates showed similar patterns, though the relationship between the mortality rates and deprivation groups was monotonic in Cross River HDSS (appendix Figure S5), while it was not for age-sex-standardized rates. The regression adjusting for age, year, and sex showed significantly higher rates of all-cause mortality in the 3–4 and 5–8 deprivation groups compared to the 0–2 deprivation group in every case except the small 5–8 deprivation group in Harar HDSS (appendix Table S7). The effect of greater deprivation was greatest in Cross River HDSS and lowest in Harar HDSS, with relative risks for highest compared to lowest groups of 2.06 (95% UI, 1.52–2.77) and 1.42 (0.85–2.31), respectively.

**Figure 3.**
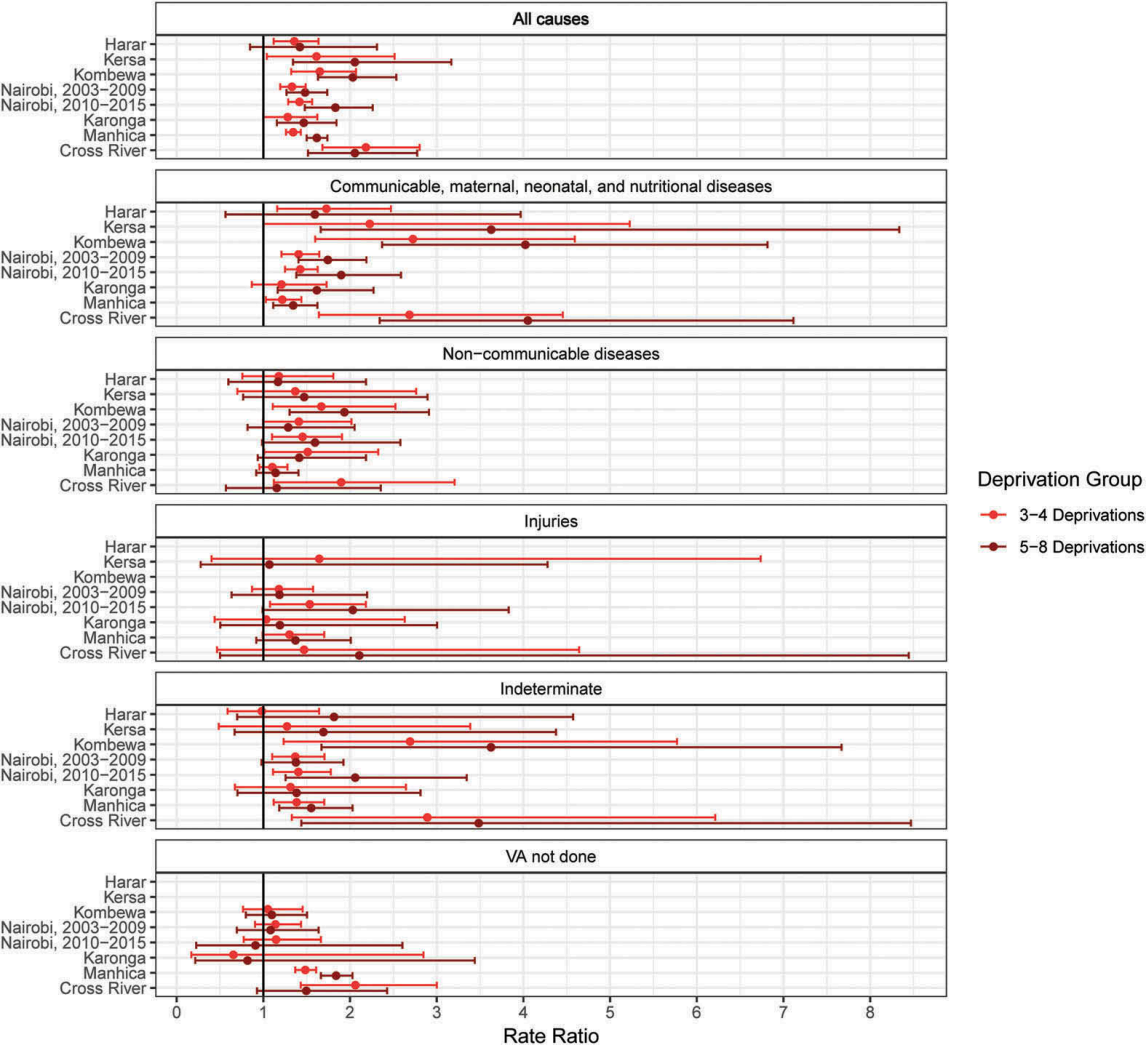
Mortality rate ratios by level of deprivation for broad causes of death: higher deprivations compared to the fewest deprivations (0–2). Note: Countries in which sites are located: Ethiopia (Harar, Kersa), Kenya (Kombewa, Nairobi), Malawi (Karonga), Mozambique (Manhiça), Nigeria (Cross River). Ratios estimated using negative binomial regressions, controlling for age, sex, and year. Kombewa and Harar HDSS deaths from injuries omitted from graph for scale (see appendix Table S7, Kombewa: 3–4 deprivation group: 8.3 [1.1–61.7], 5–8 deprivation group: 9.9 [1.3–72.5]; Harar: 3–4 deprivation group: 2.0 [1.0–4.1], 5–8 deprivation group: estimates unstable due to small number of deaths). Harar and Kersa HDSS deaths with no verbal autopsy omitted because almost every death had a verbal autopsy.

The ratio of death rates from CMNN conditions between the high-deprivation group (5–8) and the low deprivation group (0–2) ranged from 1.34 to 4.05 across sites and were significant in all sites except Harar (Figure 3). For several diseases grouped within this broad category such as HIV, malaria, and tuberculosis, higher poverty was associated with higher mortality rates, although uncertainty intervals were often wide, particularly in sites with fewer individuals or for less common COD (appendix Table S7). The point estimates of the relative rates suggested higher rates of death from NCDs among the poorer groups in each site (relative rate between 5–8 and 0–2 deprivation groups ranging 1.14–1.93). The relative rates for at least one of the 3–4 and 5–8 deprivation groups compared to the 0–2 deprivation group were significantly above one in Cross River, Karonga, Kombewa, and Nairobi (both time periods). Some of these more specific NCD categories, like acute abdomen, liver cirrhosis, and renal failure had higher death rates among poorer groups in almost every site, but relatively high uncertainty (appendix Table S7). Injury death rates had less consistent relationships with poverty, though the point estimates mostly suggested higher rates in poorer groups. Death rates from causes that were indeterminate were consistently higher among the poor, though the proportion classified as indeterminate out of deaths with completed InterVA questionnaires did not vary much by SES group (appendix Figure S6). The sites in Cross River and Manhiça were the only two with evidence of an association between poverty and rates of deaths for which no VA data were collected. Excluding deaths with no VA data and deaths for which InterVA classified the cause as indeterminate, the age-sex-standardized proportions of deaths from CMNN conditions tended to rise with poverty, while the proportion from NCDs tended to fall (appendix Figure S7). The patterns observed in the regressions using crude all-age rates were similar, though pooling across ages diminished uncertainty intervals, making more of the uncertainty bounds for NCD mortality rate ratios above one (appendix Figure S8).

As with age-sex-standardized mortality, we observed substantial differences in age-specific mortality rates across sites. Under-5 mortality rates ranged from 17 per 1,000 live births in Harar to 83 per 1,000 live births in Kersa. Age- or sex-specific death rates and proportions were more difficult to compare by SES group within sites because of small numbers (results shown in appendix). The stratified regressions suggested larger relative all-cause mortality disparities associated with SES in males in Nairobi HDSS, particularly in the second time period, though there was little evidence of sex differences in other sites. The regressions stratified by age suggested that disparities existed across all age groups. Relative all-cause mortality disparities were higher under age 15 compared to above age 40 in Harar, Karonga, and Manhiça. In Manhiça, Cross River, Karonga, Kombewa, and Nairobi, there was also evidence that the relative mortality rate disparities across SES groups were larger in ages 15 to 39 than above age 40. In Nairobi, the 15 to 39-year-old age group showed larger relative SES disparities than the under-15 age group.

**Figure 4.**
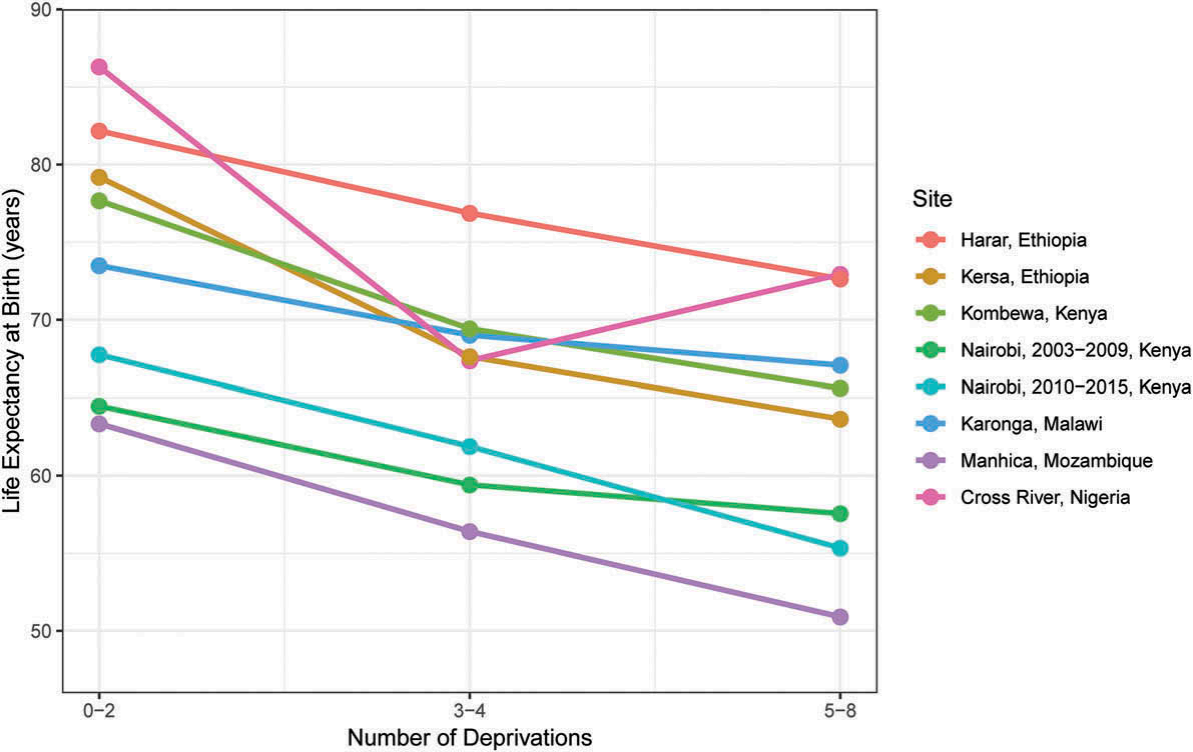
Life expectancy at birth by HDSS site and socioeconomic status.

The mortality disparities we observed by SES led to gaps in life expectancy at birth between those in the 0–2 deprivation and those in the 5–8 deprivation group ranging from 6.4 years in Karonga to 15.6 years in Kersa. The life expectancy gap between those groups was also more than 10 years in Cross River, Kombewa, and Manhiça, as well as in Nairobi in the 2010–2015 period (Figure 4).

## Discussion

Our study, using individual-level longitudinal data from seven HDSS sites in five SSA countries, showed large disparities in all-cause and cause-specific mortality rates by SES within sites. Compared to the highest socioeconomic group, mortality rates were greater in poorer groups, not only for communicable, maternal, nutritional, and neonatal conditions but also for NCDs in several HDSS sites. Relative disparities in all-cause mortality by SES were similar between sexes in most sites, though relative mortality rates between SES groups were often higher in younger age groups, particularly in younger adults (15 to 39 years of age) compared to older adults (over 40 years of age). The gap in life expectancy of over 10 years between socioeconomic groups in several of the sites in our study is on par with the difference in the average life expectancies of the USA and Kenya, yet our results are for people living within the very same communities [39].

The variation in all-cause and cause-specific deaths between sites was comparable to earlier multi-site studies of mortality in these populations, with differences in factors such as urbanicity and SES contributing to this variation [14]. For example, the populations in the Harar and Nairobi sites had fewer average deprivations on the poverty index, which may partly be related to their locations in more urban areas. However, mortality rates and composition of causes varied greatly between these sites, with lower mortality in Harar HDSS (and the highest proportion of classified deaths from NCDs) and higher mortality in Nairobi HDSS. It is possible that some of this difference in mortality is related to risks associated with the location of the Nairobi HDSS in two slum communities [27]. Local epidemiological differences, particularly relating to the HIV epidemic, are likely to have contributed to differences in mortality rates and proportions of deaths from communicable diseases between sites. Some differences between urban and rural sites were apparent; the injury mortality rates in Nairobi HDSS were much higher compared to those in the more rural sites, even after controlling for the high proportion of young adult males with particularly high injury mortality rates in the Nairobi site. Further, the gap in mortality rates between the sites in Harar and Kersa was stark (about a 5-fold difference in under-5 mortality rates), even though the two sites are separated by fewer than 100 kilometers. Large SES differences between these sites likely played an important role, but this disparity also echoes other studies showing gaps between urban and rural child mortality [40].

Results from each site showed large disparities in all-cause mortality rates between the wealthiest and poorest groups. Consistent with some previous studies, our results showed strong relationships between SES and all-cause mortality across the age spectrum [6]. Cross River, Kersa, and Kombewa had the largest relative disparities in all-cause age-sex-standardized mortality rates by SES group, though uncertainty intervals were overlapping with other sites. Local factors may lead to differences in the degree of inequality observed. Some studies have suggested that urban or semi-urban areas may show higher inequality, at least in under-5 mortality, because of greater heterogeneity in factors like piped water or electricity (while most rural households in certain areas are not likely to have these resources at all) [41]. Other factors, such as access to health facilities, may be more inequitable in rural areas. We did not see clear patterns in mortality disparities with regards to the urbanicity of sites, though our study did not explicitly examine this relationship. Previous analyses examining relationships between SES and specific causes of death in LMICs have found associations between lower SES and higher rates of death from communicable diseases, NCDs, HIV, tuberculosis, maternal mortality, and childhood illness from diarrhea and lower respiratory infections, yet these relationships do not appear consistently [8–10,20,21,23,26]. We observed associations for several of these conditions in multiple sites.

Many countries are experiencing epidemiological transitions as communicable disease burden is reduced by development and health interventions targeting these conditions, but groups within countries may face different rates of transition, with poorer populations lagging [42,43]. While our study lacks the level of detail and length of longitudinal follow-up to examine this possibility directly, we found consistently higher overall mortality rates and a larger fraction of mortality from communicable, maternal, neonatal, and nutritional diseases in the poorer groups. We also found higher rates of death from NCDs. The epidemiological transition is sometimes described as a progressive change in disease burden from communicable to a profile dominated by NCDs as populations age and become wealthier. Yet, there is also evidence that the probability of dying from an NCD is higher in LLMICs than in higher-income countries, even though the fraction of deaths from NCDs is lower [44]. Our study suggests an analogous association between poverty and NCDs at a household level – we found higher rates of NCD deaths in some poorer subpopulations. A better understanding of the drivers of existing disparities – such as low availability of NCD services, the pathways between NCDs and poverty [45,46], and various risk factors for NCDs [47,48] – are needed to inform prevention and management strategies. Our study contributes to this effort as part of the *Lancet* NCDI Poverty Commission to establish evidence about the burden of NCDIs among the poorest.

Our analyses capitalized on the strengths of HDSS data, including the granularity of household-level poverty information rather than the administrative unit comparisons often used; the verbal autopsies of deaths, which allowed us to examine COD; the routine surveillance of HDSS sites giving a denominator that allowed us to calculate cause-specific death rates and life expectancy; and the inclusion of sites from multiple countries with different settings. Despite the richness of the data from these HDSS sites, our study had several limitations. COD assigned using verbal autopsy has inherent limitations, although methods are continuously evolving [49]. However, the processes used in verbal autopsy – sourcing information from available respondents who have varying knowledge and insights about the history, symptoms, and signs leading to someone else’s death – can never be expected to provide unambiguous causes for every death in a community. In a study of this kind, a high priority is to secure methodologically comparable results across sites and over time, for which we used InterVA-4, a commonly used probabilistic model for VA questionnaires relating to World Health Organization standards [50] as used across INDEPTH sites [14,36]. The available cause classifications have been defined by the World Health Organization based on groups of ICD codes, pragmatically derived in terms of both public health importance and feasibility using verbal autopsy. However, it is easier to classify some types of deaths, such as injuries, using verbal autopsy, compared to deaths associated with more general symptoms like abdominal pain [49]. InterVA-4, in modeling likely COD on a case-by-case basis, does not have any input characterizing the socioeconomic status of the deceased. HDSS data provide useful subnational estimates, but their representativeness of wider populations may vary. Nonetheless, a co-validation study between INDEPTH HDSS mortality data and Global Burden of Disease mortality estimates showed strong similarities across a range of countries, suggesting that HDSS data may be more generalizable than is sometimes assumed [51]. The consistency of our findings across multiple sites from five SSA countries suggests that socioeconomic disparities in mortality rates are widespread across different settings and that the patterns of COD also vary by SES. HDSS-specific data were sufficiently large, in most instances, to detect differences in mortality rates by broad cause groups, but stochastic variation may explain some of the observed differences in smaller age, sex, and SES subgroup analyses. For instance, it is unclear whether the age-sex-standardized mortality rate in Cross River is higher in the 3–4 deprivation group than the 5–8 deprivation group because of unstable rates, particularly because the crude mortality rates do not show this pattern.

## Conclusion

Our findings highlight that socioeconomic disparities within relatively small communities can be quite large in SSA countries. Using available data from smaller area-based units, such as HDSS sites, provides locally relevant insights into health inequality, alignment with administrative units of government, and greater ability to reach communities [52]. Understanding the impact of inequalities within these communities is important to ensure that these types of disparities are addressed when designing interventions for prevention and management of disease.

## Paper context

Lower socioeconomic status (SES) is well known to be associated with higher risk of mortality in low- and lower-middle-income countries. However, studies have found mixed associations between SES and specific causes of death. Our study finds higher rates of death from communicable diseases, and in some cases non-communicable diseases, in poorer groups, leading to large life expectancy disparities even within communities. Understanding disparities in mortality from diseases can help target prevention and treatment efforts.

## References

[1] Feinstein JS. The relationship between socioeconomic status and health: a review of the literature. Milbank Q. 1993;71:279–12. 8510603

[2] Auerbach JA, Krimgold BK, editors. Income, socioeconomic status, and health: exploring the relationships. Washington, D.C: Academy for Health Services Research and Health Policy; 2001.

[3] Adler Nancy E, Ostrove Joan M Socioeconomic status and health: what we know and what we don’t. Ann N Y Acad Sci. 2006;896:3–15. 10.1111/j.1749-6632.1999.tb08101.x 10681884

[4] Braveman P, Tarimo E Social inequalities in health within countries: not only an issue for affluent nations. Soc Sci Med . 2002;54:1621–1635. 12113445 10.1016/s0277-9536(01)00331-8

[5] Houweling TAJ, Kunst AE Socio-economic inequalities in childhood mortality in low- and middle-income countries: a review of the international evidence. Br Med Bull. 2010;93:7–26. 20007188 10.1093/bmb/ldp048

[6] Po JYT, Subramanian SV. Mortality Burden and Socioeconomic Status in India. PLoS ONE 2011;6(2): e16844. doi: 10.1371/journal.pone.0016844. PMC3036714 21347373

[7] McKinnon B, Harper S, Kaufman JS, et al Socioeconomic inequality in neonatal mortality in countries of low and middle income: a multicountry analysis. Lancet Glob Health. 2014;2:e165–e173. 25102849 10.1016/S2214-109X(14)70008-7

[8] Hanifi SMA, Mahmood SS, Bhuiya A. Cause-specific mortality and socioeconomic status in Chakaria, Bangladesh, Global Health Action, 7:1. Chilet-Rosell E Gender bias in clinical research, pharmaceutical marketing, and the prescription of drugs. Glob Health Action. 2014;7:25473.doi: 10.3402/gha.v7.25473 25377331 PMC4220129

[9] Rossier C, Soura AB, Duthé G, et al Non-communicable disease mortality and risk factors in formal and informal neighborhoods, ouagadougou, burkina faso: evidence from a health and demographic surveillance system. PLoS One. 2014;9:e113780. 25493649 10.1371/journal.pone.0113780 PMC4262303

[10] Kabudula CW, Houle B, Collinson MA, et al Socioeconomic differences in mortality in the antiretroviral therapy era in agincourt, rural South Africa, 2001–13: a population surveillance analysis. Lancet Glob Health. 2017;5:e924–e935. 28807190 10.1016/S2214-109X(17)30297-8 PMC5559644

[11] United Nations Transforming our world: the 2030 agenda for sustainable development. United Nations; 2015 10.

[12] Hosseinpoor AR, Bergen N, Magar V Monitoring inequality: an emerging priority for health post-2015. Bull World Health Organ. 2015;93:591–591A. 26478619 10.2471/BLT.15.162081 PMC4581651

[13] INDEPTH Network Measuring health equity in small areas: findings from demographic surveillance systems. Burlington, VT: Ashgate Pub Co; 2005.

[14] Streatfield PK, Khan WA, Bhuiya A, et al Cause-specific mortality in Africa and Asia: evidence from INDEPTH health and demographic surveillance system sites. Glob Health Action. 2014;7:25362. 25377324 10.3402/gha.v7.25362 PMC4220126

[15] Sankoh O, Byass P The INDEPTH network: filling vital gaps in global epidemiology. Int J Epidemiol. 2012;41:579–588. 22798690 10.1093/ije/dys081 PMC3396316

[16] Naghavi M, Abajobir AA, Abbafati C, et al Global, regional, and national age-sex specific mortality for 264 causes of death, 1980–2016: a systematic analysis for the global burden of disease study 2016. Lancet. 2017;390:1151–1210. 28919116 10.1016/S0140-6736(17)32152-9 PMC5605883

[17] Santosa A, Byass P Diverse empirical evidence on epidemiological transition in low- and middle-income countries: population-based findings from INDEPTH network data. PLoS One. 2016;11:e0155753. 27187781 10.1371/journal.pone.0155753 PMC4871496

[18] Bawah A, Houle B, Alam N, et al The evolving demographic and health transition in four low- and middle-income countries: evidence from four Sites in the INDEPTH network of longitudinal health and demographic surveillance systems. PLoS One. 2016;11:e0157281. 27304429 10.1371/journal.pone.0157281 PMC4909223

[19] Kabudula CW, Houle B, Collinson MA, et al Progression of the epidemiological transition in a rural South African setting: findings from population surveillance in Agincourt, 1993–2013. BMC Public Health. 2017;17:424. 28486934 10.1186/s12889-017-4312-x PMC5424387

[20] Arifeen S, Black RE, Antelman G, et al Exclusive breastfeeding reduces acute respiratory infection and diarrhea deaths among infants in Dhaka slums. Pediatrics. 2001;108:E67. 11581475 10.1542/peds.108.4.e67

[21] Bell JS, Ouédraogo M, Ganaba R, et al The epidemiology of pregnancy outcomes in rural Burkina Faso. Trop Med Int Health TM IH. 2008;13:31–43. 10.1111/j.1365-3156.2008.02085.x 18578810

[22] Nabukalu D, Klipstein-Grobusch K, Herbst K, et al Mortality in women of reproductive age in rural South Africa. Glob Health Action. 2013;6:22834. 24360403 10.3402/gha.v6i0.22834 PMC3869952

[23] Mee P, Collinson MA, Madhavan S, et al Determinants of the risk of dying of HIV/AIDS in a rural South African community over the period of the decentralised roll-out of antiretroviral therapy: a longitudinal study. Glob Health Action. 2014;7:24826. 25416322 10.3402/gha.v7.24826 PMC4245451

[24] Amek NO, Van Eijk A, Lindblade KA, et al Infant and child mortality in relation to malaria transmission in KEMRI/CDC HDSS, Western Kenya: validation of verbal autopsy. Malar J. 2018;17:37. 29347942 10.1186/s12936-018-2184-x PMC5774157

[25] Abera SF, Gebru AA, Biesalski HK, et al Social determinants of adult mortality from non-communicable diseases in northern Ethiopia, 2009–2015: evidence from health and demographic surveillance site. PLoS One. 2017;12:e0188968. 29236741 10.1371/journal.pone.0188968 PMC5728486

[26] Jahn A, Floyd S, McGrath N, et al Child mortality in rural Malawi: HIV closes the survival gap between the socio-economic strata. PLoS One. 2010;5:e11320. 20596521 10.1371/journal.pone.0011320 PMC2893132

[27] Beguy D, Elung’ata P, Mberu B, et al Health & demographic surveillance system profile: the nairobi urban health and demographic surveillance system (NUHDSS). Int J Epidemiol. 2015;44:462–471. 25596586 10.1093/ije/dyu251

[28] Assefa N, Oljira L, Baraki N, et al HDSS profile: the kersa health and demographic surveillance system. Int J Epidemiol. 2016;45:94–101. 26510420 10.1093/ije/dyv284 PMC4795560

[29] Crampin AC, Dube A, Mboma S, et al Profile: the karonga health and demographic surveillance system. Int J Epidemiol. 2012;41:676–685. 22729235 10.1093/ije/dys088 PMC3396313

[30] Sacoor C, Nhacolo A, Nhalungo D, et al Profile: manhiça health research centre (Manhiça HDSS). Int J Epidemiol. 2013;42:1309–1318. 24159076 10.1093/ije/dyt148

[31] Sifuna P, Oyugi M, Ogutu B, et al Health & demographic surveillance system profile: the kombewa health and demographic surveillance system (Kombewa HDSS). Int J Epidemiol. 2014;43:1097–1104. 25009309 10.1093/ije/dyu139 PMC4258789

[32] Alkire S, Santos ME Acute multidimensional poverty: a new index for developing countries [Internet]. University of Oxford, Report No.: 38. 2010 Available from: http://www.ophi.org.uk/acute-multidimensional-poverty-a-new-index-for-developing-countries/

[33] Bukhman G, Mocumbi AO, Horton R Reframing NCDs and injuries for the poorest billion: a lancet commission. Lancet. 2015;386:1221–1222. 26403914 10.1016/S0140-6736(15)00278-0

[34] Moons KGM, Donders RART, Stijnen T, et al Using the outcome for imputation of missing predictor values was preferred. J Clin Epidemiol. 2006;59:1092–1101. 16980150 10.1016/j.jclinepi.2006.01.009

[35] Honaker J, King G, Blackwell M Amelia II: a program for missing data. J Stat Softw. 2011;45:1–47.

[36] Byass P, Chandramohan D, Clark SJ, et al Strengthening standardised interpretation of verbal autopsy data: the new InterVA-4 tool. Glob Health Action. 2012;5:19281. 10.3402/gha.v5i0.19281 PMC3433652 22944365

[37] Sankoh O, Sharrow D, Herbst K, et al The INDEPTH standard population for low- and middle-income countries, 2013. Glob Health Action. 2014;7:23286. 24679543 10.3402/gha.v7.23286 PMC3969509

[38] Preston SH, Heuveline P, Guillot M. Demography: Measuring and modeling population processes. Blackwell Publishers; 2001.

[39] Hay SI, Abajobir AA, Abate KH, et al Global, regional, and national disability-adjusted life-years (DALYs) for 333 diseases and injuries and healthy life expectancy (HALE) for 195 countries and territories, 1990–2016: a systematic analysis for the Global Burden of Disease Study 2016. Lancet. 2017;390:1260–1344. 28919118 10.1016/S0140-6736(17)32130-X PMC5605707

[40] Wang L Determinants of child mortality in LDCs: empirical findings from demographic and health surveys. Health Policy. 2003;65:277–299. 12941495 10.1016/s0168-8510(03)00039-3

[41] Schoeps A, Souares A, Niamba L, et al Childhood mortality and its association with household wealth in rural and semi-urban Burkina Faso. Trans R Soc Trop Med Hyg. 2014;108:639–647. 25129891 10.1093/trstmh/tru124

[42] Frenk J, Bobadilla JL, Sepuúlveda J, et al Health transition in middle-income countries: new challenges for health care. Health Policy Plan. 1989;4:29–39.

[43] Agyei-Mensah S, de-Graft Aikins A Epidemiological transition and the double burden of disease in Accra, Ghana. J Urban Health Bull N Y Acad Med. 2010;87:879–897. 10.1007/s11524-010-9492-y PMC2937133 20803094

[44] Murray CJ, Lopez AD Mortality by cause for eight regions of the world: global burden of disease study. Lancet Lond Engl. 1997;349:1269–1276. 10.1016/S0140-6736(96)07493-4 9142060

[45] Jan S, Laba T-L, Essue BM, et al Action to address the household economic burden of non-communicable diseases. Lancet. 2018;391:2047–2058. 29627161 10.1016/S0140-6736(18)30323-4

[46] Niessen LW, Mohan D, Akuoku JK, et al Tackling socioeconomic inequalities and non-communicable diseases in low-income and middle-income countries under the sustainable development agenda. Lancet. 2018;391:2036–2046. 29627160 10.1016/S0140-6736(18)30482-3

[47] Allen L, Williams J, Townsend N, et al Socioeconomic status and non-communicable disease behavioural risk factors in low-income and lower-middle-income countries: a systematic review. Lancet Glob Health. 2017;5:e277–e289. 28193397 10.1016/S2214-109X(17)30058-X PMC5673683

[48] Ogoina D, Onyemelukwe GC The role of infections in the emergence of non-communicable diseases (NCDs): compelling needs for novel strategies in the developing world. J Infect Public Health. 2009;2:14–29. 20701857 10.1016/j.jiph.2009.02.001 PMC7102799

[49] Murray CJ, Lozano R, Flaxman AD, et al Using verbal autopsy to measure causes of death: the comparative performance of existing methods. BMC Med. 2014;12:5. 24405531 10.1186/1741-7015-12-5 PMC3891983

[50] Nichols EK, Byass P, Chandramohan D, Clark SJ, Flaxman AD, Jakob R, et al. The WHO 2016 verbal autopsy instrument: An international standard suitable for automated analysis by InterVA, InSilicoVA, and Tariff 2.0. PLOS Med. 2018;15: e1002486 doi:10.1371/journal.pmed.1002486 PMC5761828 29320495

[51] Byass P. Cause-specific mortality findings from the Global Burden of Disease project and the INDEPTH Network. Lancet Glob Health. 2016;4: e785–e786. 10.1016/S2214-109X(16)30203-0 27765285

[52] Hosseinpoor AR, Bergen N. Area-based units of analysis for strengthening health inequality monitoring. Bull World Health Organ. 2016;94: 856–858. 10.2471/BLT.15.165266 PMC5096344 27821889

