# Supplementary material for: A comparison of all-cause and cause-specific mortality by household socioeconomic status across seven INDEPTH network health and demographic surveillance systems in sub-Saharan Africa
Source: Glob Health Action. 2019 May 16;12(1):1608013. doi: 10.1080/16549716.2019.1608013 (PMC6534200; doi:10.1080/16549716.2019.1608013)
Supplement: Supplemental Material [file ZGHA_A_1608013_SM5527.pdf]

Appendix

METHODS

Table S1: SES Indicators

Table S2: Adaptations to PI indicators for specific sites

Table S3: Cause of death classification categories

Multiple imputation methods

Table S4: Characterizing missingness of socioeconomic data by site, in sites with longitudinal SES data

Table S5: Characterizing missingness of socioeconomic data by site, in Harar and Kersa

Table S6: Characterizing missingness of socioeconomic data by site, in Kombewa and Cross River

Life expectancy calculations

RESULTS

Population age and sex structure (Appendix Figures S1-S3)

Figure S4: All-age proportions of broad causes of death among deaths among (a) all deaths and (b) deaths assigned causes by InterVA

Figure S5: All-age mortality rates by site, socioeconomic group, and cause of death category

Figure S6: Age-sex-standardized death proportions by site, socioeconomic group, and cause of death category, excluding deaths for which VA not conducted

Figure S7: Age-sex-standardized death proportions by site, socioeconomic group, and cause of death category, excluding deaths for which VA not conducted or InterVA assigned cause of death is indeterminate

Table S7: Rate ratios for cause-specific death rates between SES groups, controlling for year, sex, and age (with 95% uncertainty intervals), 0-2 deprivations as reference group

Figure S8: All-age mortality rate ratios by level of deprivation for broad causes of death: higher deprivations compared to the fewest deprivations (0-2)

Figure S9: All-cause mortality rate ratios by level of deprivation: higher deprivations compared to the least deprivations (0-2), for males and females

Figure S10: All-cause mortality rate ratios by level of deprivation: higher deprivations compared to the least deprivations (0-2), for age groups under-15, 15 to 39, and 40 and older

SENSITIVITY ANALYSES

Figure S11: Age-sex-standardized mortality rates by socioeconomic group and cause of death category, comparing SES assumptions

Table S1: SES Indicators

Indicator	Deprivation definition
School Attendance	Any school-aged child in household is not attending school up to class 8
Education Completed	No household member has completed five years of schooling
Electricity	Household has no electricity
Sanitation	Household's sanitation facility not improved or shared with other households
Water	Household does not have access to improved drinking water source, or source is more than 30-minute walk, round trip
Flooring	Household has dirt, sand, or dung floor
Cooking Fuel	Household cooks with dung, wood, or charcoal
Assets	Household does not own a car or truck AND does not own more than one of: radio, TV, telephone, bike, motorbike, or refrigerator
Adapted from Alkire and Santos (2010) [1]	

Table S2: Adaptations to PI indicators for specific sites

Site	Adaptations
Harar	No data on car, car criterion excluded from criteria in asset indicator, but asset indicator still included in full 8 indicators.
Kersa	Motorbike variable unavailable, motorbike criterion excluded from criteria in asset indicator, but asset indicator still included in full 8 indicators.
Kombewa	-
Nairobi	Electricity variable missing, but replaced by assuming household with any one of the following had electricity: television, refrigerator, fan, cooking with electricity, or electrical light source. Time to get water not collected, but assumed < 30 minutes given urban setting.
Karonga	-
Manhiça	No education variables, PI included 6 indicators instead of 8. Freezer used in place of refrigerator.
Cross River	-

Table S3: Cause of death classification categories

InterVA Cause	Level 3 Cause Category	Level 2 Cause Category	Level 1 Cause Category
**01.01 Sepsis (non-obstetric)**	Other infectious diseases	Communicable diseases	Communicable, maternal, neonatal, and nutritional diseases
**01.02 Acute resp infect incl pneumonia**	Lower respiratory infection	Communicable diseases	Communicable, maternal, neonatal, and nutritional diseases
**01.03 HIV/AIDS related death**	HIV/AIDS	Communicable diseases	Communicable, maternal, neonatal, and nutritional diseases
**01.04 Diarrhoeal diseases**	Diarrheal disease	Communicable diseases	Communicable, maternal, neonatal, and nutritional diseases
**01.05 Malaria**	Malaria	Communicable diseases	Communicable, maternal, neonatal, and nutritional diseases
**01.06 Measles**	Other infectious diseases	Communicable diseases	Communicable, maternal, neonatal, and nutritional diseases
**01.07 Meningitis and encephalitis**	Other infectious diseases	Communicable diseases	Communicable, maternal, neonatal, and nutritional diseases
**01.08 & 10.05 Tetanus**	Other infectious diseases	Communicable diseases	Communicable, maternal, neonatal, and nutritional diseases
**01.09 Pulmonary tuberculosis**	Tuberculosis	Communicable diseases	Communicable, maternal, neonatal, and nutritional diseases
**01.10 Pertussis**	Other infectious diseases	Communicable diseases	Communicable, maternal, neonatal, and nutritional diseases
**01.11 Haemorrhagic fever**	Other infectious diseases	Communicable diseases	Communicable, maternal, neonatal, and nutritional diseases
**01.99 Other and unspecified infect dis**	Other infectious diseases	Communicable diseases	Communicable, maternal, neonatal, and nutritional diseases
**02.01 Oral neoplasms**	Other neoplasms	Non-communicable diseases	Non-communicable diseases
**02.02 Digestive neoplasms**	Other neoplasms	Non-communicable diseases	Non-communicable diseases
**02.03 Respiratory neoplasms**	Respiratory neoplasms	Non-communicable diseases	Non-communicable diseases
**02.04 Breast neoplasms**	Other neoplasms	Non-communicable diseases	Non-communicable diseases
**02.05 & 02.06 Reproductive neoplasms MF**	Reproductive neoplasms	Non-communicable diseases	Non-communicable diseases
**02.99 Other and unspecified neoplasms**	Other neoplasms	Non-communicable diseases	Non-communicable diseases
**03.01 Severe anaemia**	Anemia and malnutrition	Nutritional disorders	Communicable, maternal, neonatal, and nutritional diseases
**03.02 Severe malnutrition**	Anemia and malnutrition	Nutritional disorders	Communicable, maternal, neonatal, and nutritional diseases
**03.03 Diabetes mellitus**	Diabetes	Non-communicable diseases	Non-communicable diseases
**04.01 Acute cardiac disease**	Cardiovascular diseases	Non-communicable diseases	Non-communicable diseases
**04.03 Sickle cell with crisis**	Other non-communicable diseases	Non-communicable diseases	Non-communicable diseases
**04.02 Stroke**	Cardiovascular diseases	Non-communicable diseases	Non-communicable diseases
**04.99 Other and unspecified cardiac dis**	Cardiovascular diseases	Non-communicable diseases	Non-communicable diseases
**05.01 Chronic obstructive pulmonary dis**	Chronic respiratory diseases	Non-communicable diseases	Non-communicable diseases
**05.02 Asthma**	Chronic respiratory diseases	Non-communicable diseases	Non-communicable diseases
**06.01 Acute abdomen**	Acute abdomen, liver cirrhosis, renal failure	Non-communicable diseases	Non-communicable diseases

**06.02 Liver cirrhosis**	Acute abdomen, liver cirrhosis, renal failure	Non-communicable diseases	Non-communicable diseases
**07.01 Renal failure**	Acute abdomen, liver cirrhosis, renal failure	Non-communicable diseases	Non-communicable diseases
**08.01 Epilepsy**	Epilepsy	Non-communicable diseases	Non-communicable diseases
**98 Other and unspecified NCD**	Other non-communicable diseases	Non-communicable diseases	Non-communicable diseases
**10.06 Congenital malformation**	Other non-communicable diseases	Non-communicable diseases	Non-communicable diseases
**10.01 Prematurity**	Neonatal disorders	Neonatal disorders	Communicable, maternal, neonatal, and nutritional diseases
**10.02 Birth asphyxia**	Neonatal disorders	Neonatal disorders	Communicable, maternal, neonatal, and nutritional diseases
**10.03 Neonatal pneumonia**	Neonatal disorders	Neonatal disorders	Communicable, maternal, neonatal, and nutritional diseases
**10.04 Neonatal sepsis**	Neonatal disorders	Neonatal disorders	Communicable, maternal, neonatal, and nutritional diseases
**10.99 Other and unspecified neonatal CoD**	Neonatal disorders	Neonatal disorders	Communicable, maternal, neonatal, and nutritional diseases
**11.01 Fresh stillbirth**	Not CoD	Not CoD	Not CoD
**11.02 Macerated stillbirth**	Not CoD	Not CoD	Not CoD
**12.01 Road traffic accident**	Transport injuries	Injuries	Injuries
**12.02 Other transport accident**	Transport injuries	Injuries	Injuries
**12.03 Accid fall**	Unintentional injuries	Injuries	Injuries
**12.04 Accid drowning and submersion**	Unintentional injuries	Injuries	Injuries
**12.05 Accid expos to smoke fire & flame**	Unintentional injuries	Injuries	Injuries
**12.06 Contact with venomous plant/animal**	Unintentional injuries	Injuries	Injuries
**12.10 Exposure to force of nature**	Other injuries	Injuries	Injuries
**12.07 Accid poisoning & noxious subs**	Unintentional injuries	Injuries	Injuries
**12.08 Intentional self-harm**	Intentional injuries	Injuries	Injuries
**12.09 Assault**	Intentional injuries	Injuries	Injuries
**12.99 Other and unspecified external CoD**	Other injuries	Injuries	Injuries
**09.01 Ectopic pregnancy**	Maternal disorders	Maternal disorders	Communicable, maternal, neonatal, and nutritional diseases
**09.02 Abortion-related death**	Maternal disorders	Maternal disorders	Communicable, maternal, neonatal, and nutritional diseases
**09.03 Pregnancy-induced hypertension**	Maternal disorders	Maternal disorders	Communicable, maternal, neonatal, and nutritional diseases
**09.04 Obstetric haemorrhage**	Maternal disorders	Maternal disorders	Communicable, maternal, neonatal, and nutritional diseases
**09.05 Obstructed labour**	Maternal disorders	Maternal disorders	Communicable, maternal, neonatal, and nutritional diseases
**09.06 Pregnancy-related sepsis**	Maternal disorders	Maternal disorders	Communicable, maternal, neonatal, and nutritional diseases
**09.07 Anaemia of pregnancy**	Maternal disorders	Maternal disorders	Communicable, maternal, neonatal, and nutritional diseases

**09.08 Ruptured uterus**	Maternal disorders	Maternal disorders	Communicable, maternal, neonatal, and nutritional diseases
**09.99 Other and unspecified maternal CoD**	Maternal disorders	Maternal disorders	Communicable, maternal, neonatal, and nutritional diseases
**99 Indeterminate**	Indeterminate	Indeterminate	Indeterminate
**Va not done**	VA not done	VA not done	VA not done

Not CoD = Not Cause of Death (stillbirths not included as deaths in our analysis)

Multiple imputation methods
We utilized the available data to impute SES indicators in cases of missingness. We were able to use longitudinal SES data in the Nairobi, Karonga, and Manhiça site but SES data from a single year in the other sites. Those different processes are explained separately below. Relatively small proportions of person-time and deaths had no corresponding SES data. In each case, we incorporated uncertainty by creating 20 imputed datasets, carrying forward uncertainty into our model results by conducting analysis on each of the 20 imputed datasets. Every imputation used the Amelia II software package in R [2].

Nairobi: In Nairobi, we utilized the full time series of data during imputation, rather than breaking into two periods as in our results presentation. This allowed for better use of information during imputation. The dataset was formatted to be one observation per household per calendar year. We sought to impute missing values for each of the 7 non-asset indicators as well as each of the individual assets that comprise the asset indicator. In our model, we also included terms for the household, the neighborhood (Korogocho or Viwandani) of the house, a linear time effect, one year of lags and leads of all of the indicators we sought to impute, the number of residents leaving the house in that year, the number of residents moving in to the house that year, the number of births in the household that year, the number of deaths in the household that year, the person-time lived in the household that year, the number of residents of various age and gender groups (male under-15, female under-15, male 15-49, female 15-49, male 50-69, female 50-69, male 70+, female 70+), number of years the household existed from the start of the study period, an indicator for households that existed in the first year of the study period, and an interaction of the number of years the household existed since the start of the study period and the indicator for those existing in the first year of the study period. Overall, only 2.8% person-time and 7% of deaths in the Nairobi site were in households without a single SES indicator collected. In Nairobi, these indicators were part of regular data collection. In most cases, a given household year would either have all variables available or none; partial collection was rare.

Karonga: The dataset was formatted to be one observation per household per calendar year. We sought to impute missing values for each of the 8 indicators on our poverty index. In our model, we also included terms for the household, a linear time effect, one year of lags and leads of all of the indicators we sought to impute, the number of residents leaving the house in that year, the number of residents moving in to the house that year, the number of births in the household that year, the number of deaths in the household that year, the person-time lived in the household that year, the number of residents of various age and gender groups (male under-15, female under-15, male 15-49, female 15-49, male 50-69, female 50-69, male 70+, female 70+), number of years the household existed from the start of the study period, an indicator for households that existed in the first year of the study period, and an interaction of the number of years the household existed since the start of the study period and the indicator for those existing in the first year of the study period. Overall, almost all households (97.7%) had at least one SES indicator collected during their existence. Unlike some other sites, SES data were primarily collected through specific studies, so the times at which different indicators were collected varied. This led to a large increase from the percent of households with no SES indicators collected (2.3%) to the percent of households with 4 or more SES indicators never collected (33.0%) compared to other sites.

Manhiça: The dataset was formatted to be one observation per household per calendar year. We sought to impute missing values for each of the 5 non-asset indicators as well as each of the individual assets that comprise the asset indicator. In our model, we also included terms for the household, neighborhood averages prevalence of deprivation in each of the poverty indicators, a linear time effect, one year of lags and leads of all of the indicators we sought to impute, the number of residents leaving the house in that year, the number of residents moving in to the house that year, the number of births in the household that year, the number of deaths in the household that year, the person-time lived in the household that year, the number of residents of various age and gender groups (male under-15, female under-15, male 15-49, female 15-49, male 50-69, female 50-69, male 70+, female 70+), number of years the household existed from the start of the study period, an indicator for households that existed in the first year of the study period, and an interaction of the number of years the household existed since the start of the study period and the indicator for those existing in the first year of the study period. The pattern of missingness in Manhiça is a mix between the patterns of Nairobi and Karonga. Indicators are mostly collected together in the same years; however, there are a number of households with fuel source, sanitation, electricity, and flooring collected in the same year, but

not the other indicators. Overall, the percent of person-time in households with no SES indicators collected was low (2.8%), but the percent of households with half or more SES indicators never collected (21.9%) was between those of Nairobi and Karonga.

Table S4: Characterizing missingness of socioeconomic data by site, in sites with longitudinal SES data

	Nairobi	Karonga	Manhiça*
Percent of households with no SES indicators collected	11.8%	2.3%	12.0%
Percent of household person-time with no SES indicators collected	2.8%	0.2%	2.8%
Percent of deaths with no SES indicators	7.0%	0.1%	2.9%
Percent of households with no SES data that only existed in 2 or fewer years (how often are households missing data mainly because they're not around for long)	85.8%	93.6%	58.7%
Percent of households with 3+ years of existence and SES data only in 1 year	8.6%	<0.1%	1.7%
Percent of households with half or more SES indicators never collected	12.6%	33.0%	21.9%

*Manhiça missingness reported out of 6 indicators

Harar and Kersa: Living standards data were available for one year in each household—either the beginning of the study period or the year that the household was created if it was created after the beginning of the study period. Education data were available at multiple time points, including 2013 and 2016, the beginning and end years of the study period. First, we imputed education information using several assumptions. We needed information about whether individuals were attending school (if they were of the age range expected to attend up to grade 8). We also needed information on the highest educational attainment. If the person was currently attending school, we made the assumption that they had attended school in previous years (provided data were not available on those years). If they were attending school, we utilized their attainment at the time of the survey and imputed back in time by subtracting a year of attainment for each year back in time. If attainment recorded in previous years was greater than what was imputed, then we used the attainment from the previous years. For adults over age 25, we assumed educational attainment did not change over the time period (a fair assumption in relation to the indicator regarding members of the household attaining 5 years of education). We created the education indicators at the household level. We marked the variables missing if there was insufficient information about the individuals in the household to determine whether that household should be categorized as deprived in the given category. We also created variables for whether there were school-aged children in the household, the total children in the household, and the number of those children missing information in order to inform the imputation model.

We combined the education variables with the living standards dataset. The dataset was formatted to be one observation per household, including data from the beginning year (2013) or the first year of the household's inclusion in the surveillance site. To add information for the imputation model to use about neighboring households, we calculated the proportion of neighboring households with deprivations in each indicator. We sought to impute missing values for each of the 7 non-asset indicators as well as each of the individual assets that comprise the asset indicator. In our model, we also included terms for the household, average proportion of neighboring households deprived in each poverty indicator, reported monthly income, the number of residents leaving the house in that year, the number of residents moving in to the house that year, the number of births in the household that year, the number of deaths in the household that year, the person-time lived in the household that year, the number of residents of various age and gender groups (male under-15, female under-15, male 15-49, female 15-49, male 50-69, female 50-69, male 70+, female 70+), number of years the household existed from the start of the study period, an indicator for households that existed in the first year of the study period, and an interaction of the number of years the household existed since the start of the study period and the indicator for those existing in the first year of the study period. The non-education indicators were missing in about 11% of households in Harar and 4% of households in Kersa, while the education indicators were missing in 8.8% and 17.7% of households in Harar and 37.5% and 24.7% of households in Kersa in after the initial assumptions.

Table S5: Characterizing missingness of socioeconomic data by site, in Harar and Kersa

	Harar	Kersa
Percent of households with no SES indicators collected in initial year	1.8%	1.0%
Percent of households with half or more SES indicators not collected in baseline year	11.1%	4.2%

Kombewa: In Kombewa, we assumed the SES survey data in the initial year to be representative for the corresponding age and sex groups in the sites to assign person-years to SES groups for denominators. For deaths, we merged SES survey data with the death data by household of residence. We found that 4.0% of deaths had missing SES data associated. We imputed the total number of deprivations (ordinal variable, 0-8) for these deaths using age group, sex, and year. Most of the deaths without SES data collected also did not have verbal autopsy data collected (93%), suggesting these households may have been more difficult to reach or were less likely to participate in surveys. These deaths without SES data or verbal autopsy collected constituted 12.5% of deaths in which verbal autopsy data were not collected.

Cross River: In Cross River, we assumed the SES survey data in the initial year to be representative for the corresponding age and sex groups in the sites to assign person-years to SES groups for denominators. For deaths, we merged SES survey data with the death data by household of residence. We found that 1.7% of deaths had missing associated SES data. We imputed the total number of deprivations (ordinal variable, 0-8) for these deaths using age group, sex, and year.

Table S6: Characterizing missingness of socioeconomic data by site, in Kombewa and Cross River

	Kombewa	Cross River
Percent of deaths with missing SES data for household	4.0%	1.7%

Life expectancy calculations

We had mortality rates for under-1 year, 1-4 years, and then 5-year age groups to age 85 and over for each site, stratified by three SES groups: 0-2 deprivations, 3-4 deprivations, and 5-8 deprivations on our poverty index. We also calculated ${}_na_x$ (average age of death within each age group) from the datasets [3]. We then utilized ${}_nm_x$ and ${}_na_x$ to generate full lifetables for each group and show life expectancy at birth.

Figure S1:

Total site populations by

age, sex, and number of deprivations

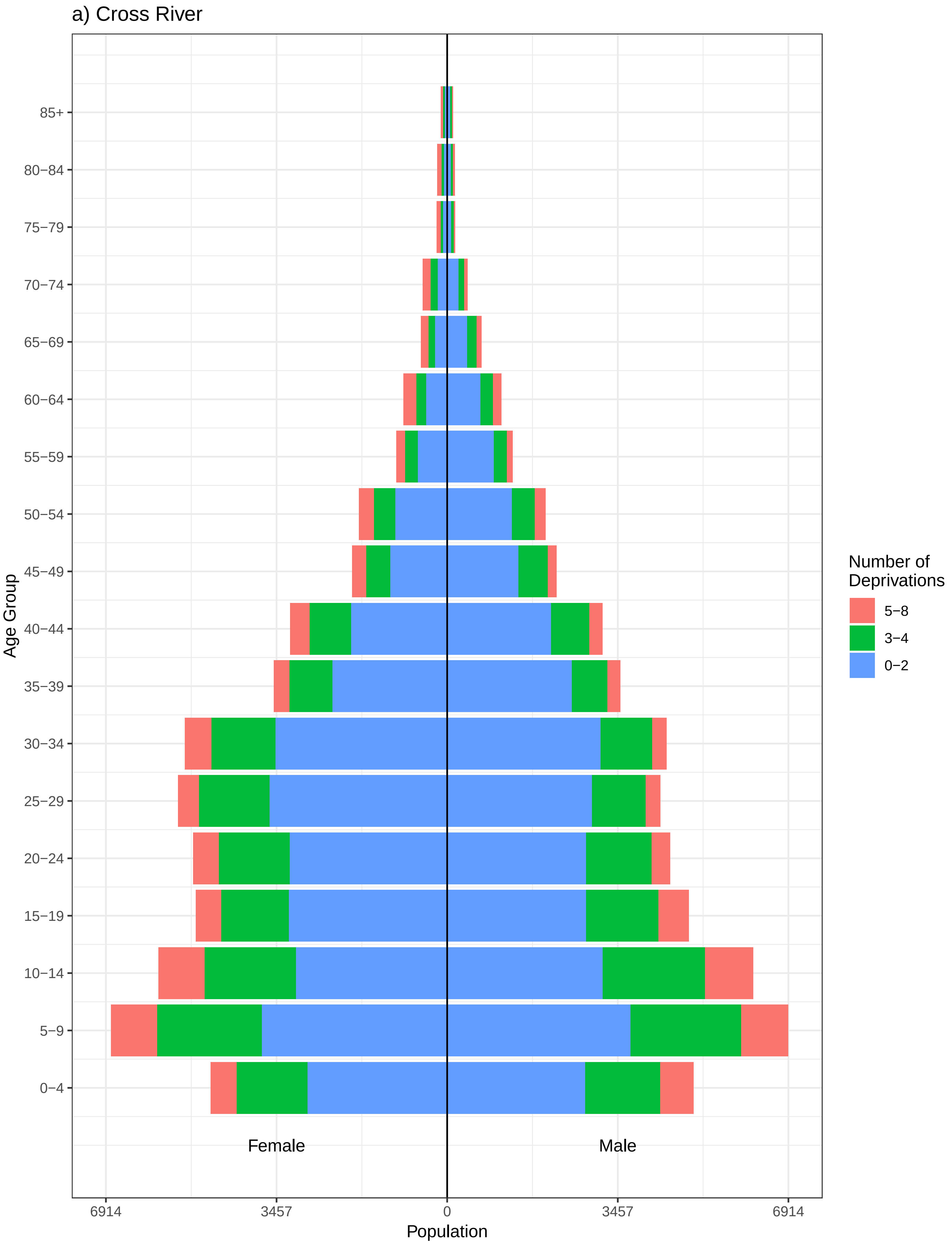

a) Cross River
Age Group
85+
80−84
75−79
70−74
65−69
60−64
55−59
50−54
45−49
40−44
35−39
30−34
25−29
20−24
15−19
10−14
5−9
0−4
Female
Male
6914
3457
0
3457
6914
Population
Number of Deprivations
5−8
3−4
0−2

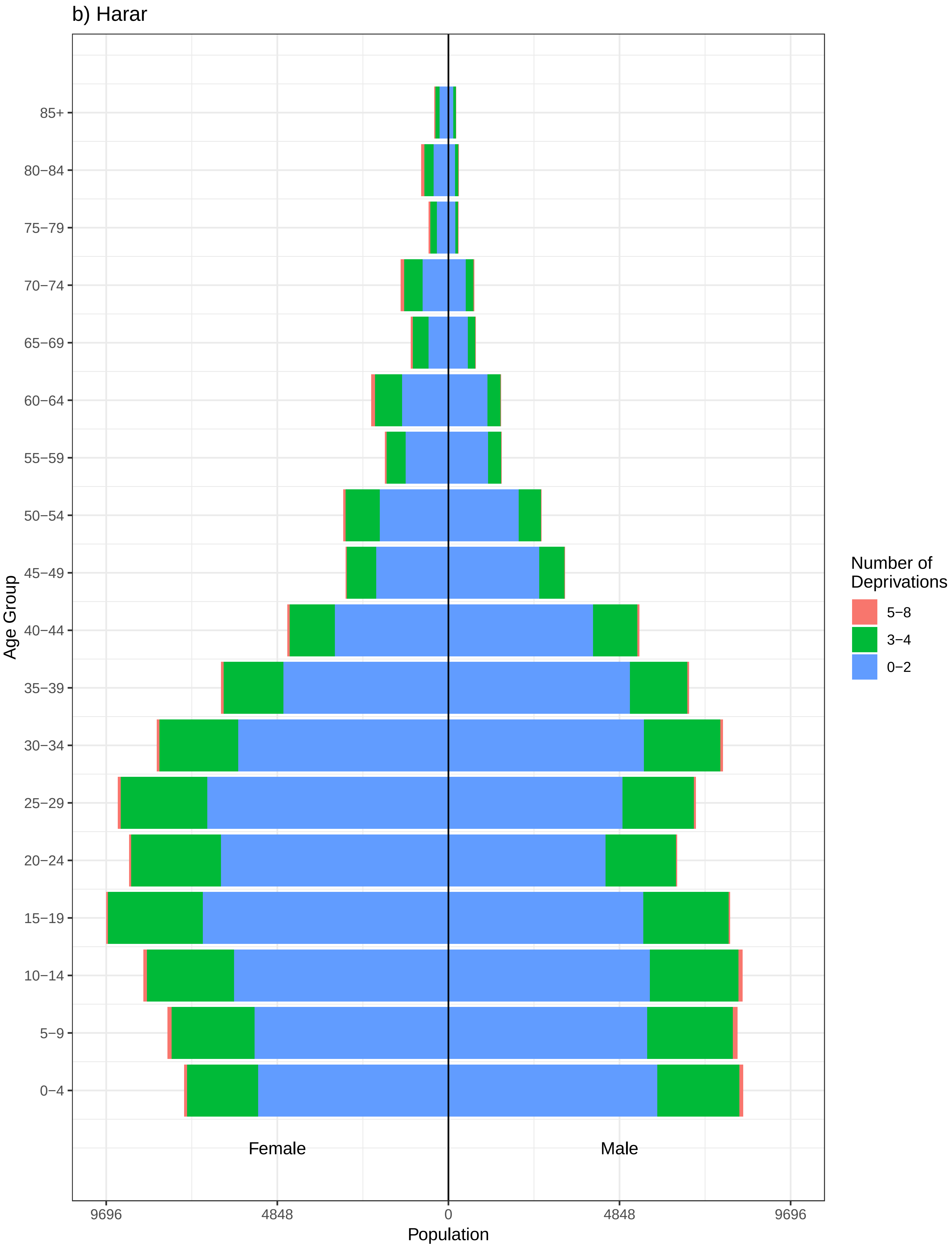

b) Harar
Age Group
85+
80−84
75−79
70−74
65−69
60−64
55−59
50−54
45−49
40−44
35−39
30−34
25−29
20−24
15−19
10−14
5−9
0−4
Female
Male
9696
4848
0
4848
9696
Population
Number of Deprivations
5−8
3−4
0−2

c) Karonga

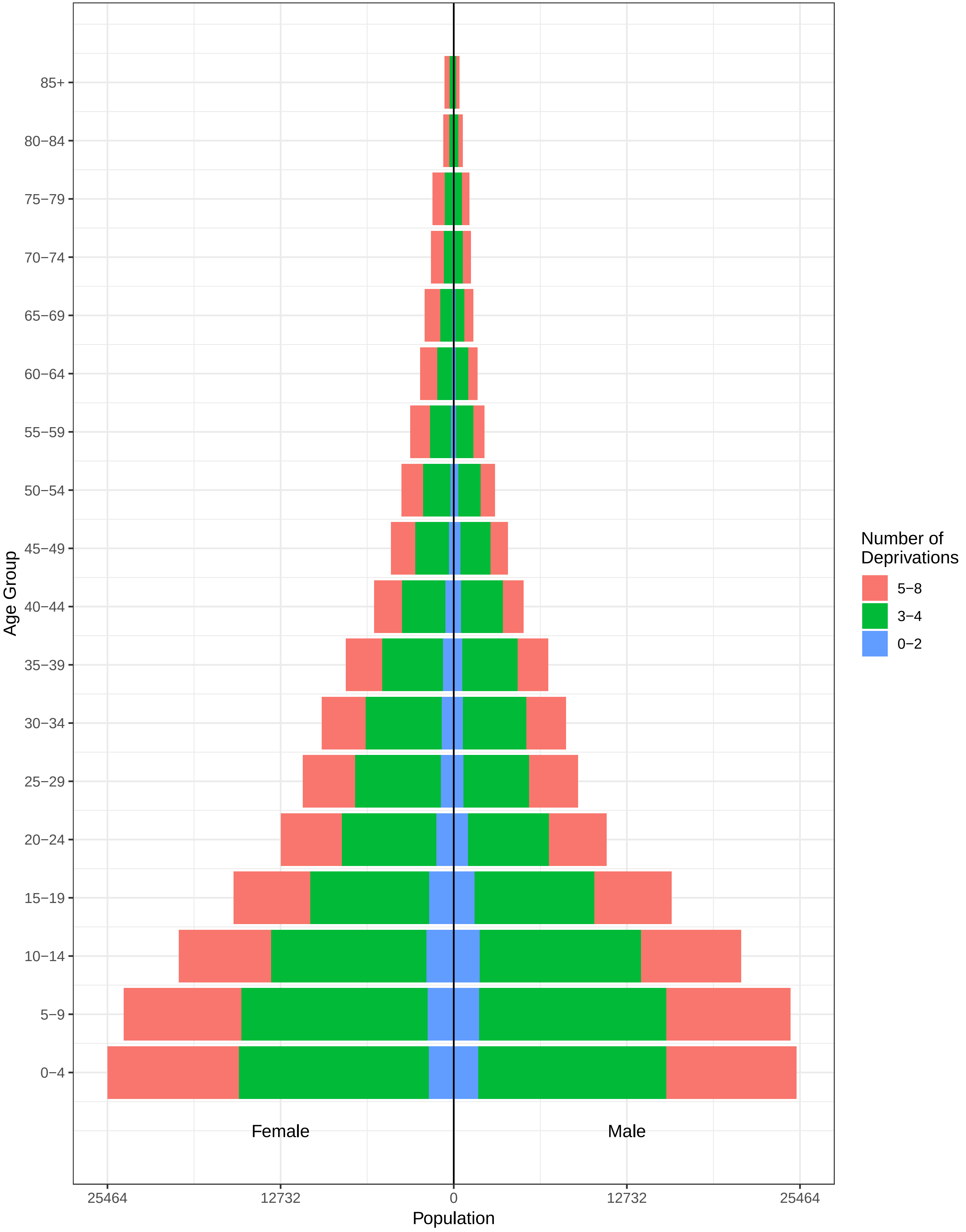
85+
80−84
75−79
70−74
65−69
60−64
55−59
50−54
45−49
40−44
35−39
30−34
25−29
20−24
15−19
10−14
5−9
0−4
Age Group
Female
Male
25464
12732
0
12732
25464
Population
Number of Deprivations
5−8
3−4
0−2

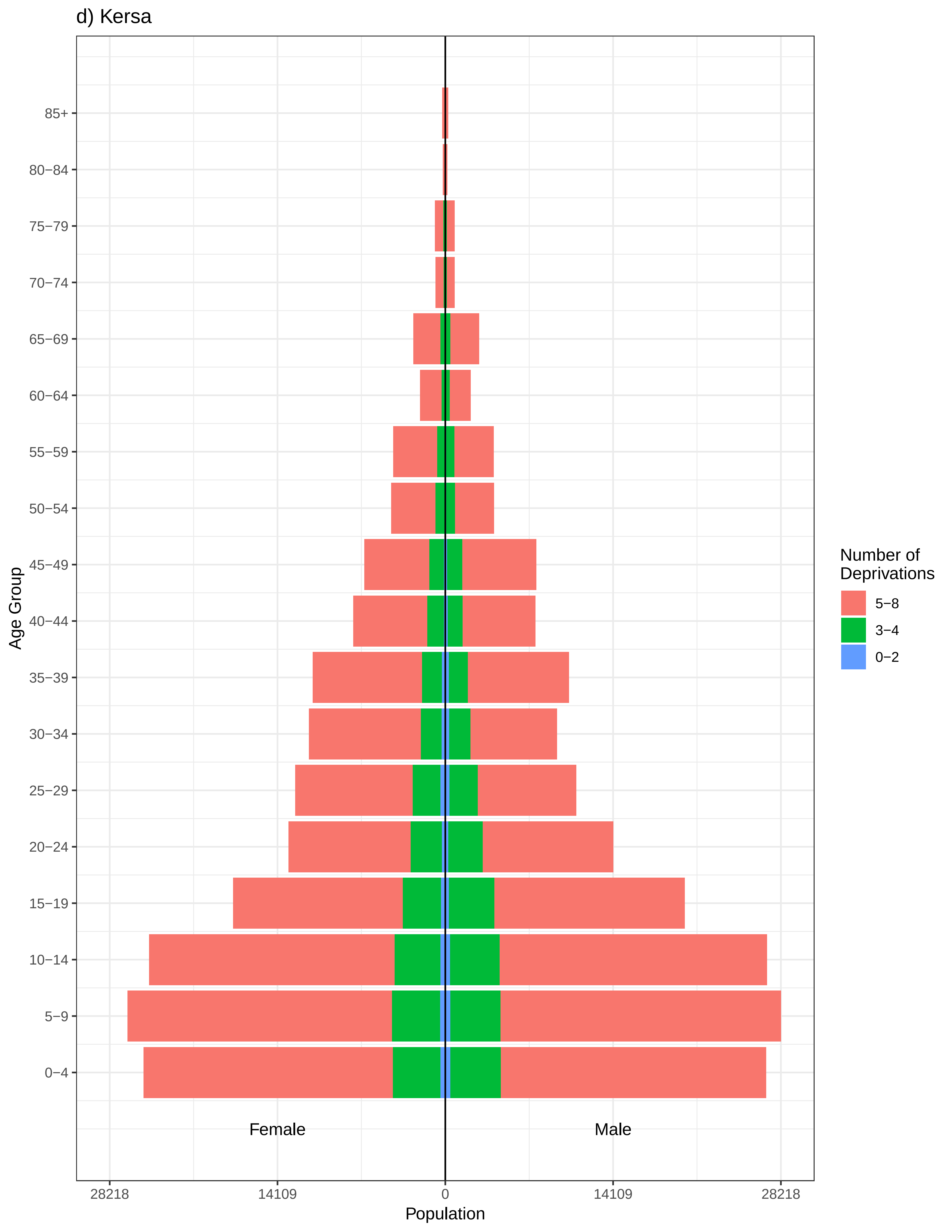
d) Kersa
Age Group
85+
80−84
75−79
70−74
65−69
60−64
55−59
50−54
45−49
40−44
35−39
30−34
25−29
20−24
15−19
10−14
5−9
0−4
Female
Male
28218
14109
0
14109
28218
Population
Number of Deprivations
5−8
3−4
0−2

e) Kombewa

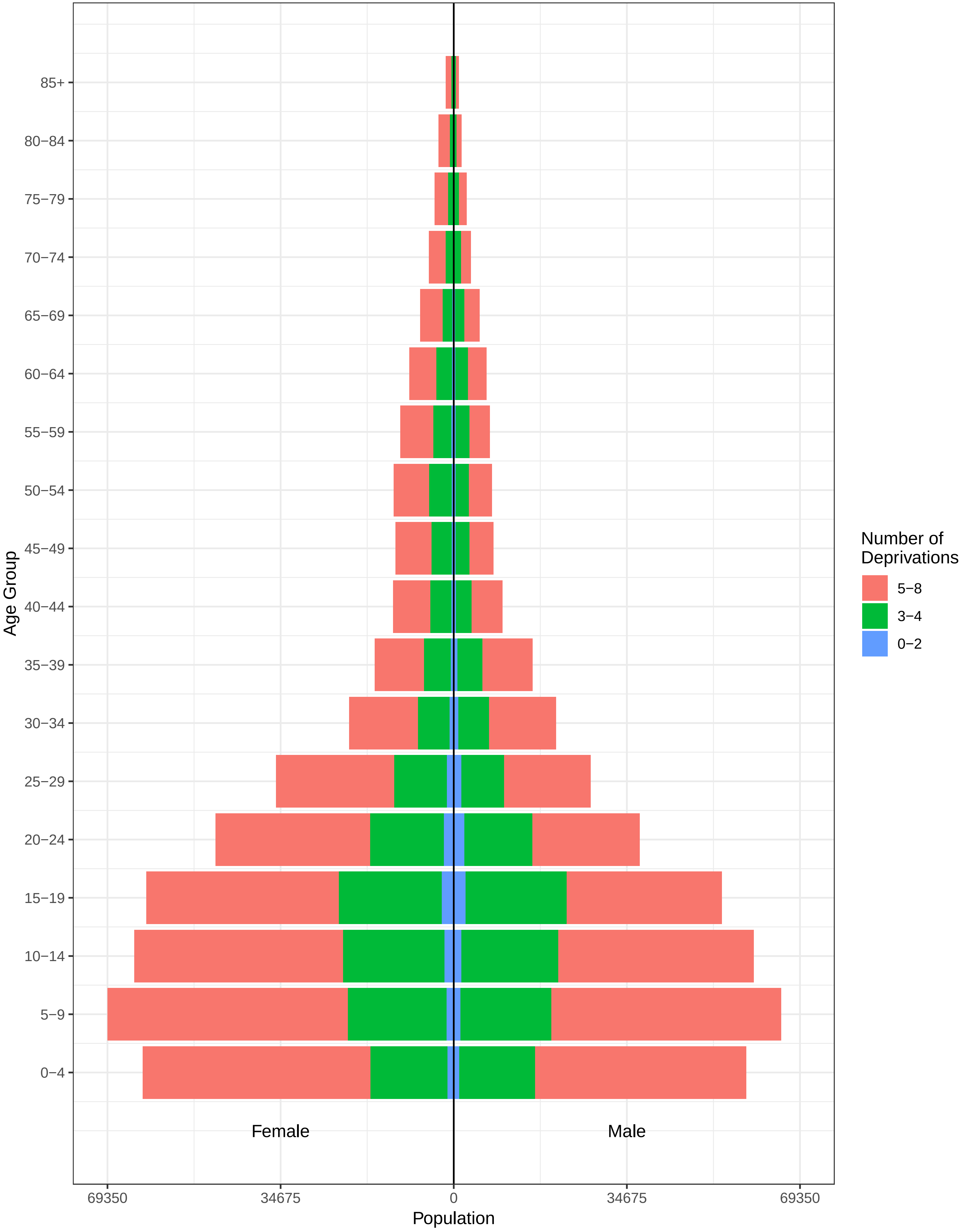

85+
80−84
75−79
70−74
65−69
60−64
55−59
50−54
45−49
40−44
35−39
30−34
25−29
20−24
15−19
10−14
5−9
0−4
Age Group
Female
Male
69350
34675
0
34675
69350
Population
Number of Deprivations
5−8
3−4
0−2

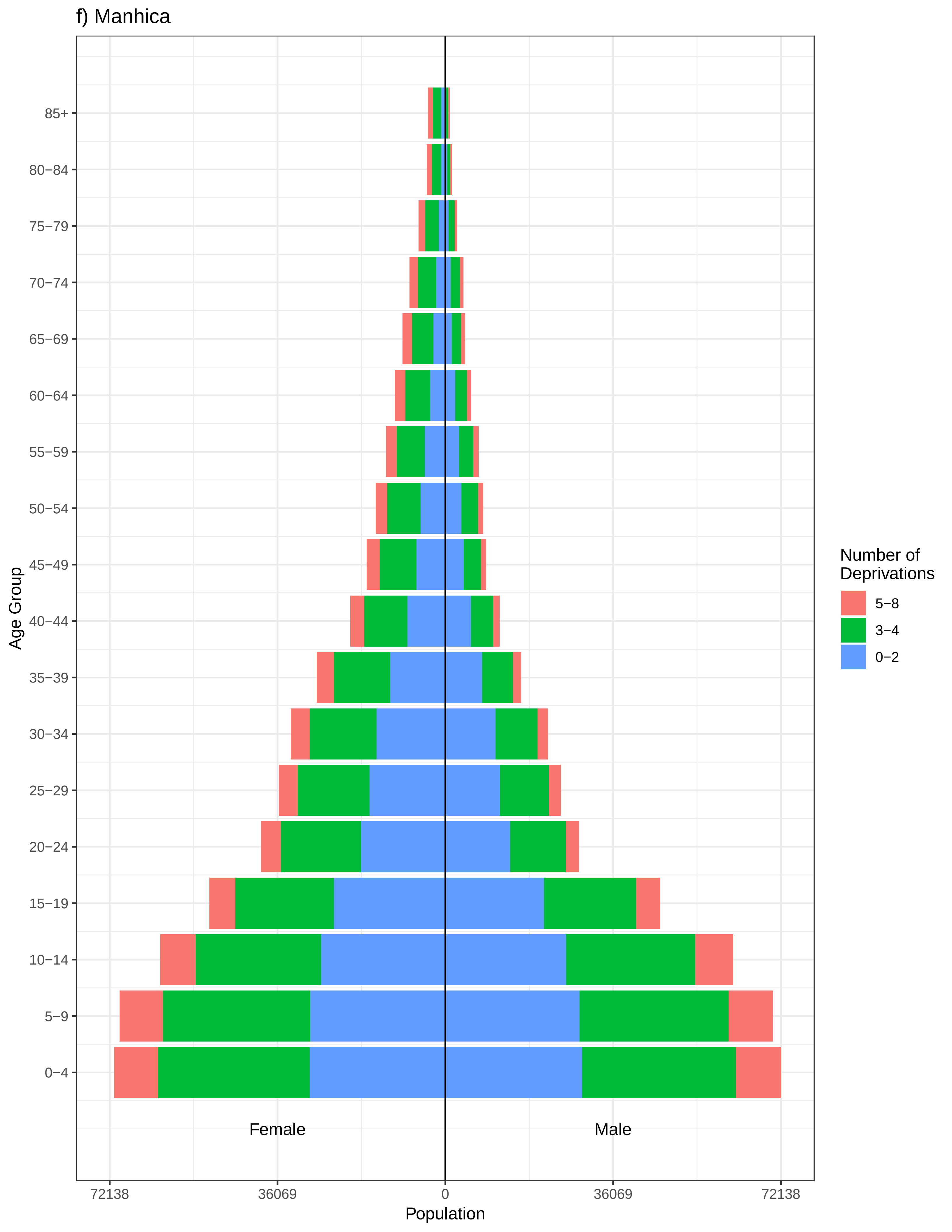

f) Manhica
85+
80−84
75−79
70−74
65−69
60−64
55−59
50−54
45−49
40−44
35−39
30−34
25−29
20−24
15−19
10−14
5−9
0−4
Age Group
Female
Male
72138
36069
0
36069
72138
Population
Number of Deprivations
5−8
3−4
0−2

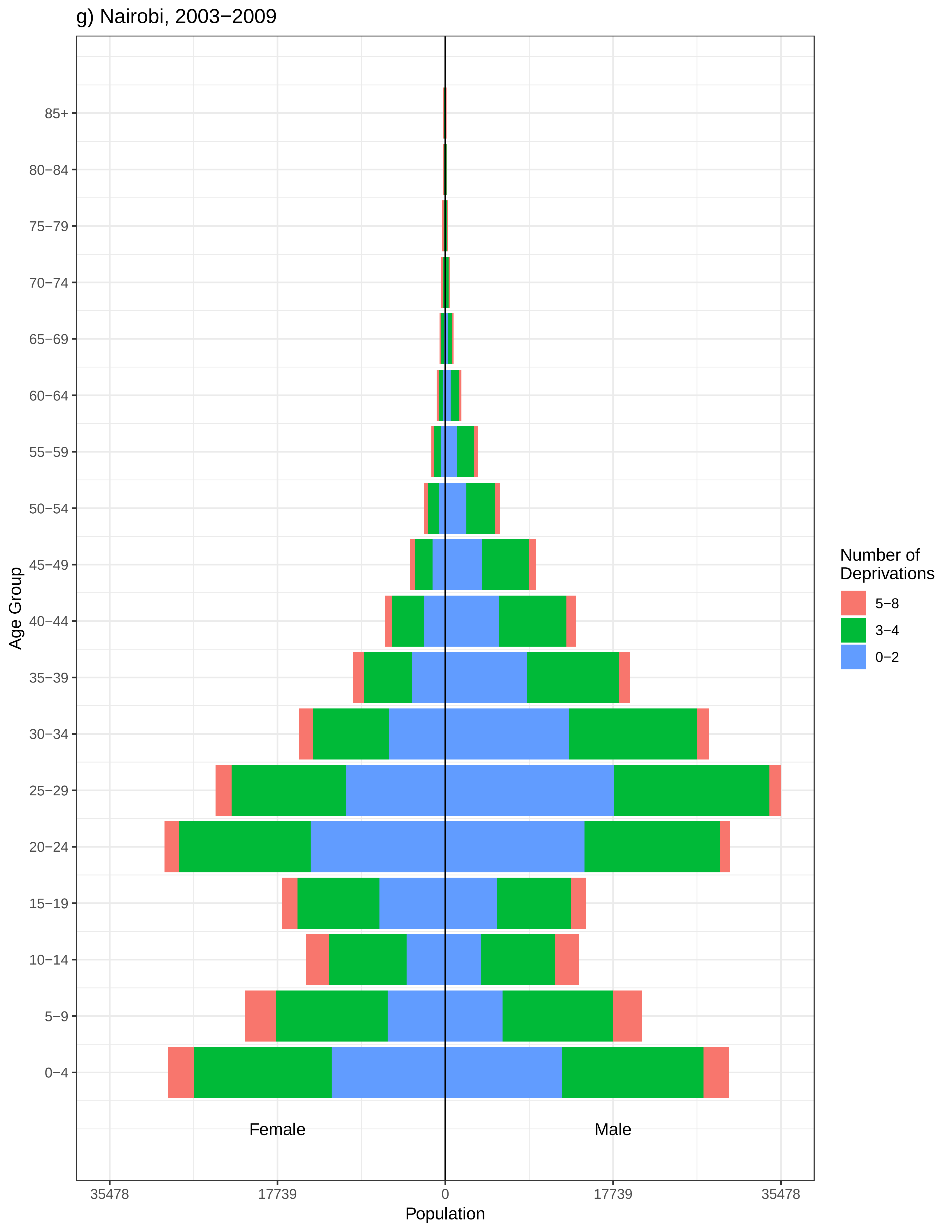

g) Nairobi, 2003−2009
85+
80−84
75−79
70−74
65−69
60−64
55−59
50−54
45−49
40−44
35−39
30−34
25−29
20−24
15−19
10−14
5−9
0−4
Age Group
Female
Male
35478
17739
0
17739
35478
Population
Number of Deprivations
5−8
3−4
0−2

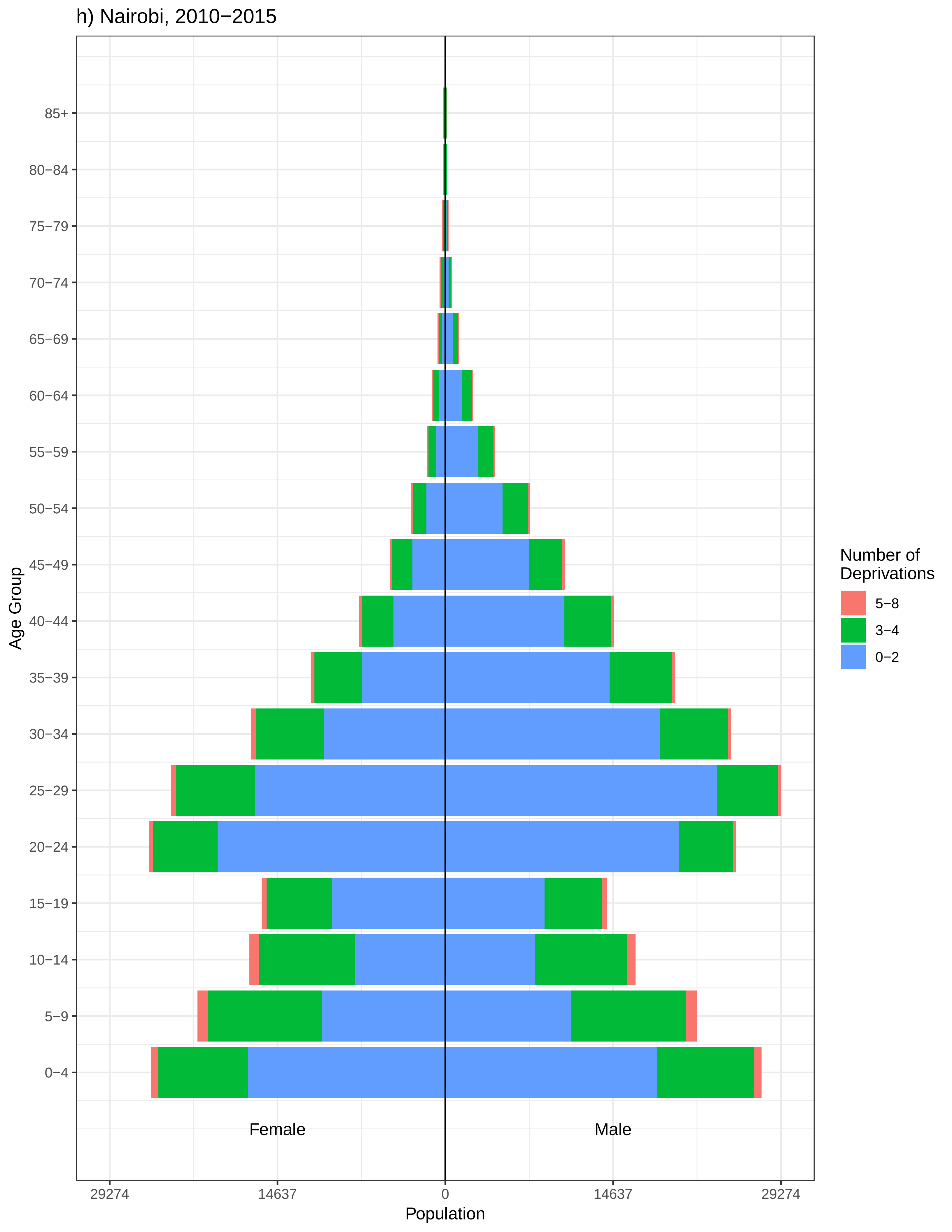

h) Nairobi, 2010−2015
Age Group
85+
80−84
75−79
70−74
65−69
60−64
55−59
50−54
45−49
40−44
35−39
30−34
25−29
20−24
15−19
10−14
5−9
0−4
Female
Male
29274
14637
0
14637
29274
Population
Number of Deprivations
5−8
3−4
0−2

Figure S2:

Proportion of population in each age and sex group, within each deprivation group

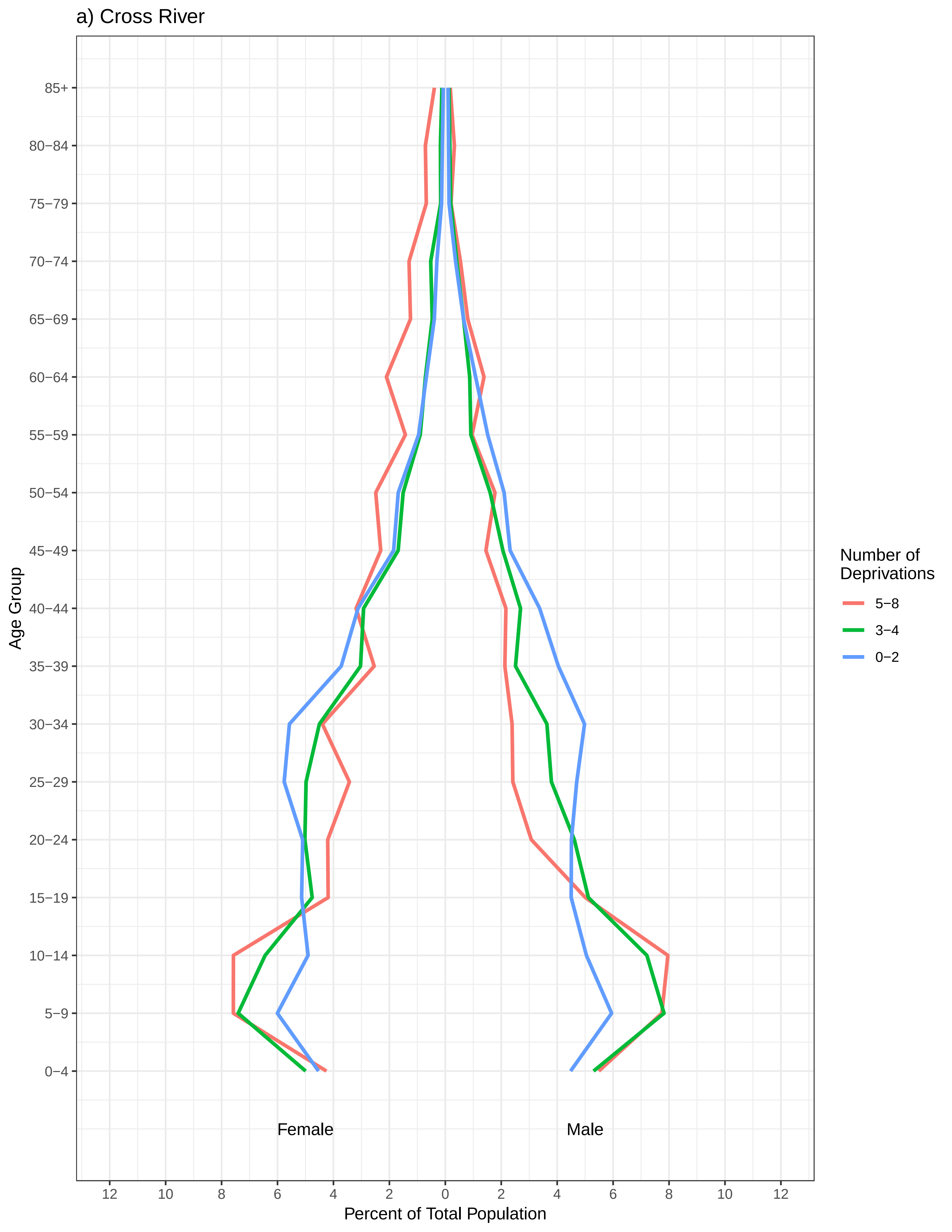
a) Cross River
Age Group
85+
80–84
75–79
70–74
65–69
60–64
55–59
50–54
45–49
40–44
35–39
30–34
25–29
20–24
15–19
10–14
5–9
0–4
Female
Male
12
10
8
6
4
2
0
2
4
6
8
10
12
Percent of Total Population
Number of Deprivations
5–8
3–4
0–2

b) Harar

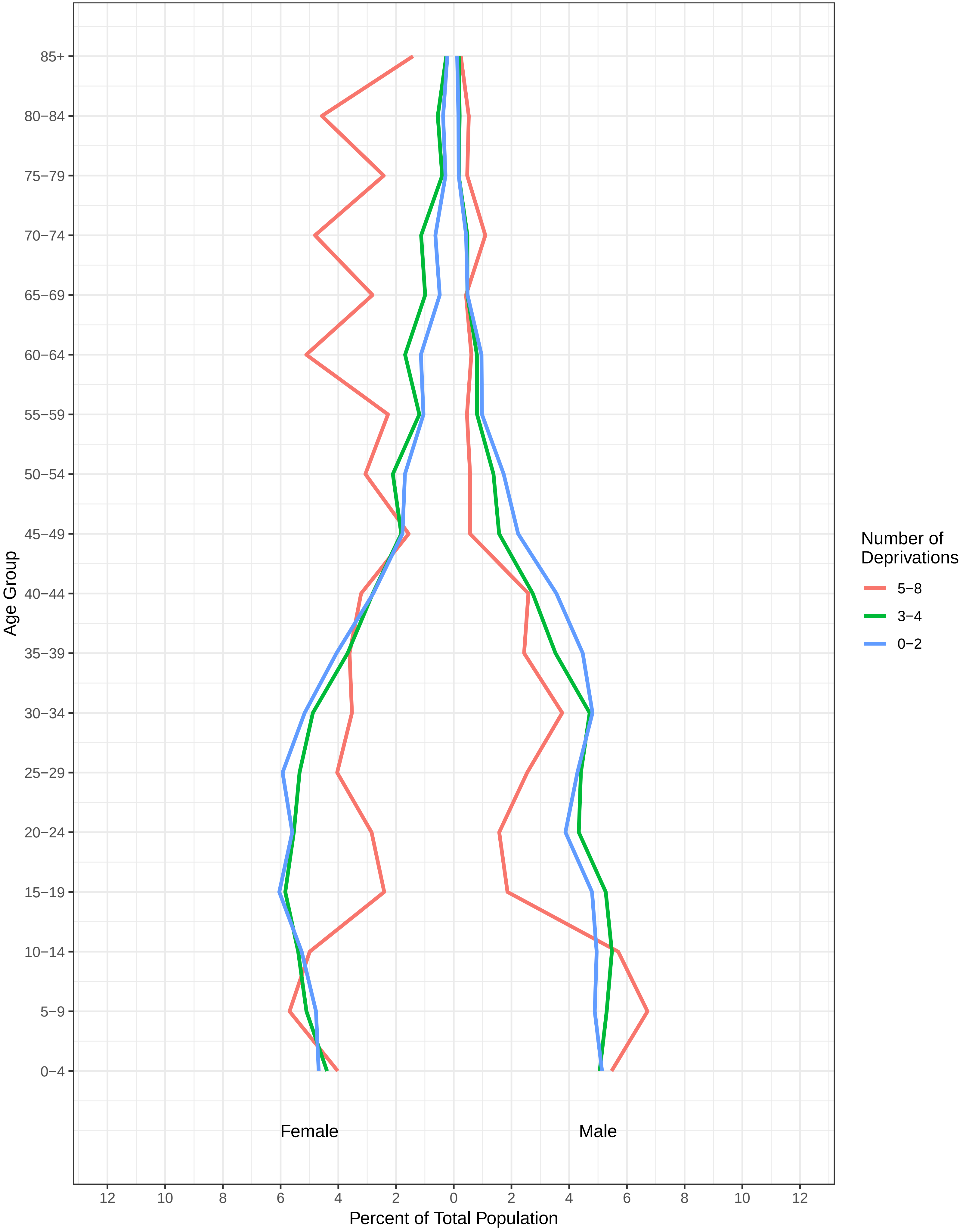

85+
80−84
75−79
70−74
65−69
60−64
55−59
50−54
45−49
40−44
35−39
30−34
25−29
20−24
15−19
10−14
5−9
0−4
Age Group
Female
Male
12 10 8 6 4 2 0 2 4 6 8 10 12
Percent of Total Population
Number of Deprivations
5−8
3−4
0−2

c) Karonga

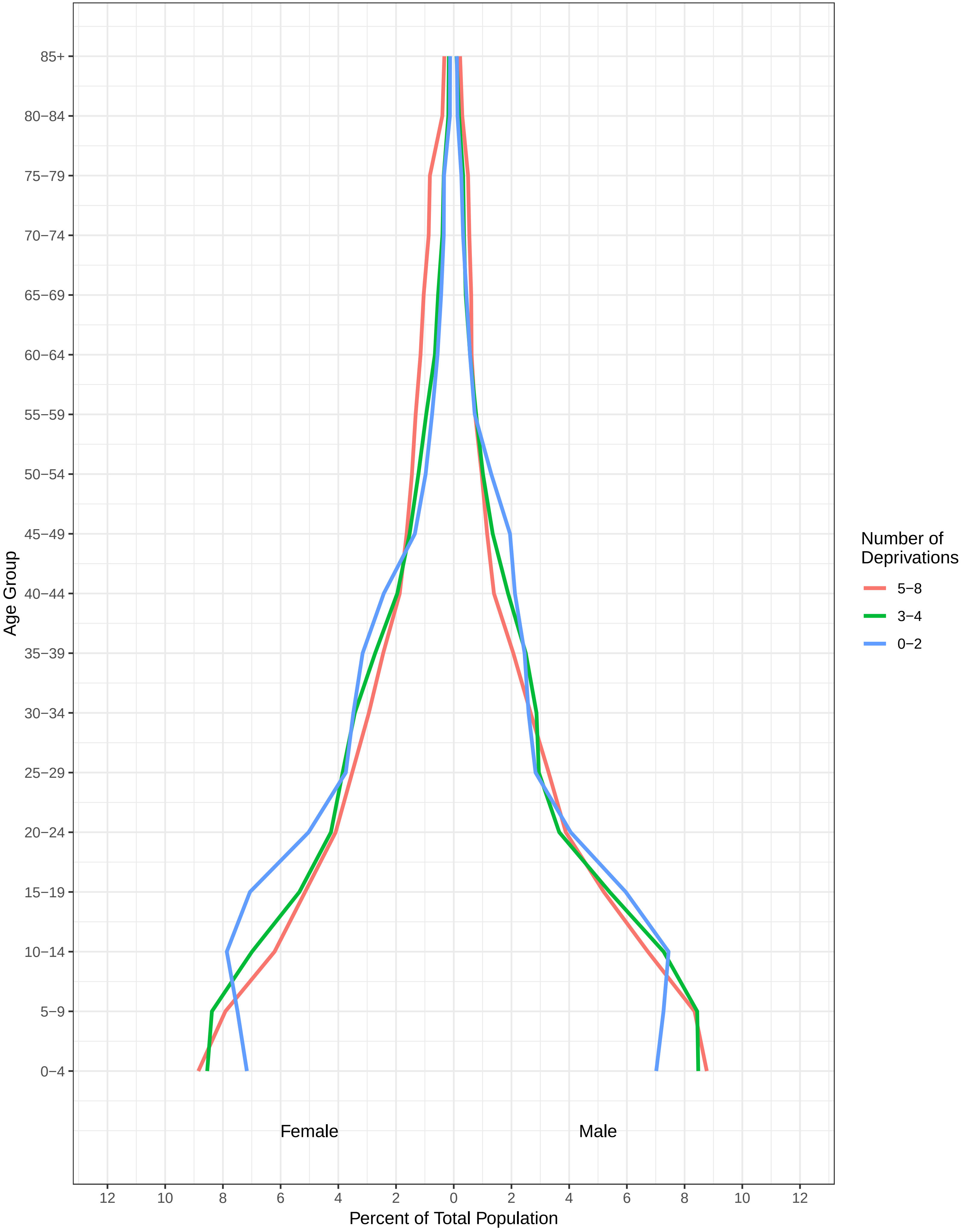

85+
80−84
75−79
70−74
65−69
60−64
55−59
50−54
45−49
40−44
35−39
30−34
25−29
20−24
15−19
10−14
5−9
0−4
Age Group
Female
Male
12 10 8 6 4 2 0 2 4 6 8 10 12
Percent of Total Population
Number of Deprivations
5−8
3−4
0−2

d) Kersa

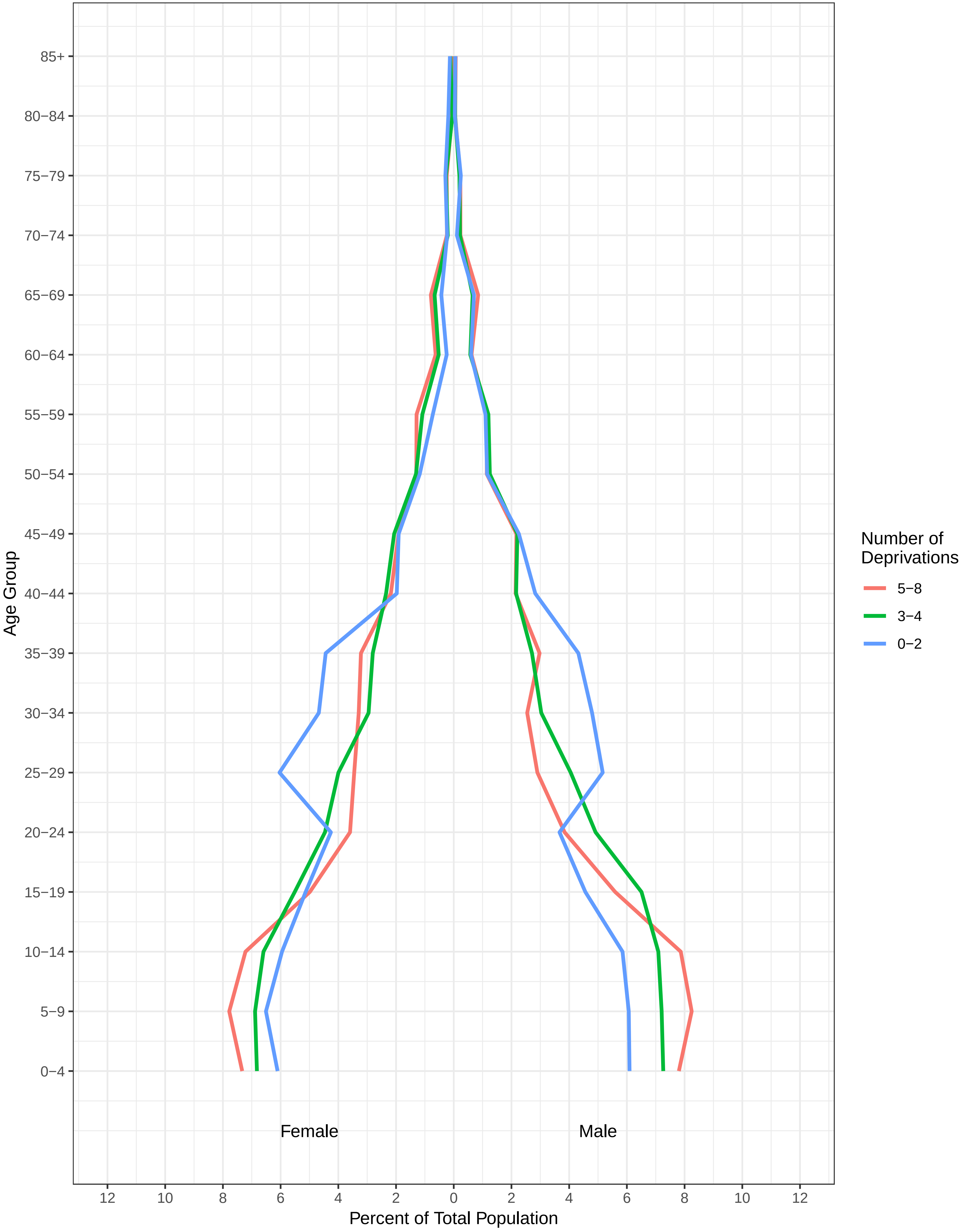

85+
80−84
75−79
70−74
65−69
60−64
55−59
50−54
45−49
40−44
35−39
30−34
25−29
20−24
15−19
10−14
5−9
0−4
Age Group
Female
Male
12 10 8 6 4 2 0 2 4 6 8 10 12
Percent of Total Population
Number of Deprivations
5−8
3−4
0−2

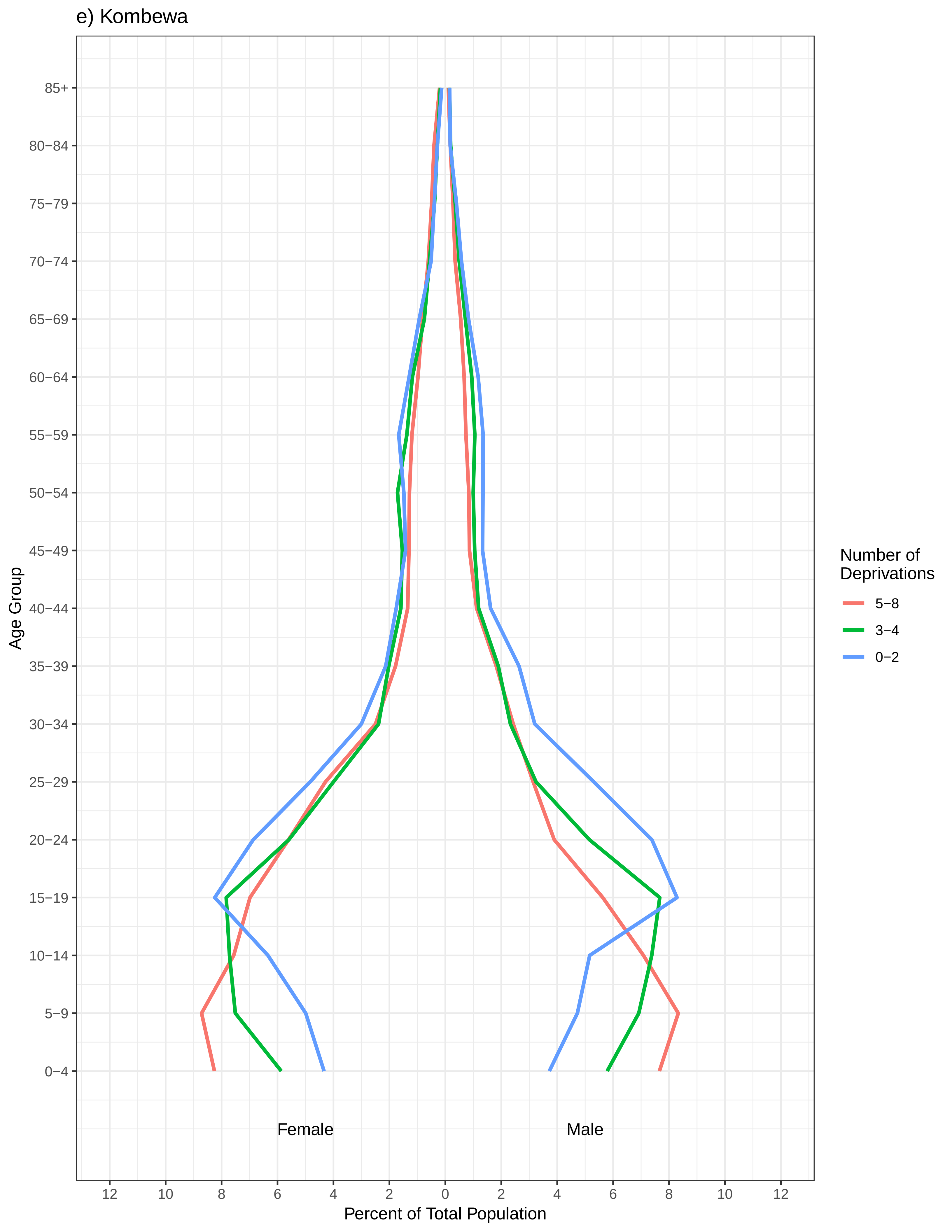

e) Kombewa
85+
80−84
75−79
70−74
65−69
60−64
55−59
50−54
45−49
40−44
35−39
30−34
25−29
20−24
15−19
10−14
5−9
0−4
Age Group
Female
Male
12 10 8 6 4 2 0 2 4 6 8 10 12
Percent of Total Population
Number of Deprivations
5−8
3−4
0−2

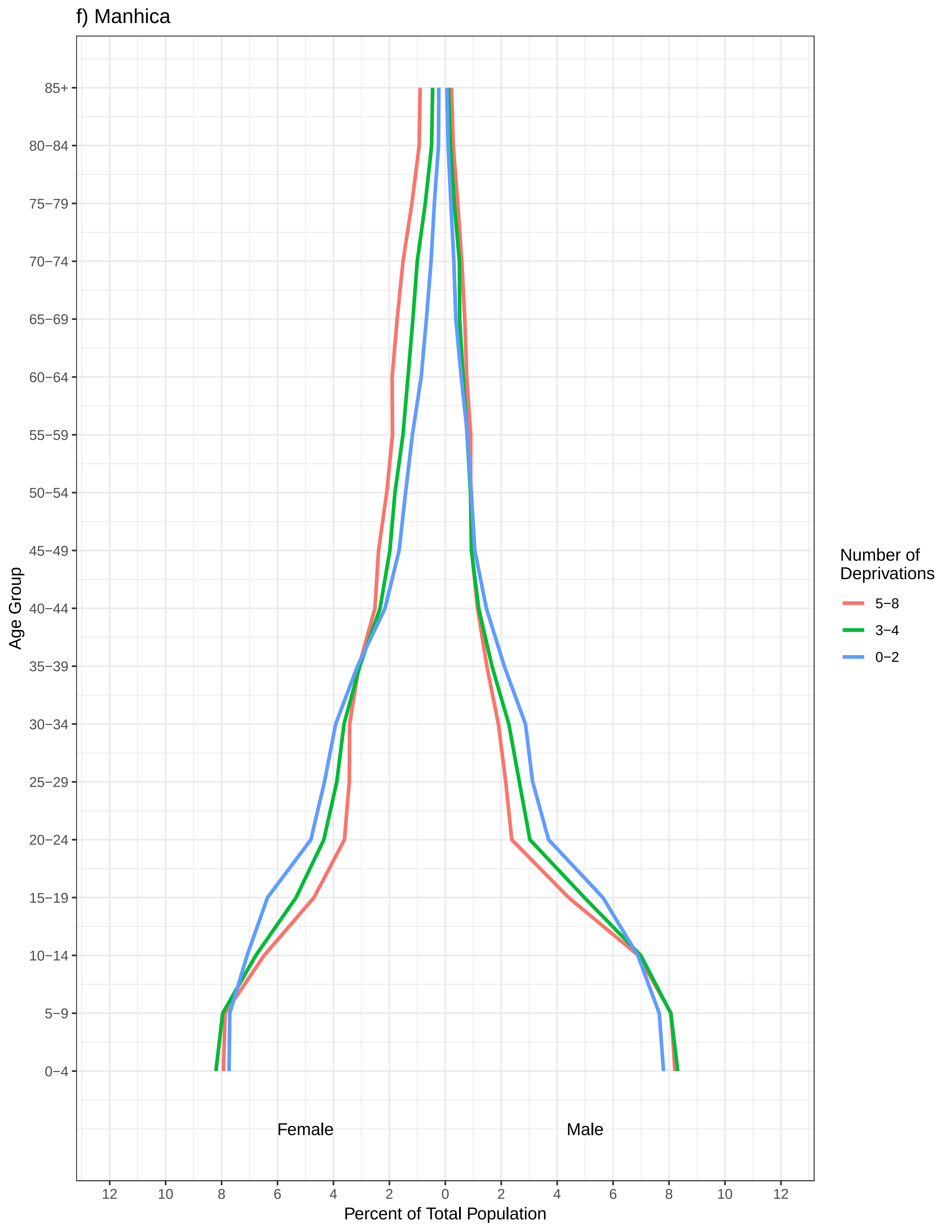

f) Manhica
85+
80−84
75−79
70−74
65−69
60−64
55−59
50−54
45−49
40−44
35−39
30−34
25−29
20−24
15−19
10−14
5−9
0−4
Age Group
Female
Male
12
10
8
6
4
2
0
2
4
6
8
10
12
Percent of Total Population
Number of Deprivations
5−8
3−4
0−2

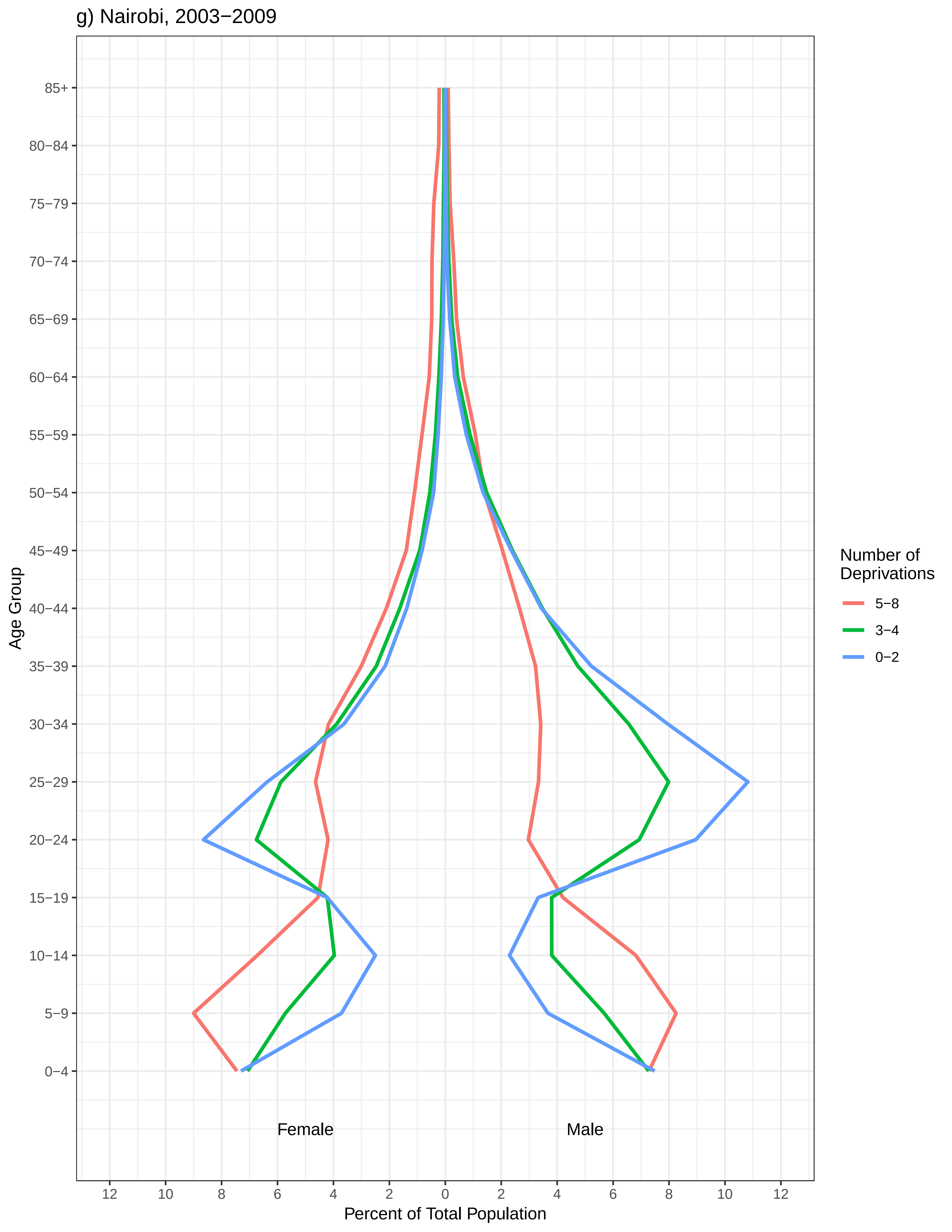

g) Nairobi, 2003−2009
85+
80−84
75−79
70−74
65−69
60−64
55−59
50−54
45−49
40−44
35−39
30−34
25−29
20−24
15−19
10−14
5−9
0−4
Age Group
Female
Male
12
10
8
6
4
2
0
2
4
6
8
10
12
Percent of Total Population
Number of Deprivations
5−8
3−4
0−2

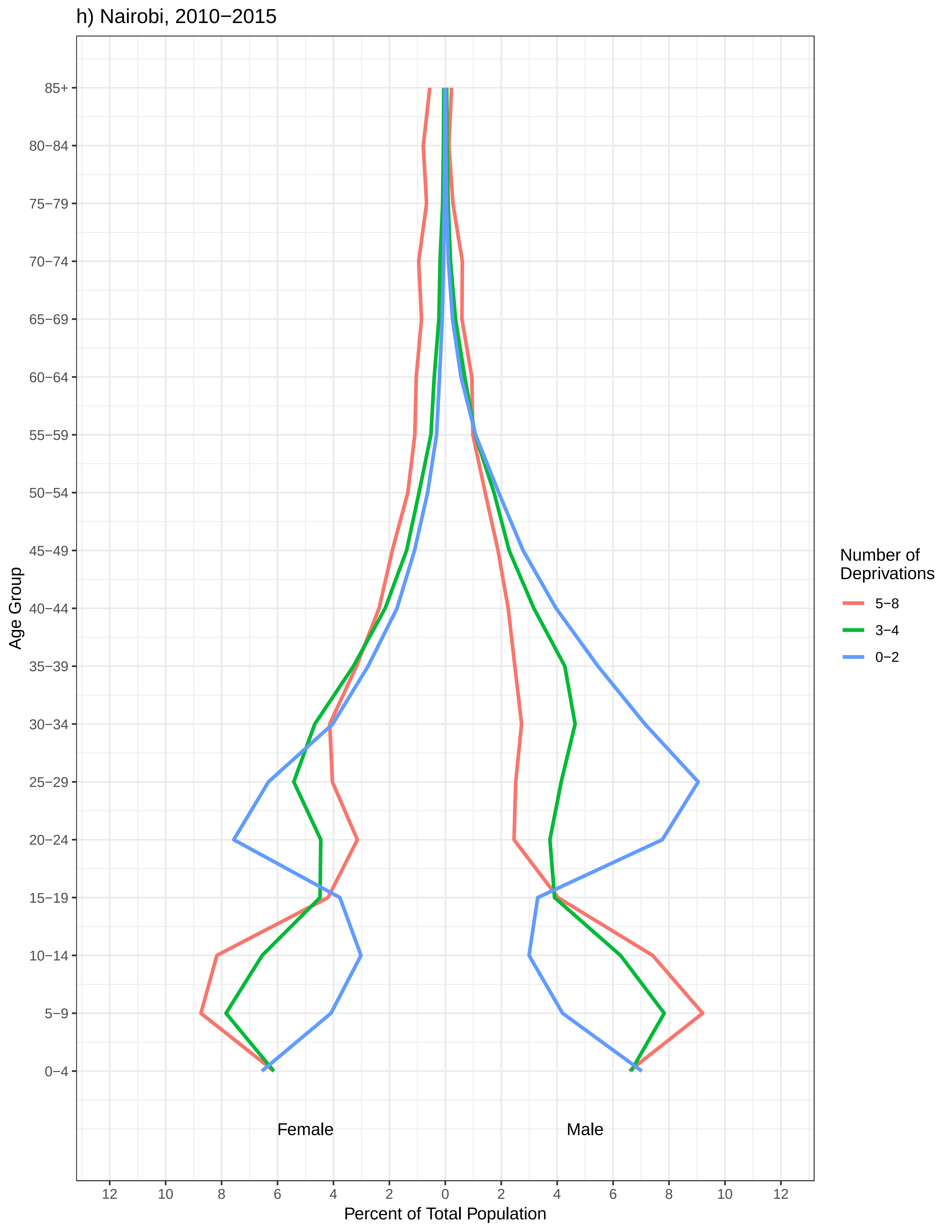

h) Nairobi, 2010−2015
85+
80−84
75−79
70−74
65−69
60−64
55−59
50−54
45−49
40−44
35−39
30−34
25−29
20−24
15−19
10−14
5−9
0−4
Age Group
Female
Male
12
10
8
6
4
2
0
2
4
6
8
10
12
Percent of Total Population
Number of Deprivations
5−8
3−4
0−2

Figure S3:

Proportion of population in each deprivation group, within each age and sex group

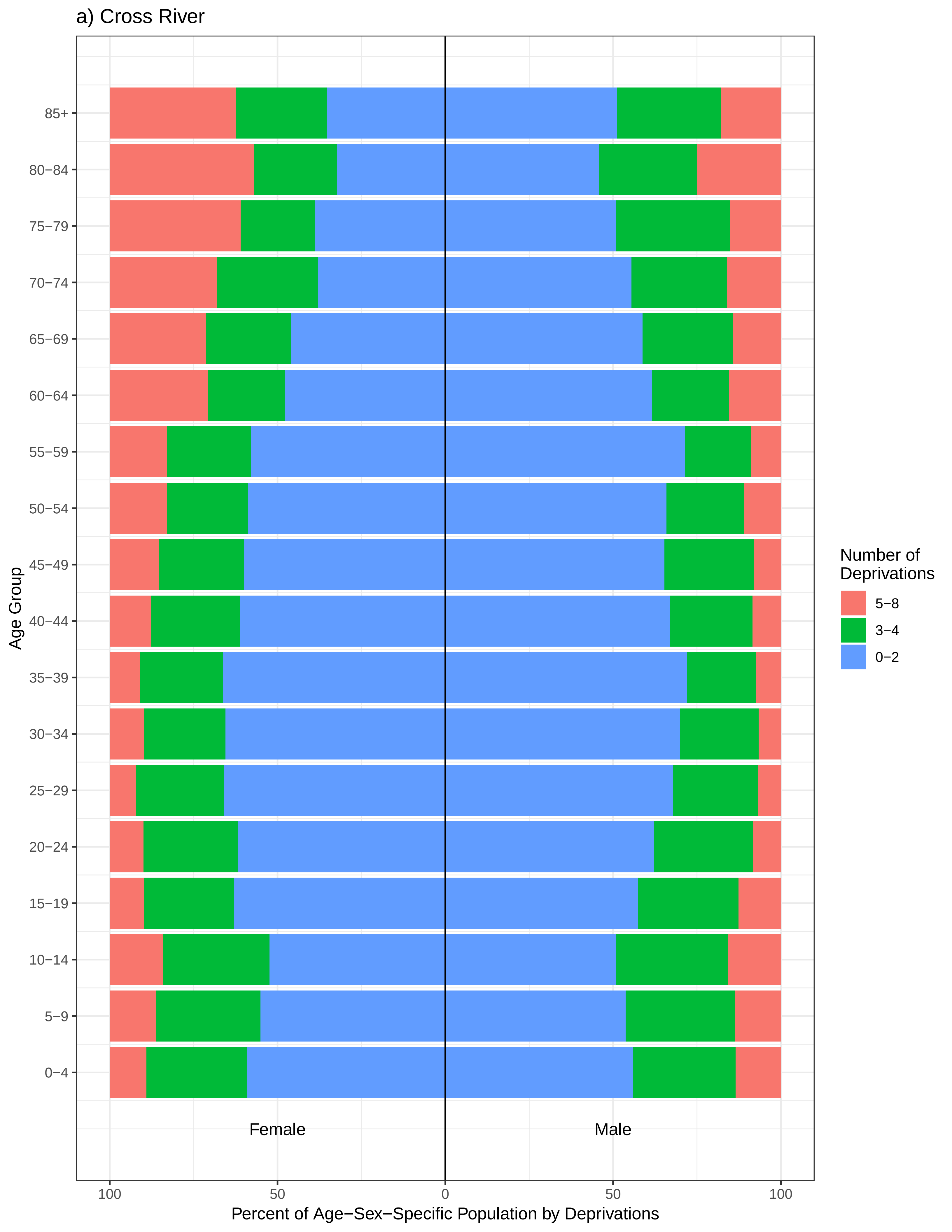

a) Cross River
85+
80−84
75−79
70−74
65−69
60−64
55−59
50−54
45−49
40−44
35−39
30−34
25−29
20−24
15−19
10−14
5−9
0−4
Age Group
Female
Male
100
50
0
50
100
Percent of Age−Sex−Specific Population by Deprivations
Number of Deprivations
5−8
3−4
0−2

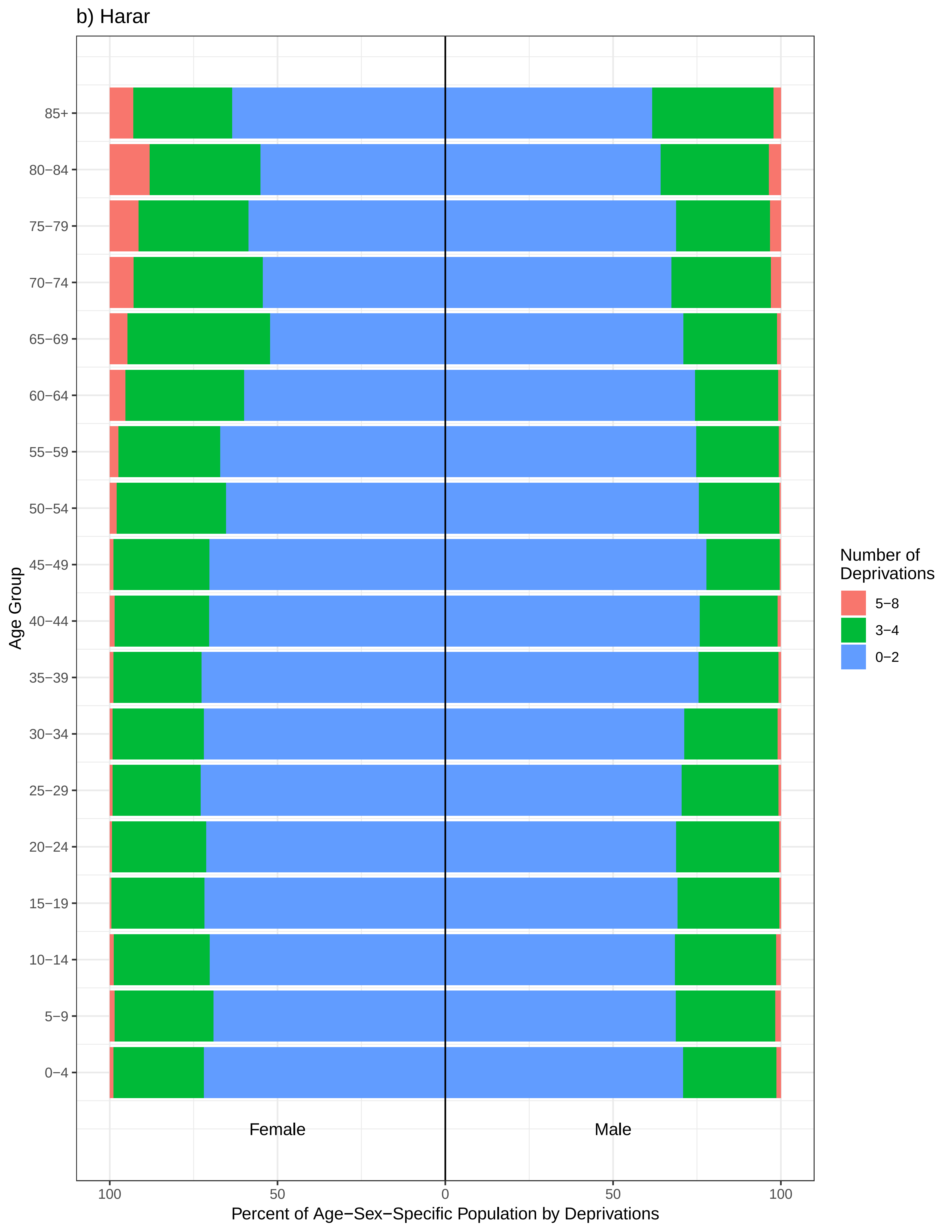
b) Harar
85+
80−84
75−79
70−74
65−69
60−64
55−59
50−54
45−49
40−44
35−39
30−34
25−29
20−24
15−19
10−14
5−9
0−4
Age Group
Female
Male
100
50
0
50
100
Percent of Age−Sex−Specific Population by Deprivations
Number of Deprivations
5−8
3−4
0−2

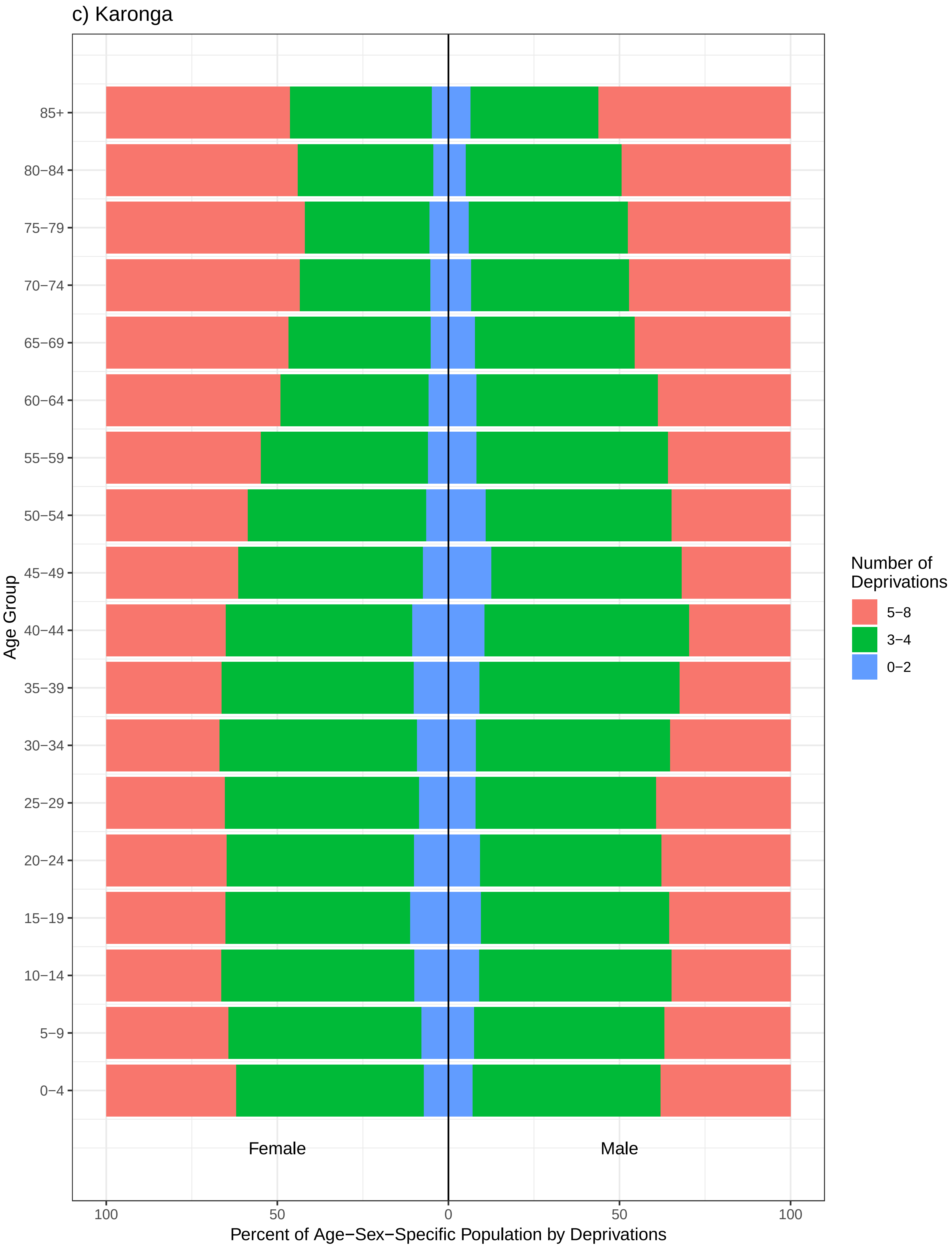
c) Karonga
85+
80−84
75−79
70−74
65−69
60−64
55−59
50−54
45−49
40−44
35−39
30−34
25−29
20−24
15−19
10−14
5−9
0−4
Age Group
Female
Male
100
50
0
50
100
Percent of Age−Sex−Specific Population by Deprivations
Number of Deprivations
5−8
3−4
0−2

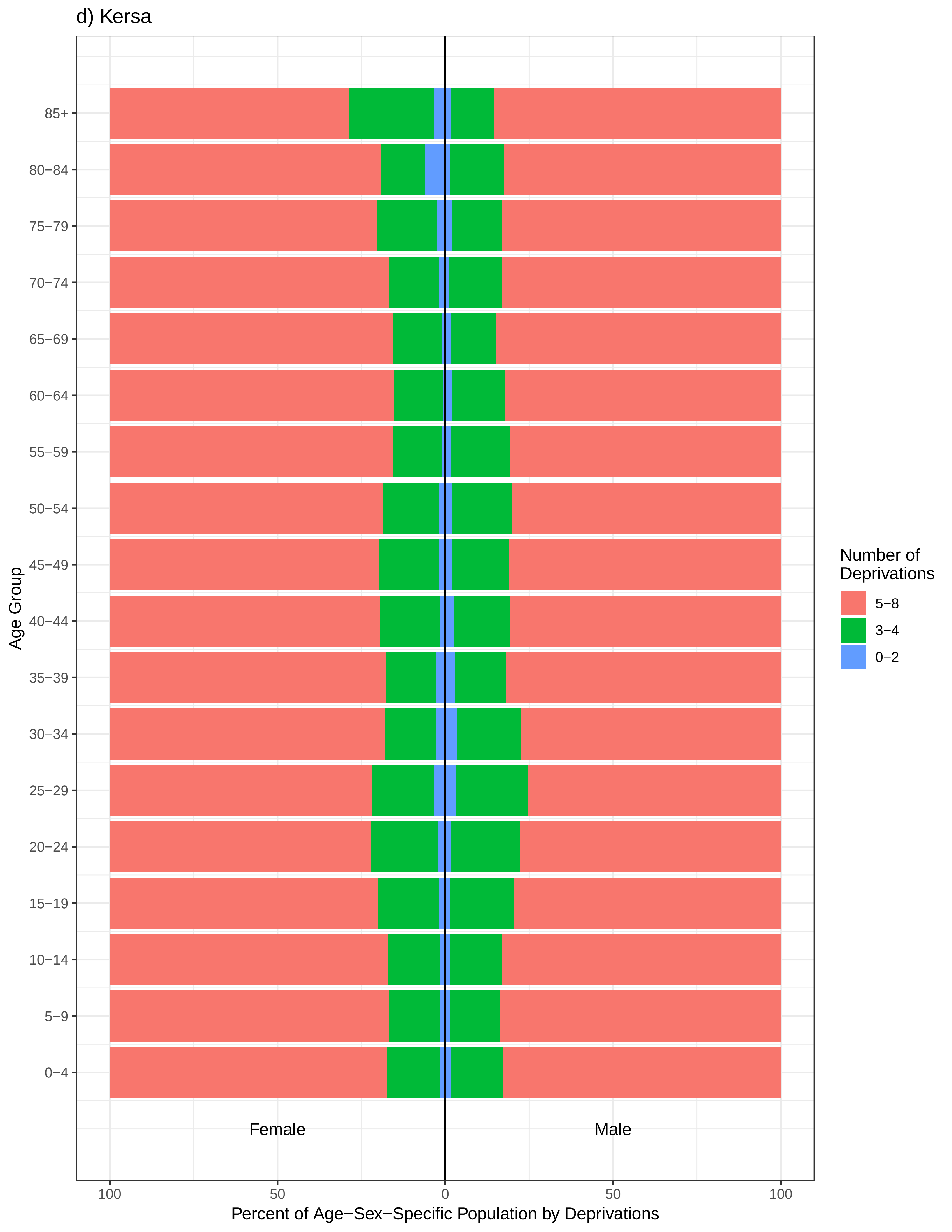
d) Kersa
85+
80−84
75−79
70−74
65−69
60−64
55−59
50−54
45−49
40−44
35−39
30−34
25−29
20−24
15−19
10−14
5−9
0−4
Age Group
Female
Male
100
50
0
50
100
Percent of Age−Sex−Specific Population by Deprivations
Number of Deprivations
5−8
3−4
0−2

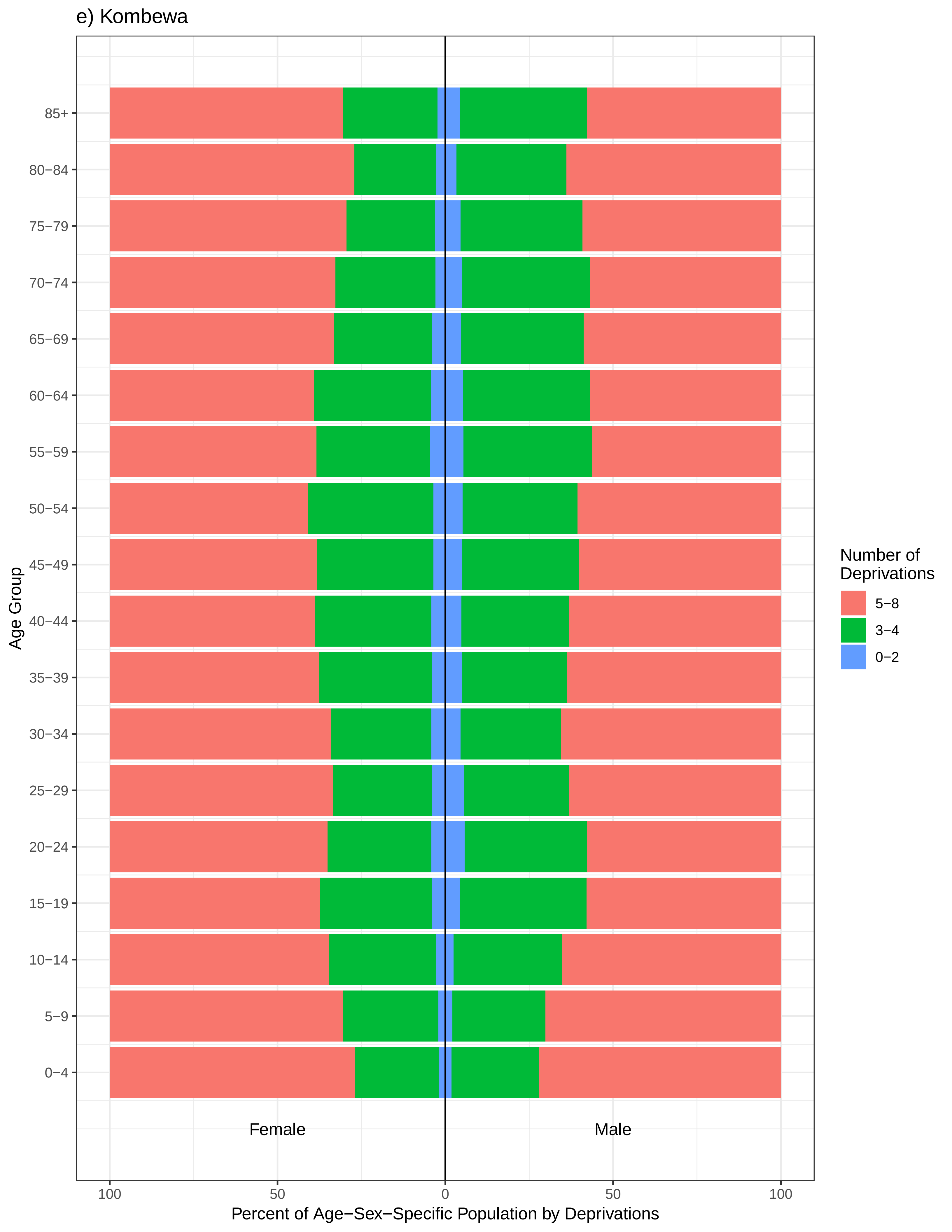

e) Kombewa
85+
80−84
75−79
70−74
65−69
60−64
55−59
50−54
45−49
40−44
35−39
30−34
25−29
20−24
15−19
10−14
5−9
0−4
Age Group
Female
Male
100
50
0
50
100
Percent of Age−Sex−Specific Population by Deprivations
Number of Deprivations
5−8
3−4
0−2

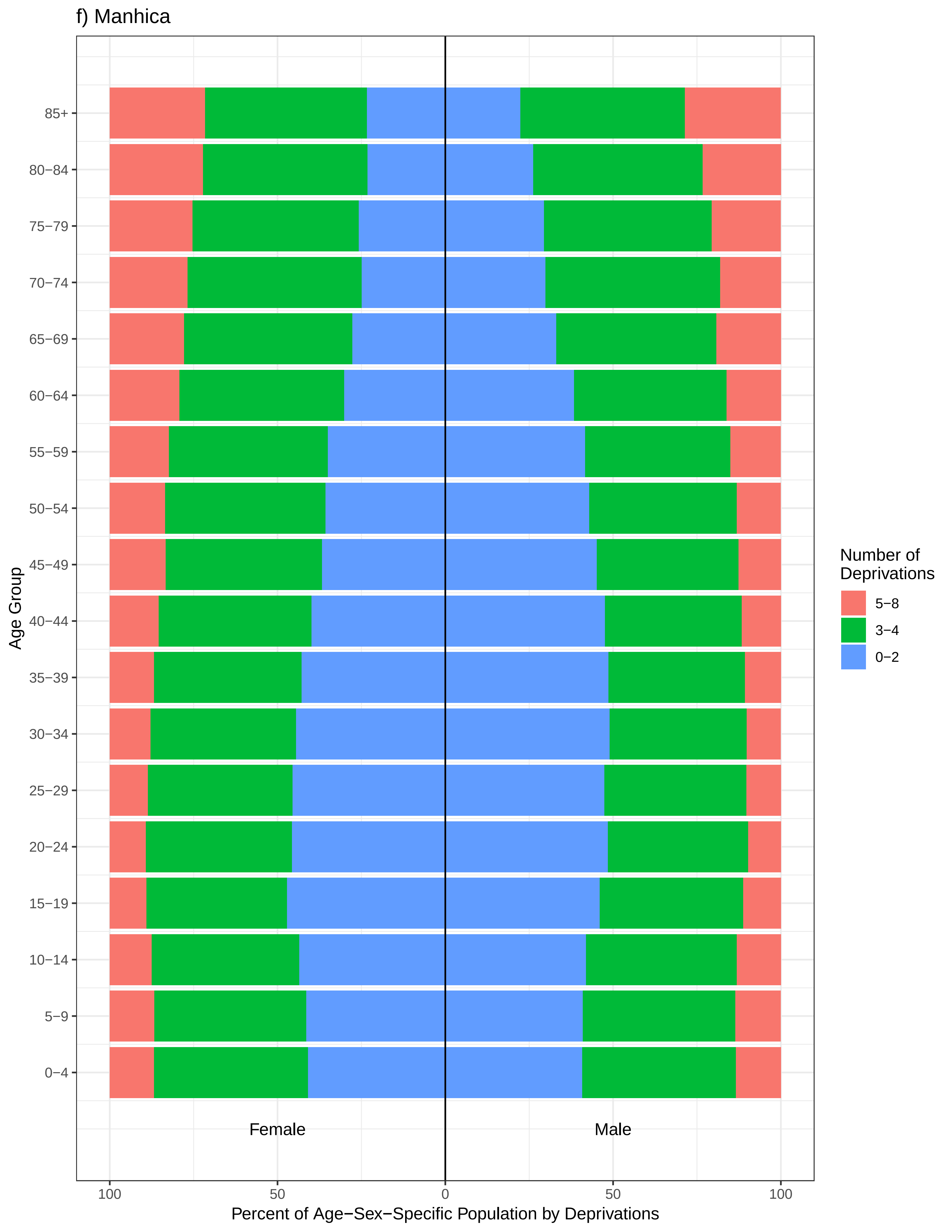

f) Manhica
85+
80−84
75−79
70−74
65−69
60−64
55−59
50−54
45−49
40−44
35−39
30−34
25−29
20−24
15−19
10−14
5−9
0−4
Age Group
Female
Male
100
50
0
50
100
Percent of Age−Sex−Specific Population by Deprivations
Number of Deprivations
5−8
3−4
0−2

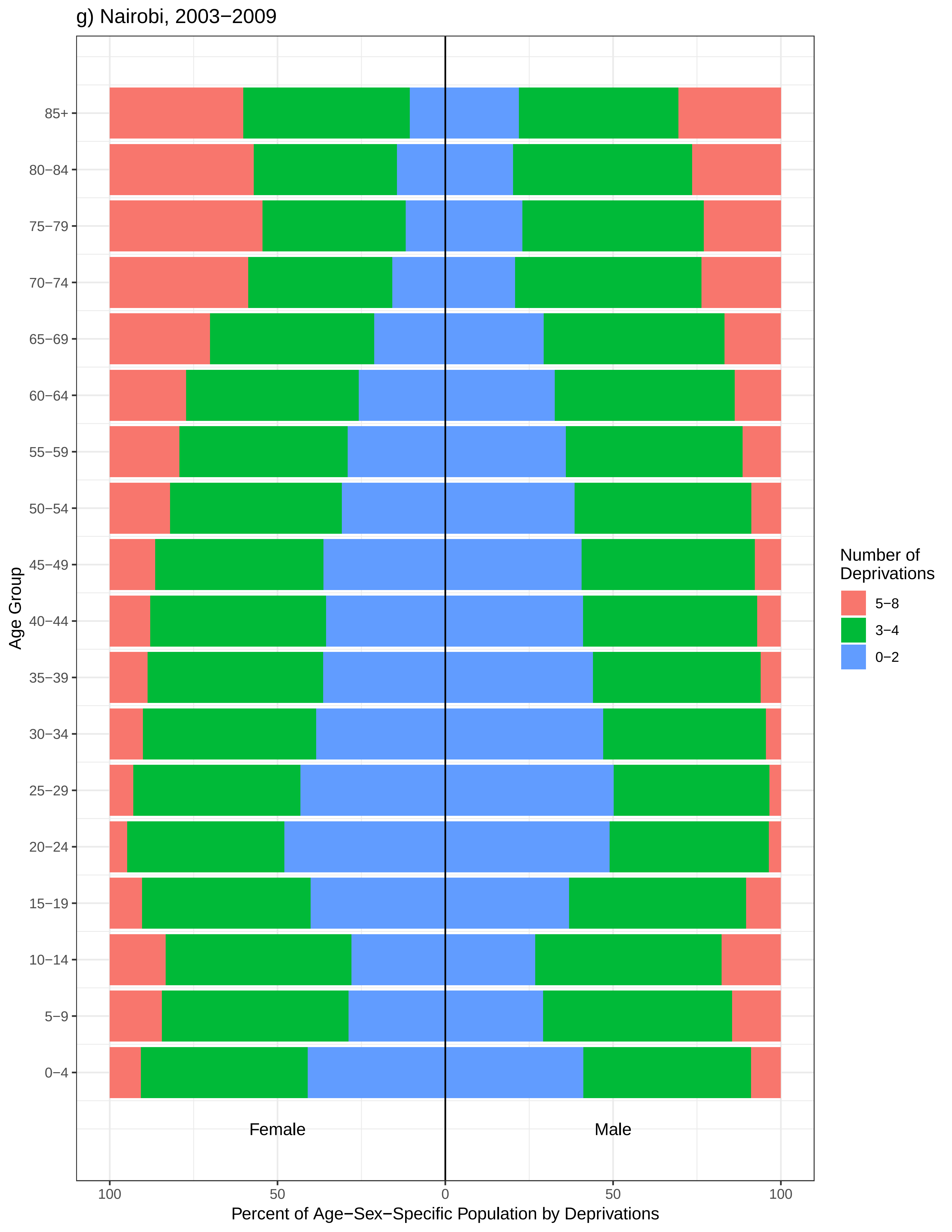

g) Nairobi, 2003−2009
Age Group
85+
80−84
75−79
70−74
65−69
60−64
55−59
50−54
45−49
40−44
35−39
30−34
25−29
20−24
15−19
10−14
5−9
0−4
Female
Male
100
50
0
50
100
Percent of Age−Sex−Specific Population by Deprivations
Number of Deprivations
5−8
3−4
0−2

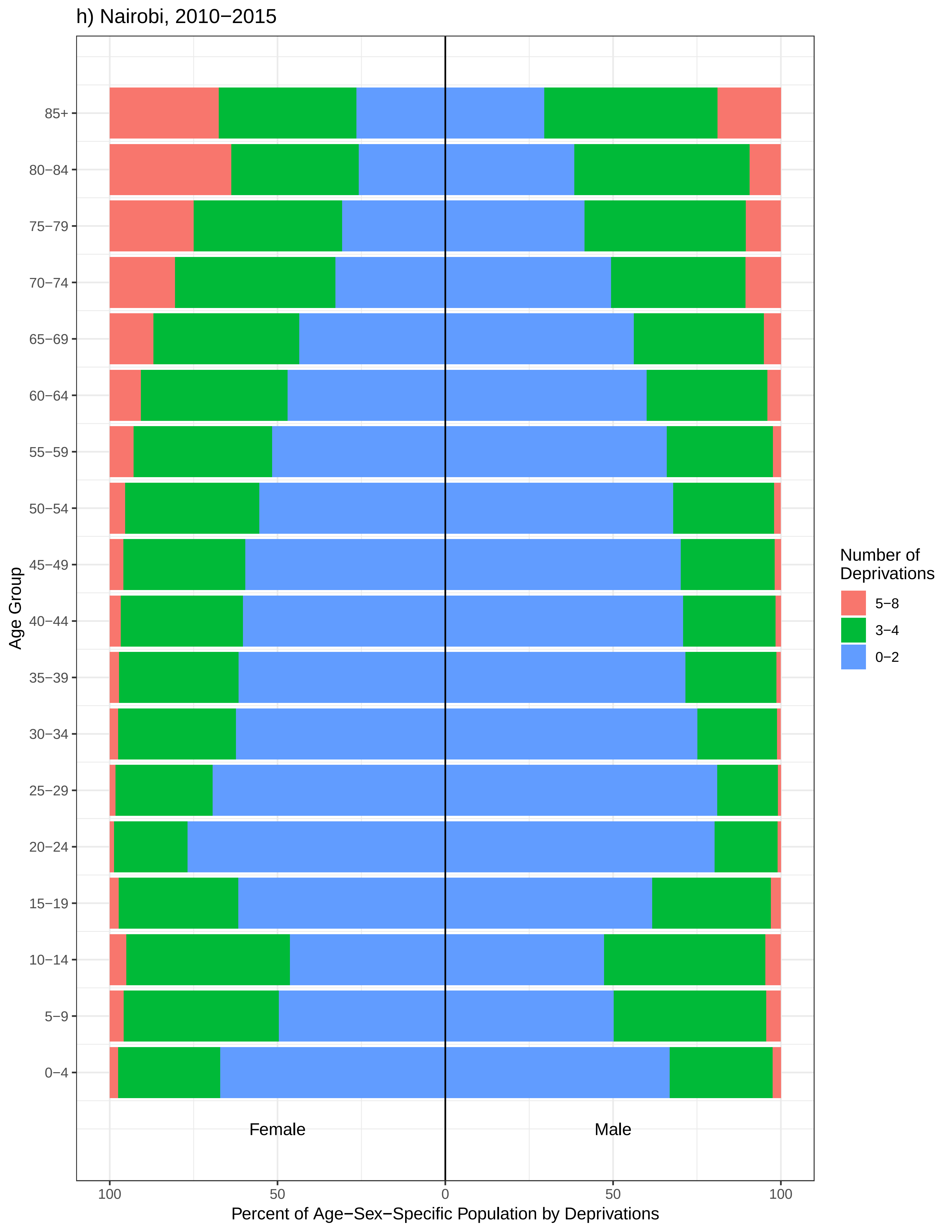

h) Nairobi, 2010−2015
85+
80−84
75−79
70−74
65−69
60−64
55−59
50−54
45−49
40−44
35−39
30−34
25−29
20−24
15−19
10−14
5−9
0−4
Age Group
Female
Male
100
50
0
50
100
Percent of Age−Sex−Specific Population by Deprivations
Number of Deprivations
5−8
3−4
0−2

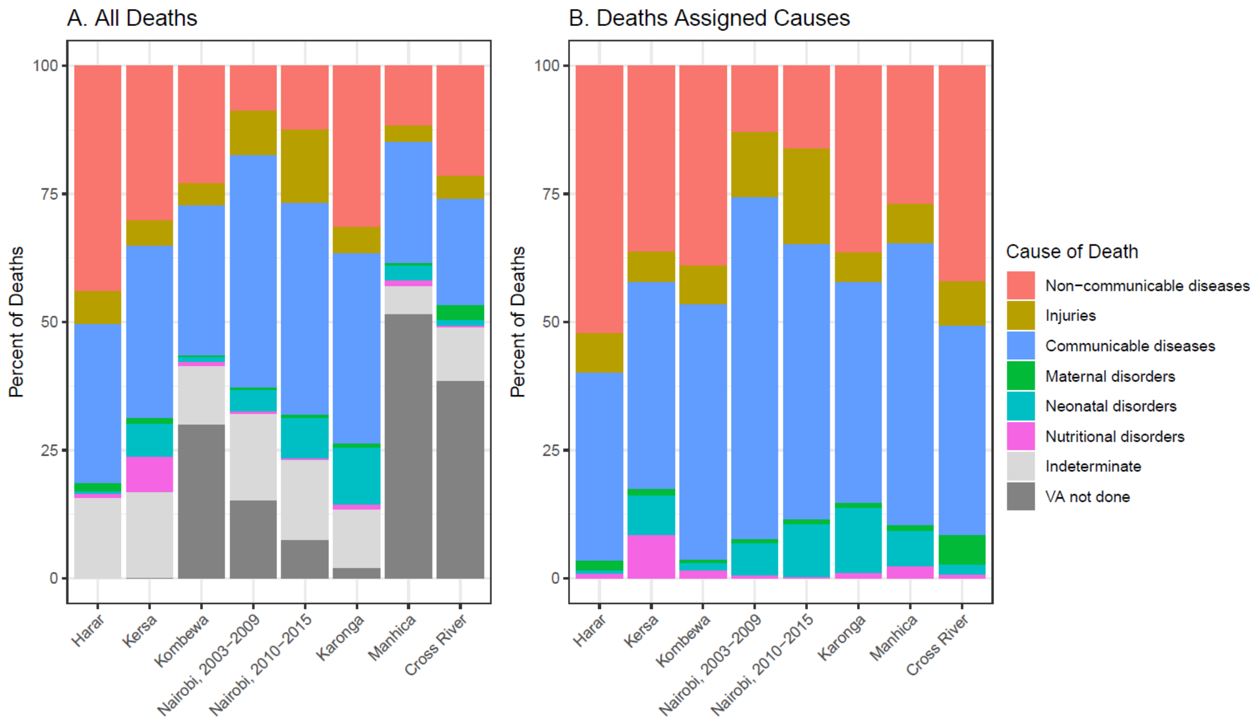

Figure S4: All-age proportions of broad causes of death among deaths among (a) all deaths and (b) deaths assigned causes by InterVA

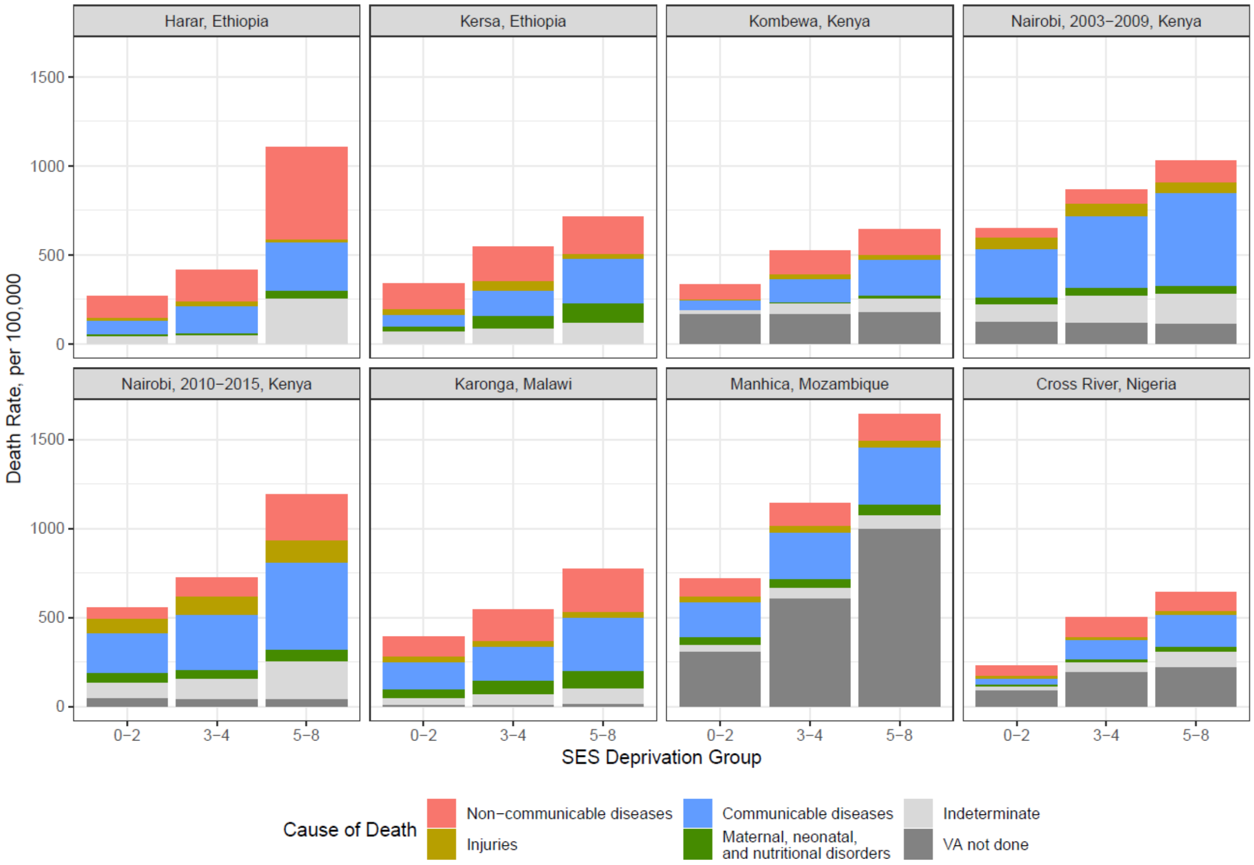

Figure S5: All-age mortality rates by site, socioeconomic group, and cause of death category

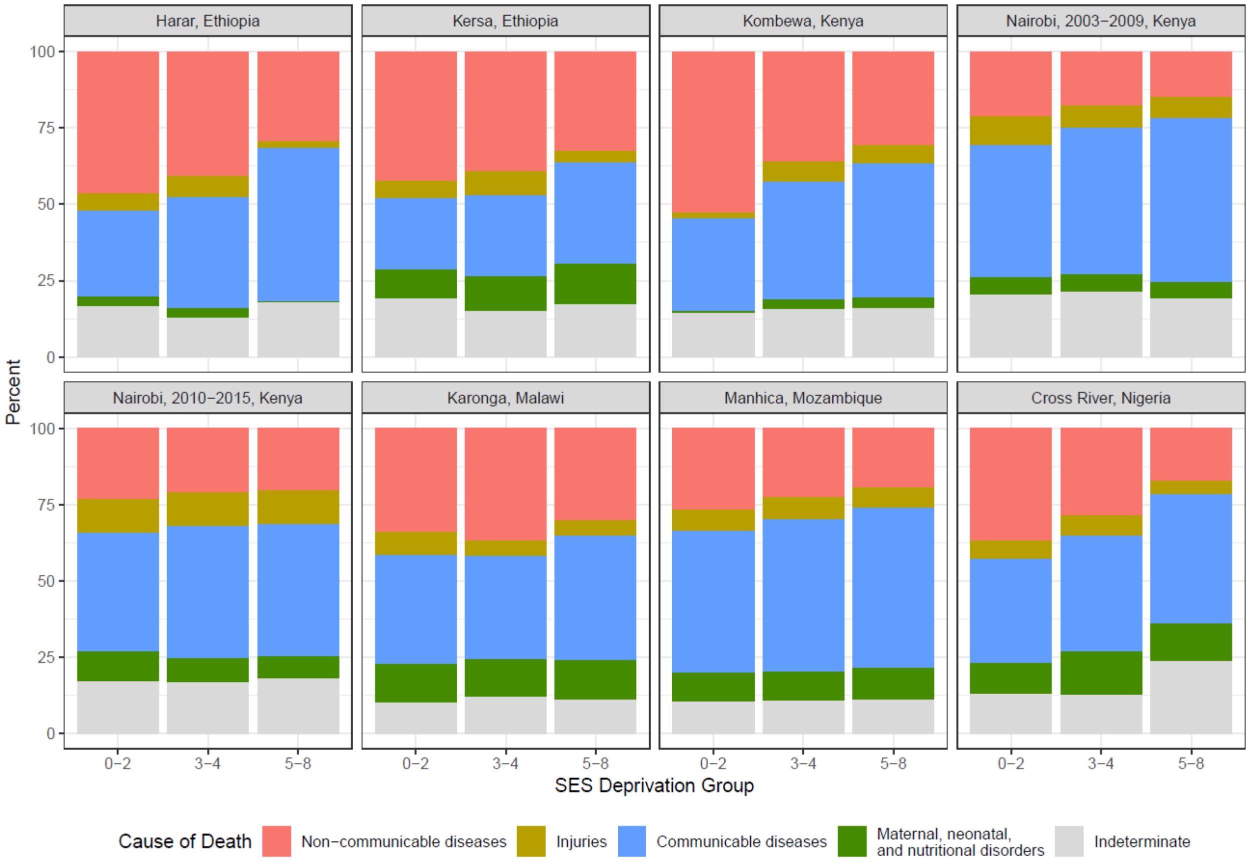

Figure S6: Age-sex-standardized death proportions by site, socioeconomic group, and cause of death category, excluding deaths for which VA not conducted

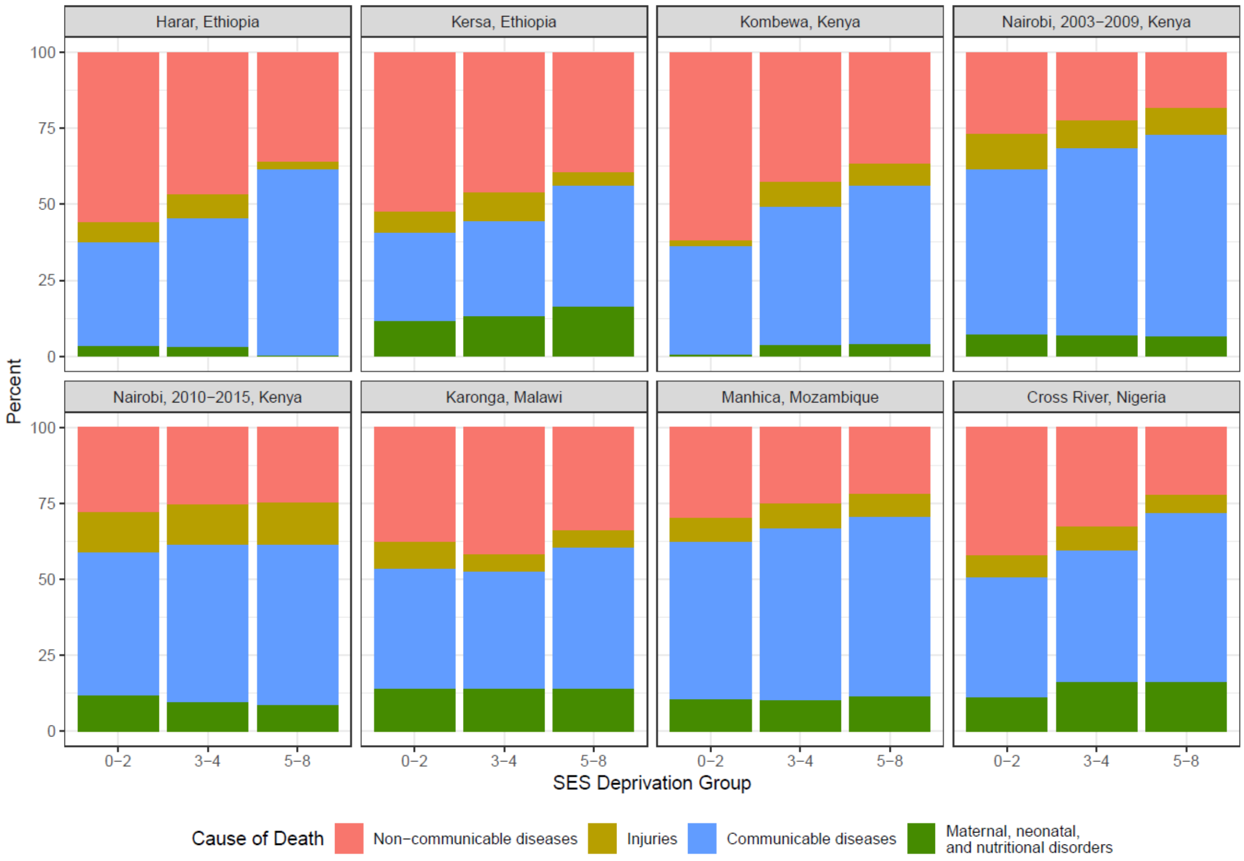

Figure S7: Age-sex-standardized death proportions by site, socioeconomic group, and cause of death category, excluding deaths for which VA not conducted or InterVA assigned cause of death is indeterminate

Table S7: Rate ratios for cause-specific death rates between SES groups, controlling for year, sex, and age (with 95% uncertainty intervals), 0-2 deprivations as reference group

Cause Name	Group	Harar	Kersa	Kombewa	Nairobi, 2003-2009	Nairobi, 2010-2015	Karonga	Manhiça	Cross River
**All causes**	3-4	1.36 (1.12,1.64)	1.61 (1.04,2.51)	1.65 (1.32,2.07)	1.33 (1.19,1.49)	1.42 (1.29,1.56)	1.28 (1.00,1.62)	1.34 (1.26,1.43)	2.18 (1.68,2.80)
	5-8	1.42 (0.85,2.31)	2.06 (1.34,3.17)	2.03 (1.63,2.53)	1.48 (1.27,1.74)	1.83 (1.48,2.26)	1.47 (1.16,1.84)	1.62 (1.50,1.74)	2.06 (1.52,2.77)
**Communicable, maternal, neonatal, and nutritional diseases**	3-4	1.73 (1.16,2.47)	2.23 (1.00,5.23)	2.73 (1.60,4.59)	1.41 (1.21,1.65)	1.43 (1.25,1.63)	1.21 (0.87,1.73)	1.22 (1.03,1.44)	2.69 (1.64,4.45)
	5-8	1.59 (0.56,3.97)	3.63 (1.66,8.34)	4.02 (2.37,6.82)	1.74 (1.41,2.19)	1.90 (1.38,2.59)	1.62 (1.17,2.27)	1.34 (1.11,1.62)	4.05 (2.34,7.11)
**Diarrheal disease**	3-4	1.23 (0.29,5.24)	^	^	1.48 (0.66,3.36)	1.17 (0.48,2.80)	3.22 (0.27,139.45)	1.12 (0.75,1.69)	2.36 (0.28,20.15)
	5-8	^	^	^	1.98 (0.60,6.16)	1.51 (0.20,11.01)	2.77 (0.22,112.73)	1.53 (0.91,2.56)	^
**HIV/AIDS**	3-4	2.09 (1.24,3.52)	^	5.80 (1.41,26.25)	1.52 (1.06,2.21)	1.70 (1.15,2.55)	0.98 (0.52,1.93)	1.46 (1.04,2.06)	2.53 (0.71,8.54)
	5-8	2.33 (0.40,9.49)	^	8.34 (2.04,38.09)	1.62 (1.04,2.59)	2.63 (1.25,5.03)	1.40 (0.74,2.67)	1.76 (1.26,2.44)	5.43 (1.49,19.80)
**Lower respiratory infection**	3-4	1.06 (0.50,2.22)	1.13 (0.27,4.96)	5.33 (1.31,22.66)	1.11 (0.78,1.58)	1.28 (0.92,1.78)	1.04 (0.52,2.19)	1.22 (0.97,1.52)	1.66 (0.53,5.17)
	5-8	0.51 (0.03,7.10)	1.77 (0.45,7.38)	7.86 (1.91,33.15)	1.42 (0.87,2.31)	0.90 (0.31,2.38)	1.55 (0.77,3.22)	1.15 (0.87,1.52)	4.03 (1.33,12.25)
**Malaria**	3-4	1.92 (0.43,8.12)	1.30 (0.10,16.85)	0.88 (0.39,2.06)	1.56 (0.64,3.96)	1.05 (0.40,2.71)	1.92 (0.72,5.58)	1.52 (1.16,2.02)	3.79 (1.32,11.02)
	5-8	^	2.03 (0.17,24.93)	1.51 (0.67,3.38)	1.48 (0.38,5.52)	1.89 (0.32,10.05)	2.69 (1.02,7.69)	2.12 (1.52,2.96)	4.45 (1.29,15.21)
**Tuberculosis**	3-4	2.87 (1.24,6.78)	1.22 (0.16,9.58)	1.62 (0.59,4.47)	1.55 (0.90,2.66)	1.46 (1.13,1.90)	0.91 (0.27,3.24)	1.20 (0.82,1.71)	17.95 (2.13,144.78)
	5-8	^	2.61 (0.37,18.56)	2.08 (0.75,5.63)	2.18 (1.23,3.87)	2.07 (1.04,3.70)	1.28 (0.39,4.66)	1.35 (0.79,2.20)	14.86 (1.46,147.00)
**Other infectious diseases**	3-4	1.75 (0.67,4.43)	^	^	1.33 (0.91,1.90)	1.75 (1.21,2.59)	1.43 (0.40,6.26)	1.28 (0.73,2.28)	1.44 (0.23,8.77)
	5-8	3.19 (0.43,18.67)	^	^	1.70 (0.98,2.88)	2.60 (1.22,5.27)	1.93 (0.54,8.36)	1.74 (0.84,3.57)	2.86 (0.44,18.77)
**Maternal disorders**	3-4	0.95 (0.21,4.39)	^	^	1.55 (0.39,6.56)	0.91 (0.27,3.34)	^	1.01 (0.53,1.94)	0.73 (0.11,4.31)
	5-8	^	^	^	1.44 (0.12,10.01)	2.96 (0.26,19.51)	^	0.66 (0.18,2.18)	3.46 (0.79,14.40)

**Neonatal disorders**	3-4	1.37 (0.12,17.34)	2.31 (0.31,18.23)	^	1.13 (0.68,1.90)	1.02 (0.73,1.41)	1.25 (0.59,2.97)	0.97 (0.59,1.58)	7.69 (0.60,96.11)
	5-8	^	2.85 (0.41,21.12)	^	1.12 (0.50,2.34)	1.11 (0.41,2.84)	1.43 (0.70,3.30)	1.28 (0.84,1.90)	^
**Anemia and malnutrition**	3-4	1.44 (0.16,12.50)	1.46 (0.19,11.28)	^	0.98 (0.21,4.32)	7.41 (0.78,74.33)	0.81 (0.06,10.60)	1.35 (0.84,2.18)	^
	5-8	4.79 (0.37,65.96)	3.28 (0.45,24.20)	^	1.56 (0.20,10.56)	^	2.16 (0.17,25.62)	1.21 (0.59,2.45)	4.23 (0.12,160.33)
**Indeterminate**	3-4	0.98 (0.59,1.64)	1.27 (0.49,3.39)	2.69 (1.23,5.77)	1.37 (1.10,1.71)	1.40 (1.11,1.78)	1.31 (0.67,2.64)	1.38 (1.12,1.70)	2.89 (1.33,6.21)
	5-8	1.82 (0.70,4.57)	1.69 (0.67,4.38)	3.63 (1.67,7.67)	1.38 (0.98,1.92)	2.06 (1.26,3.35)	1.38 (0.70,2.81)	1.55 (1.18,2.03)	3.48 (1.44,8.47)
**Injuries**	3-4	1.97 (0.97,4.09)	1.64 (0.40,6.74)	8.29 (1.08,61.68)	1.18 (0.87,1.57)	1.53 (1.08,2.18)	1.03 (0.44,2.63)	1.30 (0.99,1.70)	1.47 (0.47,4.64)
	5-8	^	1.07 (0.28,4.28)	9.91 (1.30,72.54)	1.19 (0.63,2.20)	2.03 (0.99,3.83)	1.19 (0.50,3.00)	1.37 (0.92,2.01)	2.11 (0.50,8.45)
**Transport injuries**	3-4	1.93 (0.71,5.33)	1.56 (0.26,10.41)	2.49 (0.31,19.39)	1.12 (0.47,2.65)	1.64 (0.54,4.89)	0.51 (0.16,1.65)	1.12 (0.78,1.58)	1.20 (0.20,6.33)
	5-8	^	0.70 (0.11,4.34)	4.16 (0.54,32.48)	1.34 (0.49,3.65)	2.30 (0.62,8.08)	0.71 (0.23,2.23)	0.88 (0.50,1.53)	2.54 (0.44,13.90)
**Intentional injuries**	3-4	2.63 (0.68,9.93)	0.98 (0.10,8.56)	^	1.30 (0.76,2.32)	1.44 (0.95,2.21)	1.66 (0.27,15.28)	2.72 (1.36,5.55)	2.38 (0.16,36.95)
	5-8	^	1.03 (0.13,8.27)	^	1.19 (0.45,2.98)	2.29 (0.65,6.78)	1.67 (0.27,15.57)	3.94 (1.62,9.19)	^
**Unintentional injuries**	3-4	1.41 (0.29,7.44)	^	^	1.15 (0.51,2.50)	1.60 (1.01,2.57)	^	1.10 (0.55,2.22)	1.55 (0.24,9.88)
	5-8	^	^	^	0.91 (0.21,3.71)	1.23 (0.26,4.43)	^	1.41 (0.60,3.24)	1.86 (0.14,24.21)
**Other injuries**	3-4	^	^	^	0.92 (0.23,3.72)	1.29 (0.41,4.03)	^	1.05 (0.27,4.02)	NA
	5-8	^	^	^	0.98 (0.07,7.81)	3.44 (0.55,21.61)	^	2.61 (0.52,11.45)	NA
**Non-communicable diseases**	3-4	1.18 (0.76,1.81)	1.37 (0.70,2.76)	1.67 (1.11,2.52)	1.41 (1.00,2.02)	1.45 (1.10,1.91)	1.51 (1.01,2.32)	1.10 (0.95,1.28)	1.90 (1.12,3.21)
	5-8	1.17 (0.60,2.18)	1.47 (0.77,2.89)	1.93 (1.30,2.91)	1.29 (0.82,2.05)	1.60 (0.99,2.58)	1.41 (0.94,2.18)	1.14 (0.92,1.41)	1.15 (0.57,2.35)
**Cardiovascular diseases**	3-4	1.29 (0.55,3.06)	1.97 (0.31,14.68)	2.00 (0.76,5.14)	1.52 (0.66,3.54)	1.62 (0.67,3.99)	1.38 (0.65,2.98)	1.00 (0.79,1.28)	1.48 (0.49,4.26)
	5-8	1.70 (0.71,3.98)	1.63 (0.27,11.65)	1.81 (0.69,4.78)	1.20 (0.51,2.79)	1.35 (0.55,3.29)	1.05 (0.49,2.24)	0.85 (0.60,1.20)	0.69 (0.19,2.38)
**Chronic respiratory diseases**	3-4	0.24 (0.05,1.09)	0.65 (0.07,6.69)	0.55 (0.19,1.55)	0.83 (0.28,2.68)	2.21 (0.66,7.26)	1.13 (0.35,3.96)	0.99 (0.53,1.78)	^

	5-8	^	1.28 (0.15,11.03)	0.43 (0.16,1.23)	450.59 (<0.01,9.81)	3.16 (0.41,22.62)	0.83 (0.25,2.95)	1.27 (0.57,2.83)	^
**Diabetes**	3-4	1.38 (0.48,3.97)	^	1.24 (0.22,6.60)	^	1.02 (0.16,6.02)	1.03 (0.22,4.63)	0.83 (0.33,2.19)	0.84 (0.13,5.37)
	5-8	1.80 (0.23,10.76)	^	1.52 (0.28,7.88)	^	^	0.80 (0.17,3.77)	0.97 (0.33,2.61)	^
**Reproductive neoplasms**	3-4	0.67 (0.15,2.61)	^	1.01 (0.22,4.61)	1.14 (0.28,4.68)	1.20 (0.30,4.45)	2.29 (0.30,19.39)	1.02 (0.46,2.28)	1.72 (0.06,49.17)
	5-8	^	^	1.05 (0.23,4.68)	^	1.63 (0.18,10.56)	2.41 (0.32,21.02)	0.94 (0.30,2.69)	4.06 (0.16,111.54)
**Respiratory neoplasms**	3-4	0.78 (0.14,4.37)	0.99 (0.13,7.97)	^	2.17 (0.79,6.71)	1.63 (0.62,4.36)	4.08 (0.15,97.75)	1.45 (0.63,3.38)	^
	5-8	^	0.83 (0.11,6.84)	^	2.81 (0.79,10.94)	1.11 (0.13,7.47)	4.08 (0.16,101.82)	1.80 (0.62,5.09)	^
**Other neoplasms**	3-4	1.33 (0.69,2.65)	2.06 (0.56,8.03)	2.09 (0.81,5.50)	0.88 (0.32,2.54)	1.69 (0.78,3.69)	1.63 (0.52,5.31)	1.27 (0.77,2.09)	2.17 (0.82,5.79)
	5-8	0.03 (<0.01,70.11)	1.96 (0.52,7.64)	3.12 (1.22,8.07)	0.46 (0.03,3.84)	1.67 (0.32,7.51)	1.53 (0.47,4.89)	1.82 (1.00,3.31)	1.41 (0.40,5.17)
**Acute abdomen, liver cirrhosis, renal failure**	3-4	1.27 (0.29,5.53)	4.69 (0.29,68.22)	1.55 (0.70,3.44)	1.55 (0.70,3.60)	1.10 (0.62,1.97)	1.33 (0.57,3.16)	1.26 (0.95,1.67)	2.07 (0.56,7.50)
	5-8	2.01 (0.43,9.05)	5.65 (0.38,79.54)	1.81 (0.84,3.91)	2.63 (0.95,7.17)	1.79 (0.60,5.18)	1.51 (0.66,3.55)	1.23 (0.80,1.85)	3.06 (0.74,13.07)
**Epilepsy**	3-4	4.25 (0.35,64.26)	^	^	1.81 (0.18,24.44)	0.87 (0.15,4.15)	^	1.33 (0.67,2.59)	4.58 (0.09,235.93)
	5-8	^	^	^	2.64 (0.08,62.27)	6.36 (0.93,42.49)	^	1.84 (0.75,4.29)	^
**Other non-communicable diseases**	3-4	^	0.37 (0.10,1.95)	^	^	^	^	0.82 (0.47,1.44)	^
	5-8	^	0.40 (0.13,1.77)	^	^	^	^	1.23 (0.61,2.46)	^
**VA not done**	3-4	NA	^	1.05 (0.77,1.45)	1.14 (0.90,1.43)	1.15 (0.77,1.66)	0.65 (0.17,2.85)	1.48 (1.37,1.61)	2.06 (1.43,3.00)
	5-8	NA	^	1.10 (0.80,1.50)	1.08 (0.69,1.64)	0.91 (0.23,2.60)	0.82 (0.21,3.44)	1.84 (1.66,2.03)	1.50 (0.93,2.43)

^ Indicates estimates uninformative given infrequency of cause (estimates with uncertainty intervals ranging below 0.01 to over 1000). NA indicates no model run because no deaths occurred in the given site from the given cause.

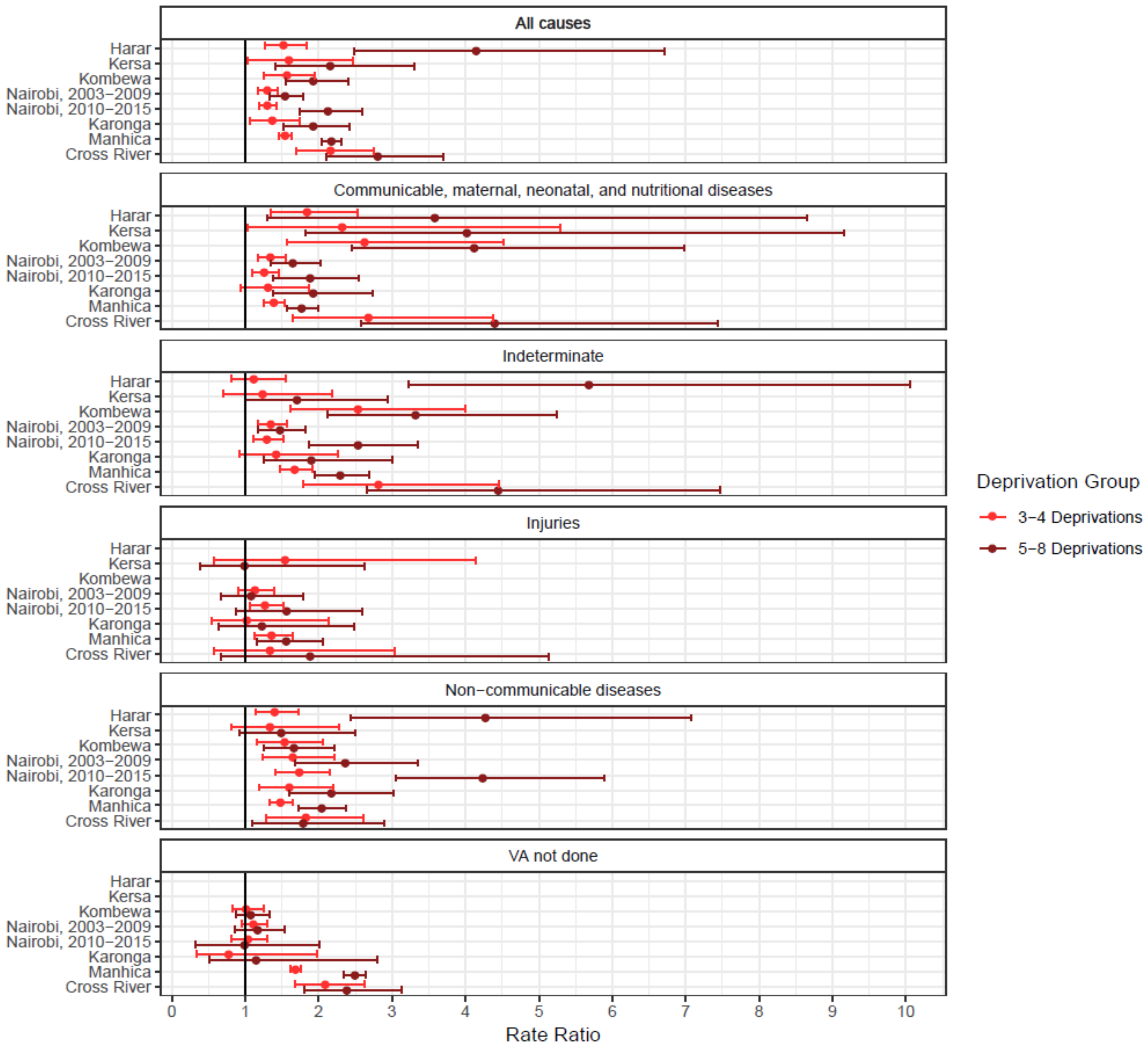

Ratios estimated using negative binomial regressions, controlling for sex and year. Kombewa and Harar HDSS deaths from injuries omitted from graph for scale (Kombewa: 3-4 deprivation group: 7·6 [1·9-31·9], 5-8 deprivation group: 8·6 [2·2-36·8]; Harar: 3-4 deprivation group: 1·9 [1·1-3·3], 5-8 deprivation group: estimates unstable due to small number of deaths). Harar and Kersa HDSS deaths with no verbal autopsy omitted because almost every death had a verbal autopsy.

Figure S8: All-age mortality rate ratios by level of deprivation for broad causes of death: higher deprivations compared to the fewest deprivations (0-2)

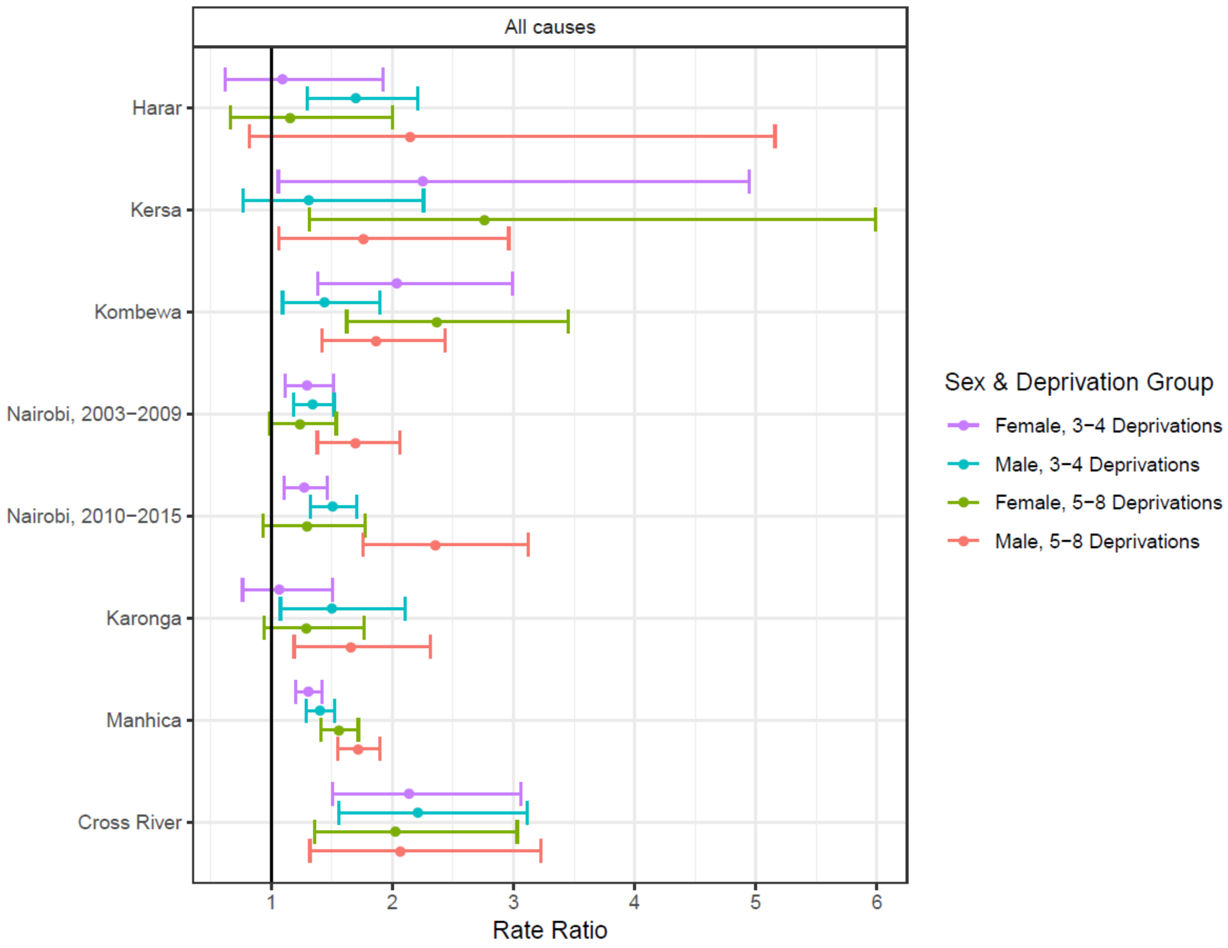

Figure S9: All-cause mortality rate ratios by level of deprivation: higher deprivations compared to the least deprivations (0-2), for males and females

Mortality rate ratios in Figure S8 generated by negative binomial regressions stratified by sex, controlling for year and age groups. To test for differences between the estimated SES group mortality rate ratios for men and women, we made pairwise comparisons of 10,000 random draws of the estimated rate ratios for men and women. There were three site-groups with less than 5% of draws of rate ratios (comparing the 3-4 and 5-8 deprivation groups to the 0-2 deprivation group) that were lower in men than women: Nairobi 2010-2015, 5-8 deprivations (0.22%); Nairobi 2010-2015, 3-4 deprivations (4.19%); and Nairobi 2003-2009, 5-8 deprivations (2.09%). While other sites did show some consistent patterns (e.g. male relative rate point estimates and ranges were higher than those in women in the same deprivation groups in Manhiça), there were no other site-groups that met the 5% criterion indicating difference in the degree of inequality by sex.

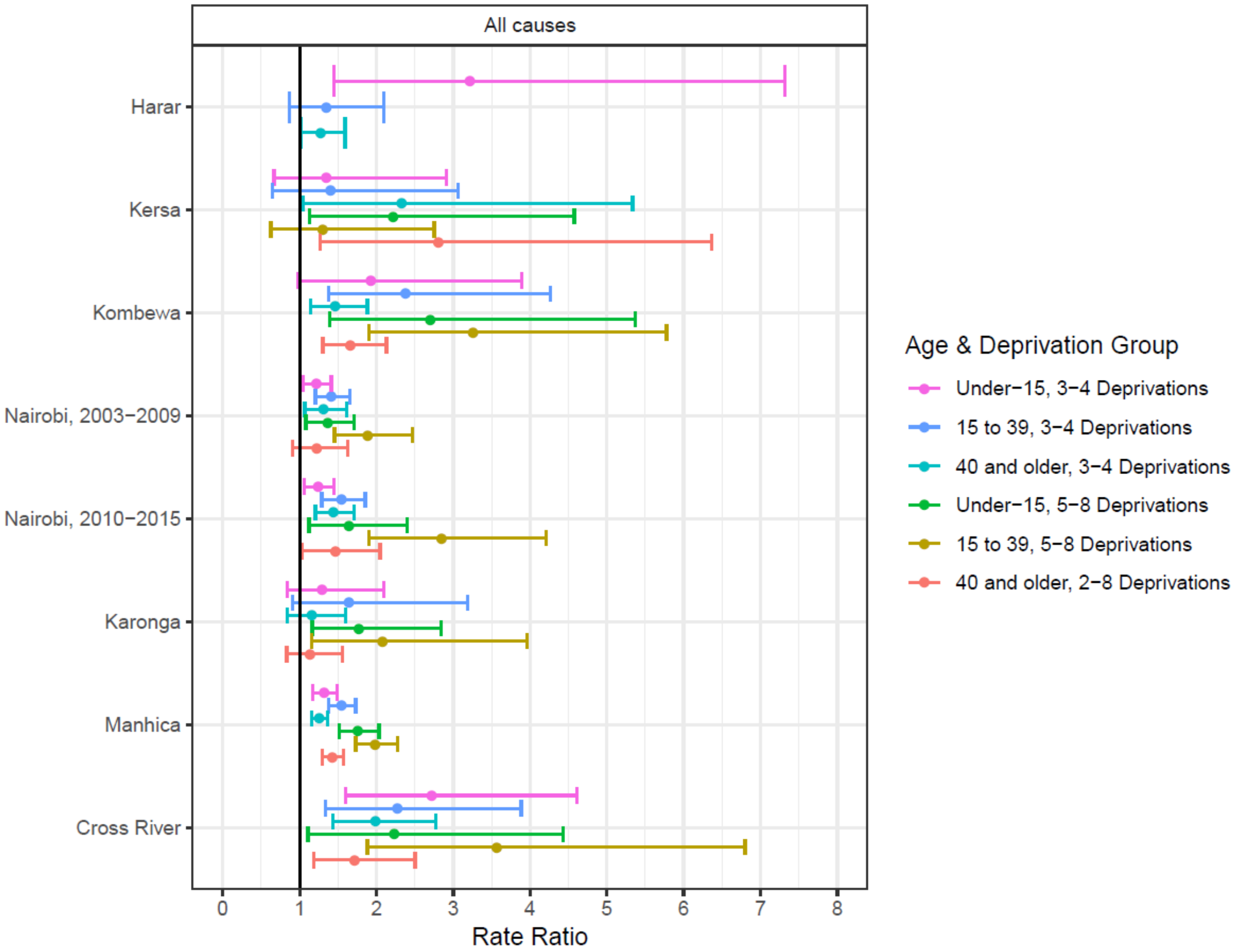

Estimates from the Harar site for the 5-8 deprivation group omitted because of small numbers of death above age 15 in the 5-8 deprivation group.

Figure S10: All-cause mortality rate ratios by level of deprivation: higher deprivations compared to the least deprivations (0-2), for age groups under-15, 15 to 39, and 40 and older

Mortality rate ratios in Figure S9 generated by negative binomial regressions stratified by broad age groups, controlling for year, sex, and age groups within the broader age ranges. Similar to tests of the difference in the degree of mortality disparities by sex, we examined differences between the estimated SES group mortality rate ratios across age groups by making pairwise comparisons of 10,000 random draws of the estimated rate ratios between each age group. There were two different patterns of differences in different sites. First, some sites showed larger SES-related relative mortality disparities at young ages. In the Harar site, the mortality rate ratio for the 3-4 deprivation group compared to the 0-2 deprivation group was larger under age 15 than in the other two age groups (96.78% of draws higher compared to 15 to 39 age group, 98.59% of draws higher compared to 40 plus age group). In the Karonga and Manhiça sites, the 5-8 deprivation group showed larger mortality rate ratios compared to the 0-2 deprivation group in the under-15 age group compared to the over-40 age group (95.08% of draws and 99.02% of draws higher, respectively). Additionally, some sites showed lower mortality rate ratios in ages 15 to 39 than in other age groups. In the Nairobi site in 2010-2015, the mortality rate ratios were higher in ages 15 to 39 than under age 15 for both the 3-4 deprivation group (96.42% of draws) and the 5-8 deprivation group (97.55% of draws), compared to the 0-2 deprivation group. In the Nairobi site in 2003-2009, this was true of the 5-8 deprivation group as well (96.64% of draws). Meanwhile, the 15 to 39 age group had higher rate ratios between SES groups than the 40 and older age group in many site-groups: Manhiça, 3-4 deprivations (99.84%); Cross River, 5-8 deprivations (97.47%); Karonga, 5-8 deprivations (95.94%); Kombewa, 5-8 deprivations (98.71%); Manhiça, 5-8 deprivations (99.99%); Nairobi 2003-2009, 5-8 deprivations (98.66%); and Nairobi 2010-2015, 5-8 deprivations (99.42%).

Sensitivity Analyses

While the sites in Nairobi, Manhiça, and Karonga had SES surveys at multiple time points, the sites in Harar, Kersa, Cross River, and Kombewa only had SES data from a survey at one time point, along with data for those who newly moved in to the site. We decided to use the most detailed data possible rather than using only the data from the first available year for the sites in Nairobi, Manhiça, and Karonga. To build evidence around the differences we might have seen in the results for the sites in Harar, Kersa, Cross River, and Kombewa if we had multiple recurring observations of SES data, we compared the results in Nairobi, Manhiça, and Karonga under two assumptions. In both assumptions, we used the imputation that included longitudinal data. In the first, as is in the main text, we used the estimates of the number of deprivations a household experiences on our poverty index that could vary over years. In the second assumption, we simply took the values of the poverty index from the first year of data for each site (or in the case of Nairobi, for the two time periods we split), and assigned the household those poverty index values for the whole time series. While there were differences, they were not large, and the results were qualitatively similar. When households were assigned the poverty index value from the beginning of the time period, the proportion of person-years (and deaths) in the site lived with more deprivations on the poverty index was higher. This is the result of the gradual reduction in poverty on our index that we observed in these sites over time. The pattern of death rates by SES groups remained similar in each site. The largest difference was in Nairobi 2003-2009, where the disparity grew slightly smaller when using the time-varying SES measures. In Manhiça and Karonga, the disparity in the overall death rate grew slightly larger.

Figure S11: Age-sex-standardized mortality rates by socioeconomic group and cause of death category, comparing SES assumptions

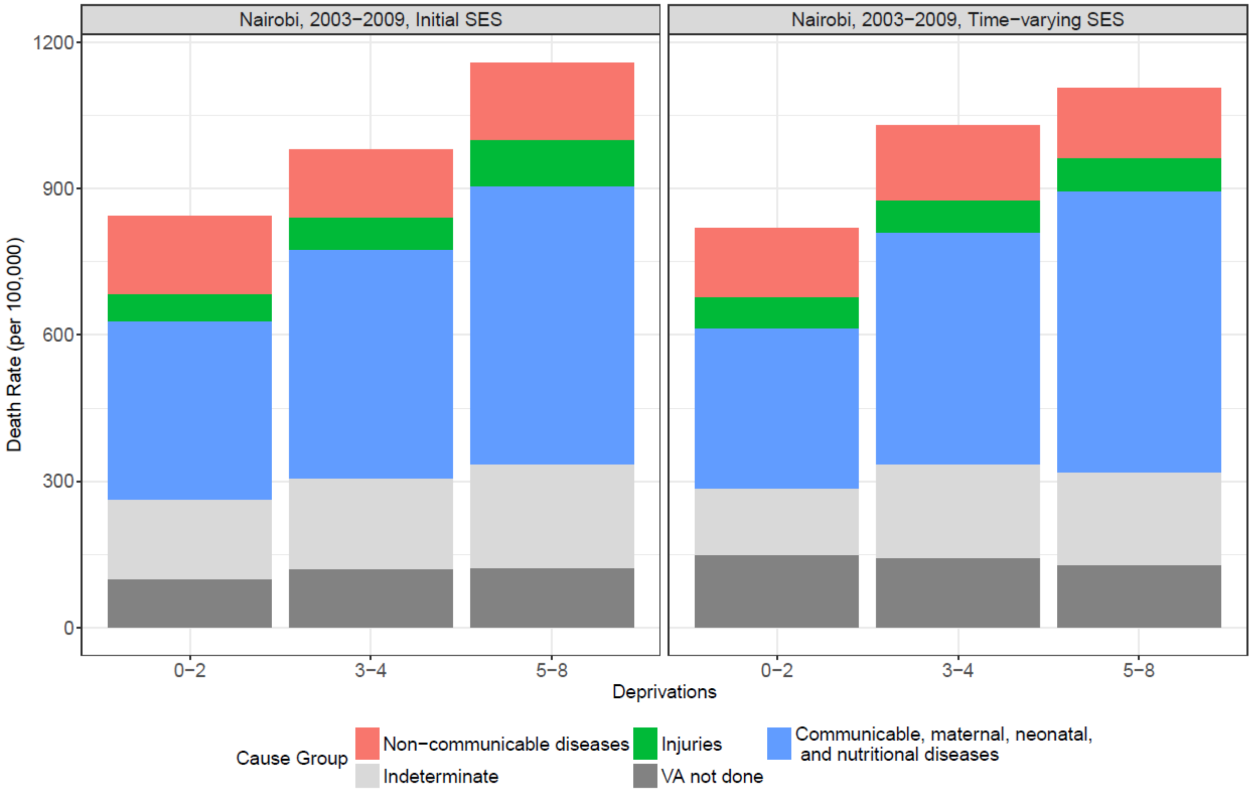

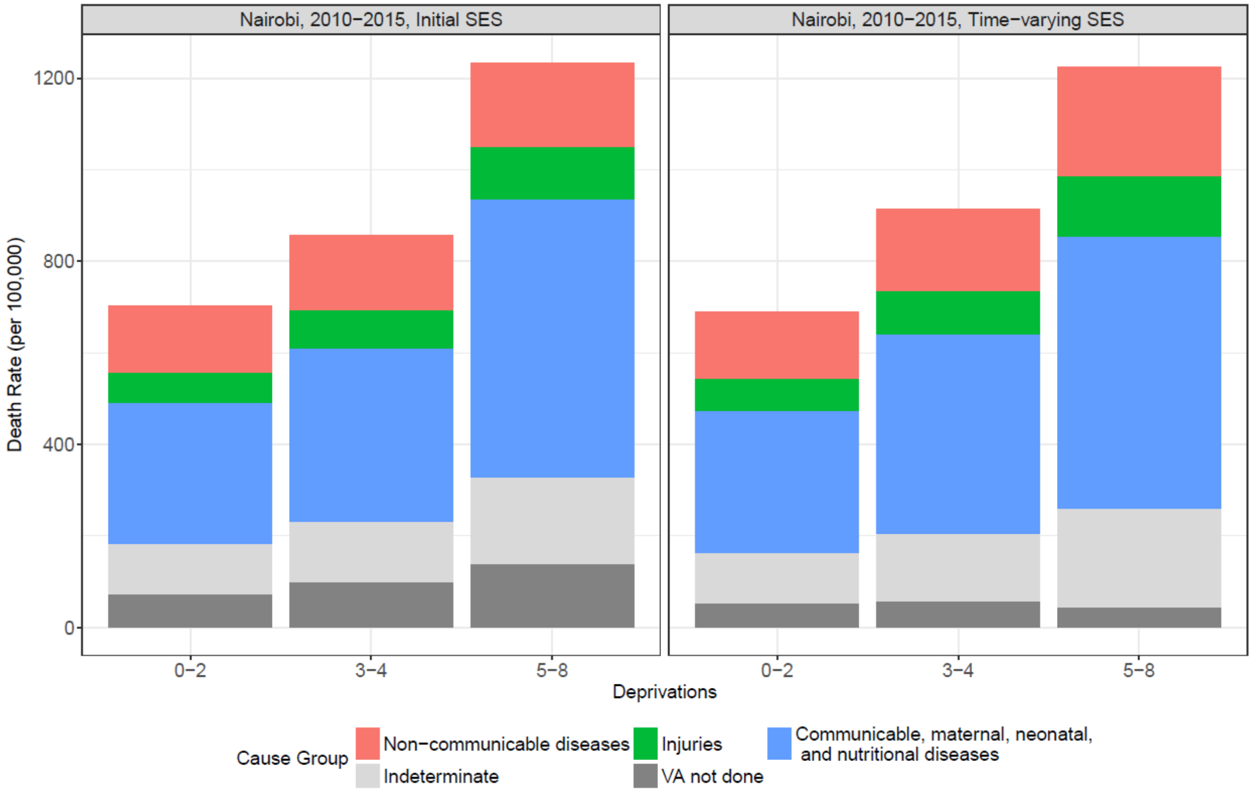
Nairobi, 2010−2015, Initial SES
Nairobi, 2010−2015, Time−varying SES
Death Rate (per 100,000)
0
400
800
1200
0−2
3−4
5−8
Deprivations
Cause Group
Non−communicable diseases
Injuries
Communicable, maternal, neonatal, and nutritional diseases
Indeterminate
VA not done

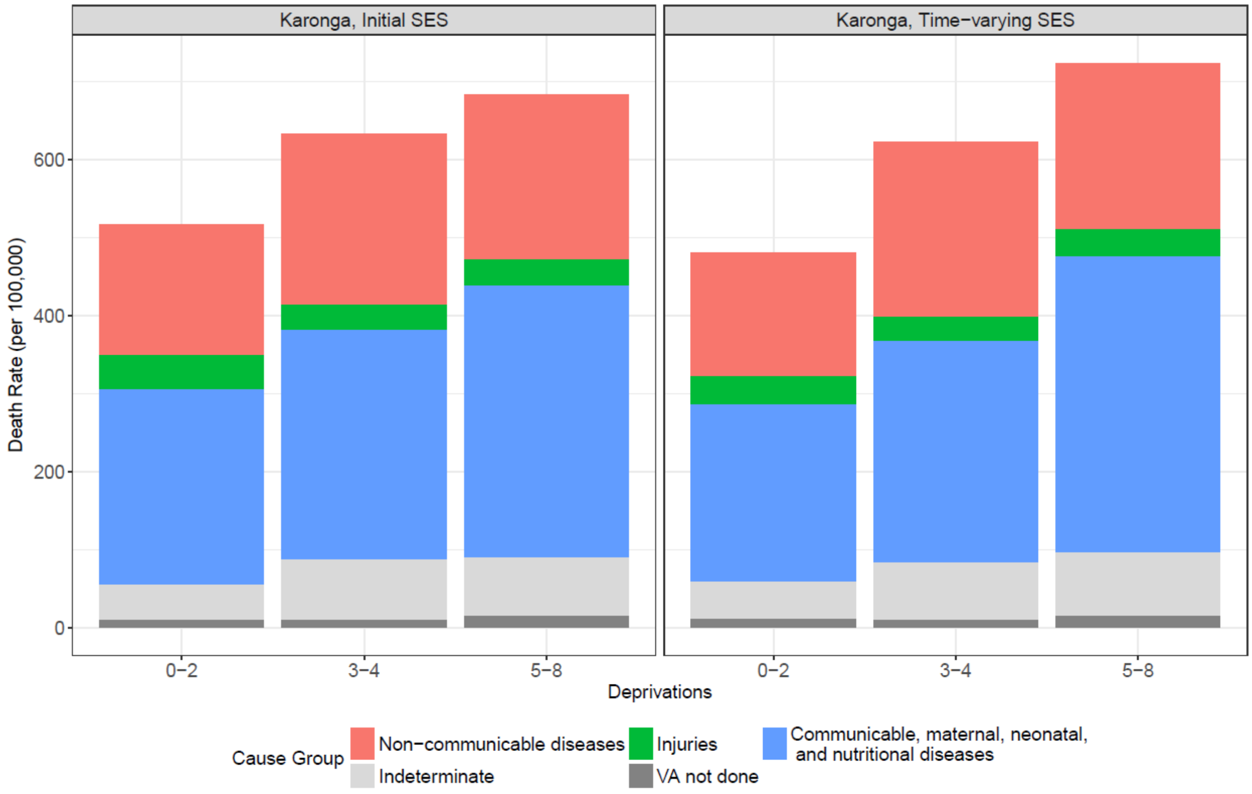
Karonga, Initial SES
Karonga, Time−varying SES
Death Rate (per 100,000)
0
200
400
600
0−2
3−4
5−8
Deprivations
Cause Group
Non−communicable diseases
Injuries
Communicable, maternal, neonatal, and nutritional diseases
Indeterminate
VA not done

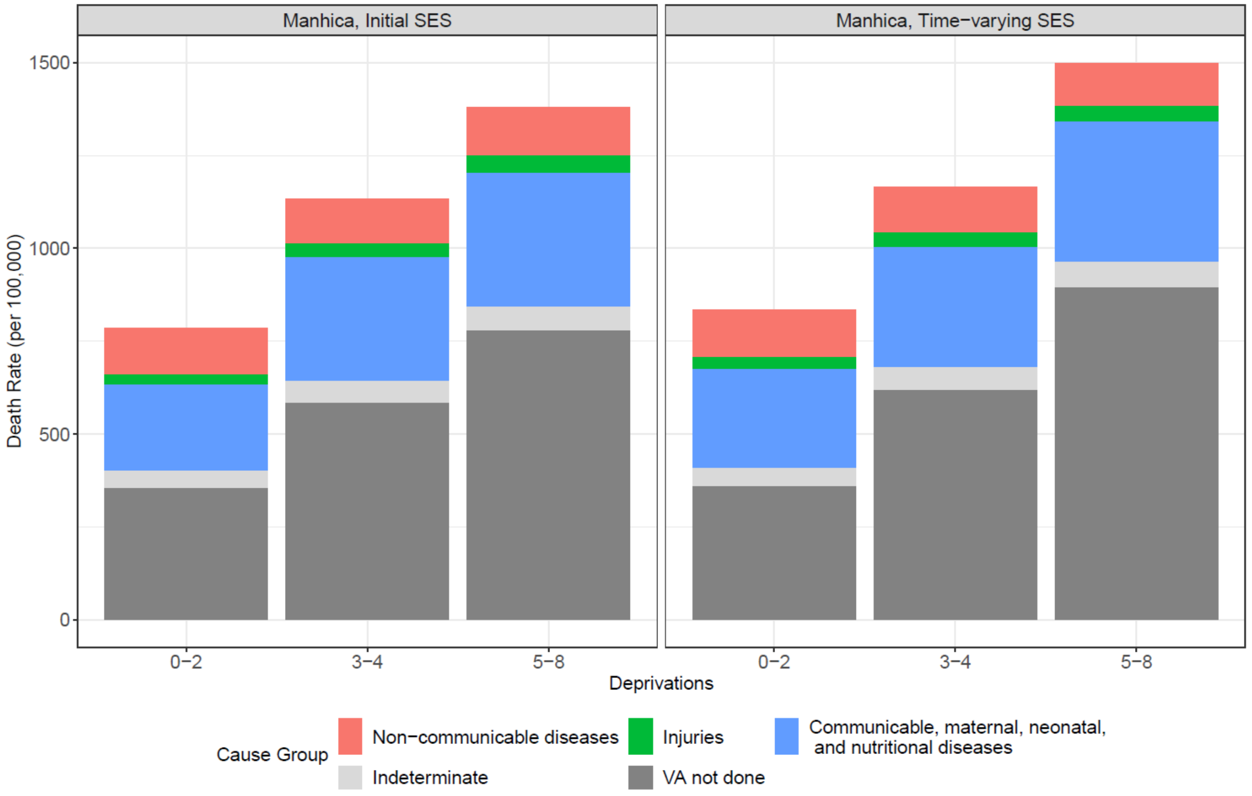

References

1. Alkire S, Santos ME. Measuring Acute Poverty in the Developing World: Robustness and Scope of the Multidimensional Poverty Index. World Dev. 2014;59: 251–274. doi:10.1016/j.worlddev.2014.01.026

2. Honaker J, King G, Blackwell M. Amelia II: A Program for Missing Data. J Stat Softw. 2011;45: 1–47. doi:10.18637/jss.v045.i07

3. Preston S, Heuveline P, Guillot M. Demography: Measuring and Modeling Population Processes. Wiley; 2000.
